# Motor function improvement and acceptability of non-invasive brain stimulation in patients with Parkinson's disease: a Bayesian network analysis

**DOI:** 10.3389/fnins.2023.1212640

**Published:** 2023-07-26

**Authors:** Youjia Qiu, Ziqian Yin, Menghan Wang, Aojie Duan, Minjia Xie, Jiang Wu, Zhong Wang, Gang Chen

**Affiliations:** ^1^Department of Neurosurgery & Brain and Nerve Research Laboratory, The First Affiliated Hospital of Soochow University, Suzhou, China; ^2^Suzhou Medical College of Soochow University, Suzhou, China

**Keywords:** Parkinson's disease, repetitive transcranial magnetic stimulation, transcranial direct current stimulation, non-invasive brain stimulation, meta-analysis

## Abstract

**Background:**

Parkinson's disease (PD) is a neurodegenerative disorder defined by progressive motor and non-motor symptoms. Currently, the pro-cognitive effects of transcranial direct current stimulation (tDCS) and repetitive transcranial magnetic stimulation (rTMS) are well-supported in previous literatures. However, controversy surrounding the optimal therapeutic target for motor symptom improvement remains.

**Objective:**

This network meta-analysis (NMA) was conducted to comprehensively evaluate the optimal strategy to use rTMS and tDCS to improve motor symptoms in PD.

**Methods:**

We searched PubMed, Embase, and Cochrane electronic databases for eligible randomized controlled studies (RCTs). The primary outcome was the changes of Unified Parkinson's Disease Rating Scale (UPDRS) part III score, the secondary outcomes were Time Up and Go Test (TUGT) time, and Freezing of Gait Questionnaire (FOGQ) score. The safety outcome was indicated by device-related adverse events (AEs).

**Result:**

We enrolled 28 studies that investigated various strategies, including high-frequency rTMS (HFrTMS), low-frequency rTMS (LFrTMS), anodal tDCS (AtDCS), AtDCS_ cathode tDCS (CtDCS), HFrTMS_LFrTMS, and Sham control groups. Both HFrTMS (short-term: mean difference (MD) −5.21, 95% credible interval (CrI) −9.26 to −1.23, long-term: MD −4.74, 95% CrI −6.45 to −3.05), and LFrTMS (long-term: MD −4.83, 95% CrI −6.42 to −3.26) were effective in improving UPDRS-III score compared with Sham stimulation. For TUGT time, HFrTMS (short-term: MD −2.04, 95% CrI −3.26 to −0.8, long-term: MD −2.66, 95% CrI −3.55 to −1.77), and AtDCS (short-term: MD −0.8, 95% CrI −1.26 to −0.34, long-term: MD −0.69, 95% CrI −1.31 to −0.08) produced a significant difference compared to Sham stimulation. However, no statistical difference was found in FOGQ score among the various groups. According to the surface under curve ranking area, HFrTMS ranked first in short-term UPDRS-III score (0.77), short-term (0.82), and long-term (0.84) TUGT time, and short-term FOGQ score (0.73). With respect to the safety outcomes, all strategies indicated few and self-limiting AEs.

**Conclusion:**

HFrTMS may be the optimal non-invasive brain stimulation (NIBS) intervention to improve motor function in patients with PD while NIBS has generally been well tolerated. However, further studies focusing on the clinical outcomes resulting from the different combined schedules of tDCS and rTMS are required.

**Systematic review registration:**

https://inplasy.com/inplasy-2023-4-0087/, identifier: 202340087.

## Introduction

Parkinson's disease (PD) is the second leading neurodegenerative disease globally characterized by progressive motor and non-motor symptoms (Dorsey et al., 2018; Aarsland et al., 2021). The number of diagnosed cases is predicted to exceed 12 million by 2040, making PD the leading cause of neurological disability (Dorsey et al., 2018; Aarsland et al., 2021). With the progress of the disease, PD patients may experience disordered walking and balance accompanied by social isolation and decreased quality of life (Tomlinson et al., 2013; Bayle et al., 2016). Although drugs such as dopaminergic can relieve motor symptoms in some cases, the degeneration of non-dopaminergic neurons may result in symptoms refractory to substitution of dopamine (Benninger et al., 2011). Deep brain stimulation is invasive and associated with risks, such as intraoperative bleeding and infections. Gutchess et al. concluded that the therapeutic schedule of neurodegenerative disorders should focus on the progress of degeneration or brain plasticity (Gutchess, 2014). Brain plasticity, defined as the ability of neural system to organize structural or functional connectivity to adapt to internal or external environmental changes, is crucial in the learning and memory processes (Stengel et al., 2022). Previous studies demonstrated that non-invasive brain stimulation (NIBS) could enable plasticity reorganization processes which was achieved by reconstruction of neural network, indicating that NIBS might be a promising treatment for degenerative neurological disorders (Kim et al., 2019; Li et al., 2021). The rationale for the application of NIBS is that when specific change of brain network activities induces behavioral changes, it could normalize the activities and lead to improved behavior through direct effect on specific cortex or indirect effect on modulating remote cortical and subcortical regions that are connected to it (Romero Lauro et al., 2014; Sale et al., 2015). Moreover, NIBS has been used to improve abnormal brain functions in various diseases and disorders (including treatment-resistant depression and chronic pain; Concerto et al., 2015), and for rehabilitation after stroke (Bucur and Papagno, 2019). NIBS could also get access to cortex, activating cortico-basal ganglia-thalamocortical circuit that is associated with the pathophysiology of PD (Benninger and Hallett, 2015). Therefore, researchers are increasingly focusing on the specific efficacy and optimal modality of NIBS as an alternative therapy for motor symptoms in PD (Koch, 2013).

Transcranial direct current stimulation (tDCS) and repetitive transcranial magnetic stimulation (rTMS) are the two most common types of NIBS (Polanía et al., 2018). The rTMS is a non-invasive, well-tolerated technology of stimulation that delivers repeated magnetic pulses to specific brain regions through stimulation coils placed on the scalp, depolarizes nerve cells and generates activities of synaptic terminals, thus regulating the physiological functions of the brain (Pell et al., 2011). According to the pattern and frequency of stimulation, it could modulate cortical excitability that is associated with the clinical improvement of PD by promoting or inhibiting cortico-cortical synaptic connections (Chen and Chen, 2019). Specifically, different frequency of rTMS could regulate cortical excitability and constrain the influence in resting-state network without spilling to other networks (stimulation in primary motor cortex constrained in sensorimotor network; Cardenas-Morales et al., 2011; Stagg et al., 2014; Warren et al., 2014). Of these, High-frequency rTMS (≥5 Hz) enhanced cortical excitability, whereas low-frequency rTMS (≤ 1 Hz) inhibited cortical excitability for a short time (George and Aston-Jones, 2010). The tDCS is delivered using two conductive-rubber electrodes placed in a saline-soaked sponge to connect the DC stimulator (Lang et al., 2005) that alters cortical excitability through applying a weak current to the scalp to modulate the neuronal resting membrane potential (Nitsche and Paulus, 2000). Stimulation through the anode enhances cortical excitability, while stimulation through the cathode inhibits cortical excitability (Stagg and Nitsche, 2011). Furthermore, it has been proven that local tDCS could modulate resting-state networks, and excitatory in primary motor cortex could enhance the functional connectivity in motor network (Stagg et al., 2014). In addition, studies have reported that activation of the cortex through tDCS could induce secretion of dopamine, thus improving clinical symptoms of PD (Nitsche and Paulus, 2000; Fregni et al., 2006). Although the mechanism of action of NIBS technology remains elusive, both techniques appear to induce long-term enhancement and de-enhancement-like phenomena through multiple molecular and cellular mechanisms and demonstrate promising efficacy in alleviating the motor symptoms of PD (Polanía et al., 2018). Both types of NIBS have been confirmed to be safe, and the adverse effects after NIBS are mild and resolve quickly (Vonloh et al., 2013; Matsumoto and Ugawa, 2017). The rTMS has a broader range of clinical applications (including depression) (Rossi et al., 2009; Burke et al., 2019), whereas tDCS is less expensive and easier to learn and popularize, making home-use possible (Woods et al., 2016).

Considering the pathophysiological complexity of PD and participation of multiple brain regions in motor improvement, the application of NIBS might be different (de Oliveira et al., 2021). Although the effects of it on motor symptoms of PD have been confirmed in a previous meta-analysis (Zhang et al., 2022), the efficacy of rTMS and tDCS in specific frequencies and locations has not been compared. Thus, we performed a network meta-analysis (NMA) to comprehensively assess the effectiveness and safety of different rTMS and tDCS modes to treat the disordered motor function observed in PD. The result of our NMA may provide evidence-based recommendations for clinical decision-making.

## Methods

### Methods and materials

Our NMA complies with the Cochrane Handbook for systematic Reviews of Interventions Version 6.3 (Liberati et al., 2009) and the PRISMA checklist (Page et al., 2021). The meta-analysis has been registered and is available on INPLASY (registration ID: 202340087).

### Literature search

To perform the NMA, two reviewers (YJQ and ZQY) systemically searched PubMed, Embase, and the Cochrane Library databases, and collected eligible studies published from January 1, 2013 to January 1, 2023. The database was searched based on the combination of medical MeSH terms and general terms. We also reviewed meta-analyses, reviews, and the references of the included studies to supplement the search. The detailed search strategy and results can be found in Supplementary material (Supplementary Table 1).

### Eligibility criteria

Studies meeting the following criteria are enrolled: (1) participants: patients with a diagnosis of idiopathic PD, (2) intervention: patients received interventional NIBS, such as rTMS and tDCS, (3) comparison: patients received Sham stimulation, (4) outcomes: efficacy outcomes were pre–post changes in the Unified Parkinson's Disease Rating Scale part III (UPDRS-III) score, timed up and go (TUGT) time, and Freezing of Gait Questionnaire (FOGQ) score. UPDRS-III is the considered as the primary outcome, while TUGT time and FOGQ score are secondary outcomes. Additionally, we divided these scales into short-term and long-term efficacy. Short-term efficacy was defined as a change in the scales measured immediately at the end of NIBS treatment and up to 1 week later, whereas long-term efficacy was defined as a change in the scales at the 2-week follow-up and beyond. Safety outcomes were indicative of adverse events (AEs) after stimulation, (5) study type: studies using a crossover design or randomized control trial (RCT) design. Studies were excluded if they fulfilled at least one of the following criteria: (1) conference abstract, editorial, review, case report, single-arm clinical trial, (2) studies not written in English, (3) study with unavailable data, (4) studies that did not include any of the outcome measures.

### Study selection and data collection

Two independent reviewers (YJQ and ZQY) screened the research data and information, and discrepancies were resolved by discussing with the third author (MW), who did not perform data collection. The following variables were extracted: first author in brief, intervention, position site of NIBS, sex, age, baseline Hoehn–Yahr scale, and outcome Studies matching at least one of the following were excluded measures. When data extracted from an included manuscript described continuous variables as medians and interquartile ranges rather than means and standard deviations, we transformed these data according to the method described by Hozo et al. (2005). When there was missing data, we contacted the author to obtain complete data. When we did not receive a response, we removed the study with missing data.

### Quality and risk of bias assessments

The quality of evidence assessment of each paired comparison was estimated by the Grading of Recommendations Assessment, Development, and Evaluations w Studies matching at least one of the following were excluded working group approach using the confidence in NMA framework (Nikolakopoulou et al., 2020). Each study started with a relatively high point for estimation and would be downgraded considering the limitations of risk of bias, publication bias, inconsistency (heterogeneity), and imprecision (Guyatt et al., 2008). The risk of bias of each included study was evaluated using the Cochrane Collaboration tool (Higgins et al., 2011). Two reviewers classified studies according to the risk of bias (low, high, or unclear) using Review Manager 5.4. Any disagreements were resolved through discussion.

### Statistical analysis

Prior to NMA, we performed a pairwise meta-analysis of direct evidence using Review Manager 5.4. The risk ratio (OR) and MDs with 95% confidence intervals (95% CIs) were used for dichotomous and continuous variables. Statistical heterogeneity between trials was evaluated with the *I*^2^ statistics. *I*^2^ < 30%, 30 to 50%, and >50% were recognized as low, moderate, and high heterogeneity, respectively. We selected the random effect model for analysis when heterogeneity was >50%, otherwise, we selected the fixed effect model (Higgins et al., 2003; Tufanaru et al., 2015).

This NMA was conducted based on a Bayesian framework using R software (version 4.2.2), using the “gemtc” package (van Valkenhoef et al., 2012). Dichotomous outcomes were analyzed using log response ratios with 95% credible intervals (CrIs), and continuous variables were analyzed with mean differences (MDs) with 95% CrIs instead of standard mean difference because the rating scale used uniform units. The NMA plot was conducted using Stata 17.0 and each node indicates a type of intervention. The size of the node and the thickness of the edge represent the number of participants, and the number of trials comparing the two interventions, respectively. Node splitting models were constructed to test the consistency and stability of the network structure (Higgins et al., 2012; Krahn et al., 2014). The convergence of the model was evaluated with the track and density plot and the Brooks–Gelman–Rubin diagnosis plot. Additionally, to rank the efficacy of different NIBS interventions, we generated the surface under the cumulative ranking (SUCRA) plot with a percentage ranging from 0 to 1. A treatment showing a higher SUCRA value indicated a greater probability of being more effective than other treatments. Moreover, *I*^2^ statistics were used to assess heterogeneity in the NMA. A sensitivity analysis was then performed by excluding studies with high risk of bias. For all analyses, *P*-values were calculated as two-sided and a cut-off point of 0.05 was considered statistically significant. In addition, a funnel plot was generated to assess possible publication bias using STATA 17.0 (Chaimani et al., 2013), and an asymmetric distribution of the funnel plot indicated obvious publication bias.

## Results

### Study characteristics

**Figure 1:**
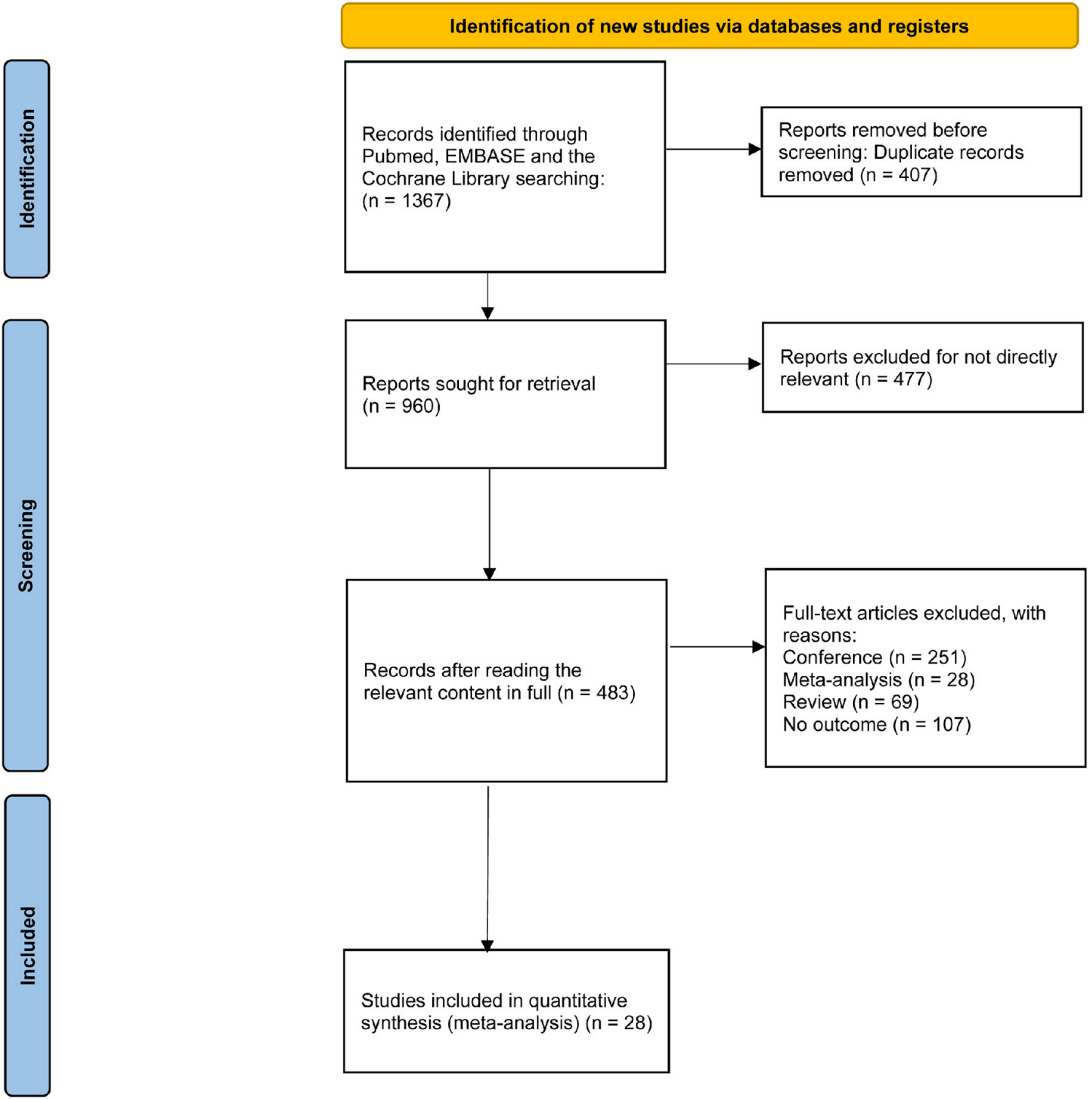
The study search, selection, and inclusion process.

The study selection process is shown in Figure 1. We identified 1,367 records from three electronic databases. After removing 407 duplicates, 960 records were screened by title and abstract. Of these, 477 records were excluded because they were irrelevant to the topic. We then assessed the eligibility of 483 full articles and excluded 455 studies with different study types. Overall, 28 studies that fulfilled the selection criteria were retained for further analysis. The baseline characteristics of the enrolled manuscripts are illustrated in Table 1. Of these, 17 studies are related to rTMS, including 15 studies on HFrTMS, three studies on LFrTMS, and one study on the combination of HFrTMS and LFrTMS. Eleven studies are related to tDCS, including 10 studies on AtDCS and one study on the combination of AtDCS and CtDCS. In addition, one study applied a combination of HFrTMS and AtDCS.

**Table 1:** Characteristics of the included randomized controlled trials.

**References**	**Intervention**	**Site**	**Male**	**Age (Mean ±SD)**	**H-Y (Mean ±SD)**	**Outcome**
**rTMS**						
Yang et al. (2013)	HFrTMS	M1	5	65.20 ± 11.08	2.30 ± 0.42	TUGT
	Sham		7	67.00 ± 13.21	2.35 ± 0.41	
Maruo et al. (2013)	HFrTMS	M1	11	63.00 ± 11.30	3.10 ± 0.5	UPDRS-III
	Sham		11	63.00 ± 11.30	3.10 ± 0.50	
Kim et al. (2015)	HFrTMS	M1	NR	64.50 ± 8.40	3.00 ± 0.5	UPDRS-III, TUGT, FOGQ
	Sham		NR	64.50 ± 8.40	3.00 ± 0.50	
Chang et al. (2016)	HFrTMS	M1	6	71.90 ± 7.80	NR	UPDRS-III, TUGT, FOGQ
	Sham		6	71.90 ± 7.80	NR	
Makkos et al. (2016)	HFrTMS	M1	13	66.64 ± 10.27	2.38 ± 0.84	UPDRS-III, TUGT
	Sham		11	66.00 ± 6.36	2.26 ± 0.74	
Brys et al. (2016)	HFrTMS	M1	11	59.60 ± 12.60	2.29 ± 0.31	UPDRS-III
	HFrTMS	DLPFC	9	64.60 ± 12.30	2.83 ± 0.77	
	HFrTMS	M1_DLPFC	6	64.90 ± 8.00	2.48 ± 0.51	
	Sham		11	64.00 ± 7.40	2.33 ± 0.35	
Chang et al. (2017)	HFrTMS_AtDCS	M1(rTMS)_DLPFC(tDCS)	9	63.60 ± 7.50	2.53 ± 0.54	UPDRS-III, TUGT, FOGQ
	HFrTMS		11	63.80 ± 8.30	2.41 ± 0.44	
Yokoe et al. (2018)	HFrTMS	M1	7	69.10 ± 8.40	3.50 ± 0.60	UPDRS-III
	HFrTMS	SMA	7	69.10 ± 8.40	3.50 ± 0.60	
	HFrTMS	DLPFC	7	69.10 ± 8.40	3.50 ± 0.60	
	Sham		7	69.10 ± 8.40	3.50 ± 0.60	
Cohen et al. (2018)	HFrTMS_LFrTMS	M1_DLPFC	17	64.40 ± 6.80	2.18 ± 0.40	UPDRS-III, TUGT
	Sham		15	66.80 ± 8.10	2.18 ± 0.40	
Aftanas et al. (2018)	HFrTMS	M1_DLPFC	13	63.20 ± 8.33	NR	UPDRS-III
	Sham		10	63.80 ± 7.50	NR	
Mi et al. (2019)	HFrTMS	SMA	9	62.65 ± 10.56	2.60 ± 0.8	UPDRS-III, TUGT, FOGQ
	Sham		5	65.60 ± 8.68	2.35 ± 0.91	
Khedr et al. (2019)	HFrTMS	M1	NR	59.58 ± 11.28	NR	UPDRS-III
	LFrTMS		NR	55.88 ± 13.84	NR	
Khedr et al. (2019)	HFrTMS	M1	NR	60.70 ± 8.80	3.10 ± 1.10	UPDRS-III
	Sham		NR	57.40 ± 10.00	3.50 ± 1.00	
Chung et al. (2020)	HFrTMS	M1	10	62.70 ± 6.80	2.20 ± 0.3	UPDRS-III, TUGT
	LFrTMS		9	62.10 ± 5.70	2.20 ± 0.40	
	Sham		7	62.10 ± 5.70	2.30 ± 0.30	
Li et al. (2020)	HFrTMS	M1	8	61.67 ± 6.92	1.85 ± 0.63	UPDRS-III
	Sham		8	61.46 ± 8.40	1.83 ± 0.64	
Zhuang et al. (2020)	LFrTMS	DLPFC	11	60.58 ± 9.21	2.00 ± 0.80	UPDRS-III
	Sham		7	61.57 ± 13.25	2.34 ± 1.03	
Aftanas et al. (2022)	HFrTMS	M1_DLPFC	12	63.48 ± 8.26	2.55 ± 0.50	UPDRS-III
	Sham		10	63.86 ± 7.79	2.56 ± 0.50	
**tDCS**						
Manor et al. (2021)	AtDCS	M1_DLPFC	31	71.00 ± 8.00	NR	UPDRS-III, TUGT, FOGQ
	Sham		28	69.00 ± 7.00	NR	
Na et al. (2022)	AtDCS	M1	3	63.73 ± 6.57	1.74 ± 1.70	UPDRS-III, TUGT, FOGQ
	Sham		7	65.08 ± 6.46	2.00 ± 1.68	
Lee and Kim (2021)	AtDCS	SMA	6	70.00 ± 3.76	2.47 ±0.52	FOGQ
	Sham		8	71.33 ± 3.27	2.80 ±0.41	
Kaski et al. (2014)	AtDCS	M1	NR	NR	NR	TUGT
	Sham		NR	NR	NR	
Manenti et al. (2016)	AtDCS	DLPFC	4	69.00 ± 9.10	2.20 ± 0.60	UPDRS-III, TUGT
	Sham		7	69.10 ± 5.60	2.30 ± 0.40	
Costa-Ribeiro et al. (2017)	AtDCS	M1	8	61.10 ± 9.10	2.36 ± 0.68	UPDRS-III, TUGT
	Sham		7	62.00 ± 16.70	2.32 ± 0.65	
Wong et al. (2022)	AtDCS	M1	8	54.20 ± 4.10	1.89 ± 0	TUGT
	AtDCS	DLPFC	6	50.09 ± 2.40	1.67 ± 0.50	
	AtDCS	Cerebellum	2	61.30 ± 7.90	2.13 ± 0.60	
	Sham		3	58.30 ± 8.00	1.78 ± 0.70	
Biundo et al. (2015)	AtDCS	DLPFC	NR	69.10 ± 7.60	NR	UPDRS-III
	Sham		NR	72.30 ± 4.10	NR	
Yotnuengnit et al. (2018)	AtDCS	M1	21	66.30 ± 8.95	NR	UPDRS-III
	Sham		12	62.70 ± 8.80	NR	
De Icco et al. (2022)	AtDCS_CtDCS	M1	9	71.90 ± 5.20	2.62 ± 0.84	UPDRS-III
	Sham		12	73.70 ± 5.00	2.53 ± 0.72	
Ferrucci et al. (2016)	AtDCS	M1	5	74.33 ± 7.53	2.50 ± 0.33	UPDRS-III
	AtDCS	Cerebellum	5	74.33 ± 7.53	2.50 ± 0.33	
	Sham		5	74.33 ± 7.53	2.50 ± 0.33	

HFrTMS, high-frequency repetitive transcranial magnetic stimulation; LFrTMS, low-frequency repetitive transcranial magnetic stimulation; AtDCS, anodal transcranial direct current stimulation; CtDCS, cathode transcranial direct current stimulation; MC, motor cortex; M1, primary motor cortex; DLPFC, dorsolateral prefrontal cortex; SMA, supplementary motor area; SD, standard difference; NR, not reported; H-Y, Hoehn and Yahr; UPDRS-III, Unified Parkinson's Disease Rating Scale part III; TUGT, Time Up and Go Test; FOGQ, Freezing of Gait Questionnaire.

### NMA

**Figure 2:**
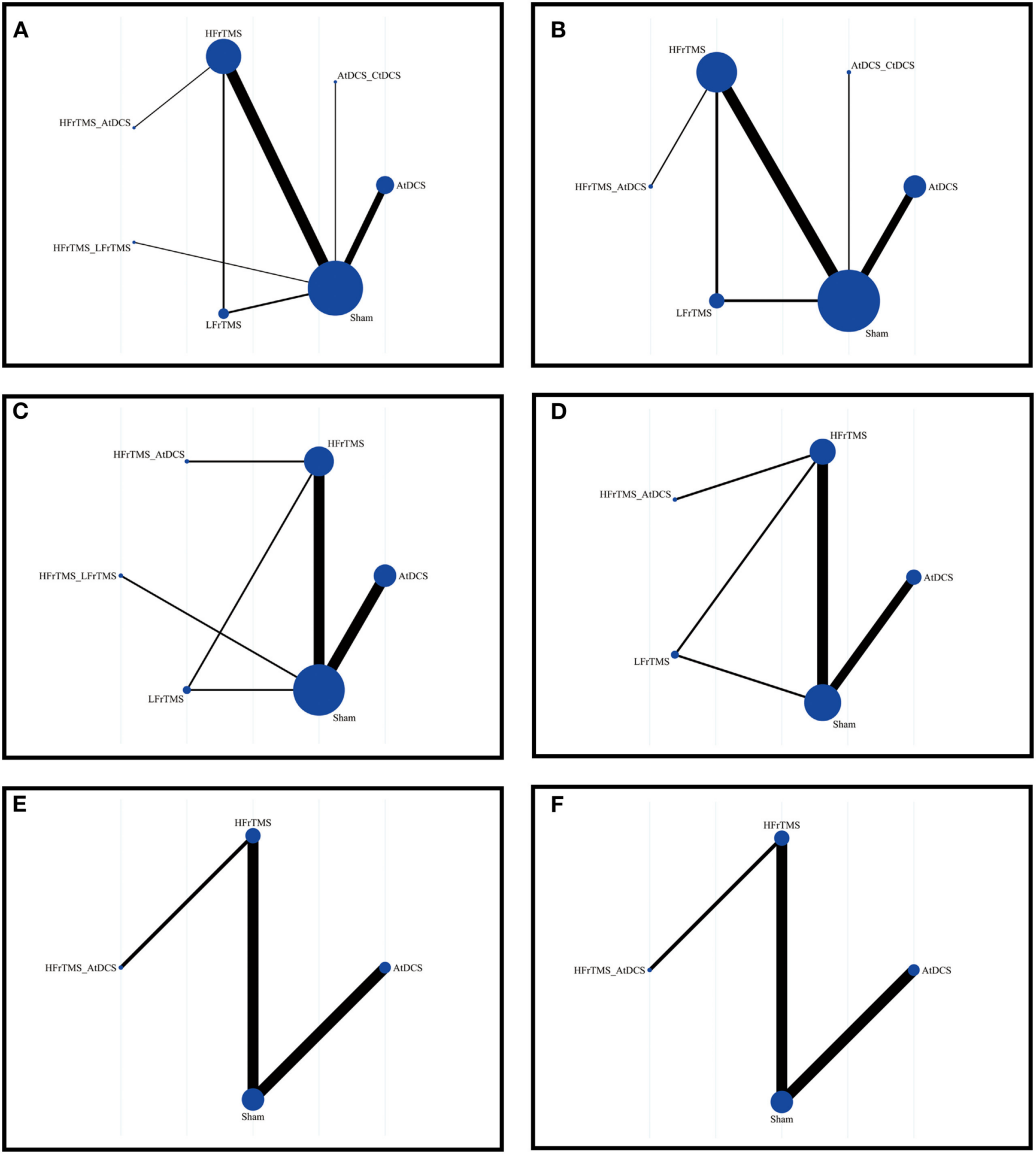
Network of trails comparing non-invasive brain stimulation of Parkinson's disease. The size of circles represented the number of participants for each intervention and the width of lines represented the number of trials compared between treatments. **(A)** Short-term UPDRS-III score. **(B)** Long-term UPDRS-III score. **(C)** Short-term TUGT time. **(D)** Long-term TUGT time. **(E)** Short-time FOGQ score. **(F)** Short-time FOGQ score. UPDRS-III, Unified Parkinson's Disease Rating Scale part III; TUGT, Time Up and Go Test; FOGQ, Freezing of Gait Questionnaire.

**Figure 3:**
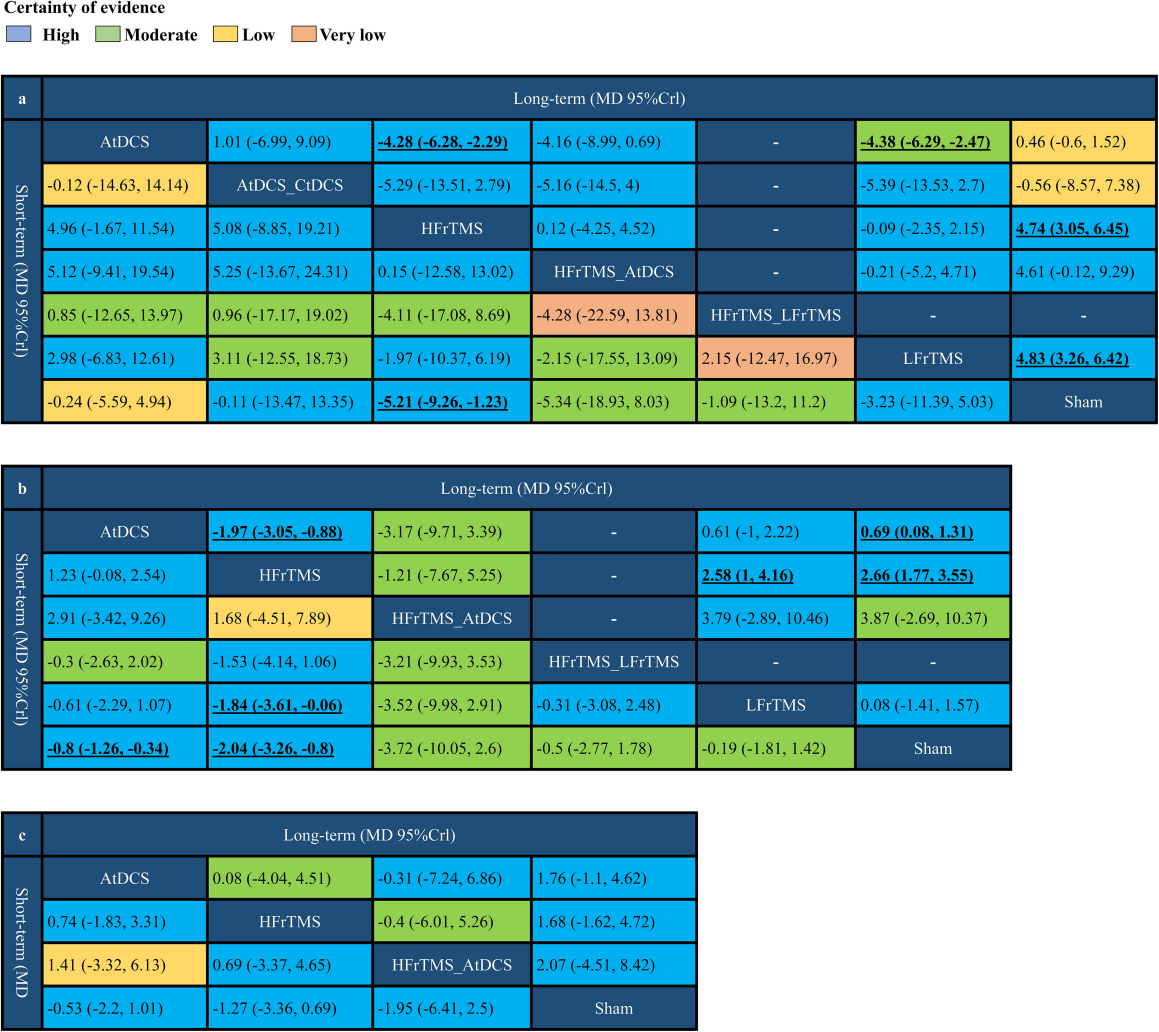
Network meta-analysis results comparing non-invasive brain stimulation of Parkinson's disease. **(a)** Short-term and long-term UPDRS-III score. **(b)** Short-term and long-term TUGT time. **(c)** Short-term and long-term FOGQ score. Values in bold indicate significant difference. UPDRS-III, Unified Parkinson's Disease Rating Scale part III; TUGT, Time Up and Go Test; FOGQ, Freezing of Gait Questionnaire.

Figure 2 and Supplementary Figures 1–6 showed the network comparisons of different strategies of NIBS on changes of scales. In short-term outcomes, HFrTMS was correlated with a significant improvement in UPDRS-III compared to those resulting from Sham stimulation (MD 5.21, 95% CrI 1.23 to 9.26; Figure 3a). For TUGT time, HFrTMS also resulted in a greater improvement than did LFrTMS (MD 1.84, 95% CrI 0.06 to 3.61) and Sham stimulation (MD 2.04, 95% Crl 0.8 to 3.26). Moreover, AtDCS was superior to Sham stimulation in effects on TUGT time (MD 0.8, 95% CrI 0.34 to 1.26; Figure 3b). However, there were no statistical differences in FOGQ score when any of different strategies of NIBS were used (Figure 3c).

For the long-term efficacy, HFrTMS (MD 4.28, 95% CrI 2.29 to 6.28) and LFrTMS (MD 4.38, 95% CrI 2.47 to 6.29) were associated with greater improvement in UPDRS-III score compared to those resulting from AtDCS. In addition, HFrTMS (MD 4.74, 95% CrI 3.05 to 6.45) and LFrTMS (MD 4.83, 95% CrI 3.26 to 6.42) were associated with greater improvement when compared to Sham stimulation (Figure 3a). HFrTMS resulted in statistical differences in TUGT time compared to those resulting from AtDCS (MD 1.97, 95% CrI 0.88 to 3.05), LFrTMS (MD 2.58, 95% CrI 1.00 to 4.16), or Sham stimulation (MD 2.66, 95% CrI 1.77 to 3.55). Statistical significance was also observed between the effects of AtDCS and Sham stimulation on TUGT time (MD 0.69, 95% Crl 0.08 to 1.31; Figure 3b). Similar to the short-term findings, no statistical difference was observed in long-term improvement in FOGQ score among the various groups (Figure 3c).

**Figure 4:**
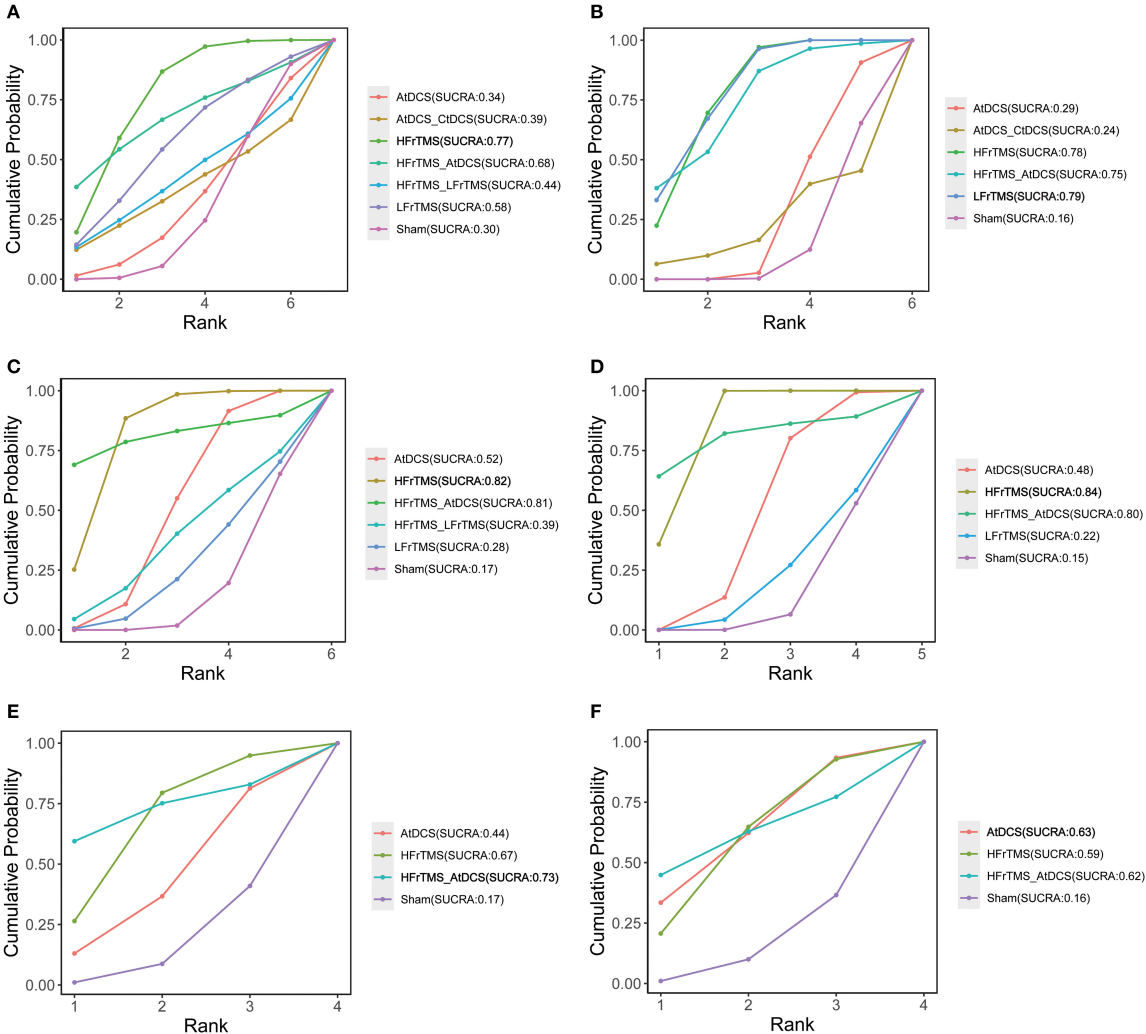
Cumulative probability of each intervention with specific rank. A higher SUCRA value indicated a better rank for the intervention. **(A)** Short-term UPDRS-III score. **(B)** Long-term UPDRS-III score. **(C)** Short-term TUGT time. **(D)** Long-term TUGT time. **(E)** Short-time FOGQ score. **(F)** Short-time FOGQ score. Values in bold indicate the first ranking. SUCRA, surface under curve ranking area; UPDRS-III, Unified Parkinson's Disease Rating Scale part III; TUGT, Time Up and Go Test; FOGQ, Freezing of Gait Questionnaire.

The ranking probability of each intervention for efficacy outcomes is shown in Figure 4. In short-term efficacy, HFrTMS (SUCRA, 0.77) ranked first in the UPDRS-III score and TUGT time (Figures 4A, C). For the FOGQ score. HFrTMS_AtDCS (SUCRA, 0.73) had the highest-ranked probability (Figure 4E). In long-term efficacy, LFrTMS (SUCRA, 0.79) ranked first in the UPDRS-III score (Figure 4B), whereas HFrTMS (SUCRA,0.84) resulted in the highest SUCRA value in TUGT time (Figure 4D). AtDCS (SUCRA,0.63) ranked the highest in FOGQ score (Figure 4F).

### Pairwise meta-analysis

The detailed results of pairwise meta-analysis are shown in Supplementary Figures 27–54. We classified different strategies according to the site the NIBS were placed. The position of the NIBS was primarily classified into primary motor cortex (M1), motor cortex, premotor cortex, supplementary motor area (SMA), and dorsolateral prefrontal cortex (DLPFC). In terms of short-term efficacy, HFrTMS placed over the M1_DLPFC resulted in a significant improvement in UPDRS-III score compared to those resulting from Sham stimulation (MD −14.48, 95% CI −16.06 to −12.93, *I*^2^ 0, *P* < 0.00001, High). In TUGT time, HFrTMS over the SMA site was correlated with a more significant improvement compared to Sham stimulation (MD −3.33, 95% CI −5.45 to −1.21, *I*^2^ NA, *P* < 0.002, Moderate). Furthermore, a significant difference was observed in TUGT time resulting from AtDCS placed over the M1_DLPFC site (MD −1.00, 95% CI −1.53 to −0.47, *I*^2^ NA, *P* < 0.0002, Moderate) and those resulting from Sham stimulation (Table 2).

**Table 2:** Summary and detailed effects sizes of NIBS in different site as compared with the sham from pair-wise meta-analysis of short-term outcomes.

**Short-term outcomes or subgroup title (total and by drug)**	**No. of trials contributing to the meta-analysis**	**No. of participants contributing to the meta-analysis**	**MD [95% CI]**	**P-value**	***I*^2^ (%)**	**GRADE**
**UPDRS-III**						
MC	2	82	−0.53 [−1.37, 0.31]	0.21	0	High
AtDCS	2	82	−0.53 [−1.37, 0.31]	0.21	0	High
M1	11	366	−1.33 [−4.32, 1.67]	0.39	89	High
HFrTMS	8	285	−1.28 [−4.96, 2.41]	0.50	94	High
LFrTMS	1	33	−3.10 [−9.49, 3.29]	0.34	NA	Low
AtDCS	2	41	−0.74 [−4.84, 3.36]	0.72	0	Moderate
AtDCS_CtDCS	1	28	−0.08 [−6.07, 5.91]	0.98	NA	Low
SMA	2	68	−0.99 [−7.38, 5.41]	0.76	93	Moderate
HFrTMS	2	68	−0.99 [−7.38, 5.41]	0.76	93	Moderate
DLPFC	4	115	1.37 [0.18, 2.56]	0.02	35	Very low
HFrTMS	1	38	1.60 [0.38, 2.82]	0.01	NA	Very low
LFrTMS	1	33	−5.53 [−13.29, 2.23]	0.16	NA	Low
AtDCS	2	44	−1.74 [−10.40, 6.92]	0.69	0	Low
M1_DLPFC	4	209	−6.76 [−15.20, 1.67]	0.12	99	Moderate
HFrTMS	2	96	−14.48 [−16.04, −12.93]	< 0.00001	0	High
HFrTMS_LFrTMS	1	42	−1.08 [−2.39, 0.23]	0.11	NA	Low
AtDCS	1	71	2.83 [0.62, 5.04]	0.01	NA	Very low
Cerebellum	1	18	−1.10 [−5.54, 3.34]	0.63	NA	Moderate
AtDCS	1	18	−1.10 [−5.54, 3.34]	0.63	NA	Moderate
**TUG**						
MC	1	22	0.78 [−1.92, 3.48]	0.57	NA	Moderate
AtDCS	1	22	0.78 [−1.92, 3.48]	0.57	NA	Moderate
M1	8	253	−0.60 [−1.42, 0.22]	0.15	0	High
HFrTMS	5	147	−1.35 [−2.88, 0.18]	0.08	0	High
LFrTMS	1	33	0.10 [−1.59, 1.79]	0.91	NA	Moderate
AtDCS	3	73	−0.49 [−1.67, 0.69]	0.41	0	High
SMA	1	30	−3.33 [−5.45, −1.21]	0.002	NA	Moderate
HFrTMS	1	30	−3.33 [−5.45, −1.21]	0.002	NA	Moderate
DLPFC	2	38	−0.17 [−1.57, 1.23]	0.81	0	High
AtDCS	2	38	−0.17 [−1.57, 1.23]	0.81	0	High
M1_DLPFC	2	113	−0.97 [−1.49, −0.45]	0.0002	0	High
HFrTMS_LFrTMS	1	42	−0.50 [−2.77, 1.77]	0.67	NA	Moderate
AtDCS	1	71	−1.00 [−1.53, −0.47]	0.0002	NA	Moderate
Cerebellum	1	18	−0.65 [−3.61, 2.31]	0.67	NA	Moderate
AtDCS	1	18	−0.65 [−3.61, 2.31]	0.67	NA	Moderate
**FOG**						
M1	3	73	0.38 [−0.49, 1.26]	0.39	20	High
HFrTMS	2	50	−1.84 [−4.73, 1.05]	0.21	0	High
AtDCS	1	23	0.61 [−0.31, 1.53]	0.19	NA	Moderate
SMA	2	60	−1.02 [−2.11, 0.07]	0.07	0	High
HFrTMS	1	30	−1.00 [−2.20, 0.20]	0.10	NA	Moderate
AtDCS	1	30	−1.13 [−3.80, 1.54]	0.41	NA	Moderate
M1_DLPFC	1	71	−1.00 [−3.02, 1.02]	0.33	NA	Moderate
AtDCS	1	71	−1.00 [−3.02, 1.02]	0.33	NA	Moderate

HFrTMS, high-frequency repetitive transcranial magnetic stimulation; LFrTMS, low-frequency repetitive transcranial magnetic stimulation; AtDCS, anodal transcranial direct current stimulation; CtDCS, cathode transcranial direct current stimulation; MC, motor cortex; M1, primary motor cortex; DLPFC, dorsolateral prefrontal cortex; SMA, supplementary motor area; M, Mean Difference; CI, Confidence Interval; NA, Not applicable; UPDRS-III, Unified Parkinson's Disease Rating Scale part III; TUGT, Time Up and Go Test; FOGQ, Freezing of Gait Questionnaire.

Regarding long-term efficacy, both HFrTMS over the M1 site (MD −4.68, 95% CI −7.01 to −2.34, *I*^2^ 0, *P* < 0.0001, High) and the SMA site (MD −5.55, 95% CI −8.59 to −2.51, *I*^2^ N/A, *P* < 0.0003, Moderate) were associated with a significant reduction in UPDRS-III score when compared to Sham stimulation. Moreover, LFrTMS applied over the DLPFC site (MD −5.22, 95% CI −6.89 to −3.55, *I*^2^ N/A, *P* < 0.00001, Moderate) demonstrated a greater reduction in UPDRS-III than did Sham stimulation. Additionally, HFrTMS over the M1 (MD −2.25, 95% CI −3.23 to −1.27, *I*^2^ 0, *P* < 0.00001, High) and the SMA (MD −4.61, 95% CI −6.74 to −2.48, *I*^2^ N/A, *P* < 0.00001, Moderate) sites was associated with a greater reduction in TUGT time than that of Sham stimulation. AtDCS placed over the M1 site (MD −1.16, 95% CI −2.30 to −0.02, *I*^2^ N/A, *P* < 0.05, Moderate) also resulted in significant reduction in TUGT time when compared to the time associated with Sham stimulation. In long-term FOGQ score, HFrTMS over the SMA site showed greater improvement than did Sham stimulation (MD −2.53, 95% CI −3.73 to −1.33, *I*^2^ N/A, *P* < 0.0001, Moderate; Table 3).

**Table 3:** Summary and detailed effects sizes of NIBS in different site as compared with the sham from pair-wise meta-analysis of long-term outcomes.

**Long-term outcomes or subgroup title (total and by drug)**	**No. of trials contributing to the meta-analysis**	**No. of participants contributing to the meta-analysis**	**MD [95% CI]**	**P-value**	***I*^2^ (%)**	**GRADE**
**UPDRS-III**						
MC	2	75	−0.56 [−1.79, 0.66]	0.37	0	High
AtDCS	2	75	−0.56 [−1.79, 0.66]	0.37	0	High
M1	9	318	−4.19 [−6.16, −2.22]	< 0.0001	0	High
HFrTMS	7	234	−4.68 [−7.01, −2.34]	< 0.0001	0	High
LFrTMS	1	33	−2.80 [−9.34, 3.74]	0.40	NA	Low
AtDCS	1	23	−4.66 [−9.97, 0.65]	0.09	NA	Low
AtDCS_CtDCS	1	28	0.55 [−7.48, 8.58]	0.89	NA	Low
SMA	1	30	−5.55 [−8.59, −2.51]	0.0003	NA	Moderate
HFrTMS	1	30	−5.55 [−8.59, −2.51]	0.0003	NA	Moderate
DLPFC	4	104	−5.00 [−6.54, −3.46]	< 0.00001	48	Moderate
HFrTMS	1	27	−3.17 [−8.04, 1.70]	0.20	NA	Moderate
LFrTMS	1	33	−5.22 [−6.89, −3.55]	< 0.00001	NA	Moderate
AtDCS	2	44	−4.96 [−12.07, 2.15]	0.17	81	Low
M1_DLPFC	2	93	0.60 [−1.47, 2.67]	0.57	20	High
HFrTMS	1	35	−1.37 [−5.38, 2.64]	0.50	NA	Moderate
AtDCS	1	58	1.31 [−1.10, 3.72]	0.29	NA	Moderate
**TUG**						
MC	1	22	−2.43 [−8.92, 4.06]	0.37	0	Low
AtDCS	1	22	−2.43 [−8.92, 4.06]	0.37	0	Low
M1	5	183	−1.43 [−2.10, −0.76]	< 0.0001	33	High
HFrTMS	4	127	−2.25 [−3.23, −1.27]	< 0.00001	0	High
LFrTMS	1	33	0.20 [−1.38, 1.78]	0.80	NA	Moderate
AtDCS	1	23	−1.16 [−2.30, −0.02]	0.05	NA	Moderate
SMA	1	30	−4.61 [−6.74, −2.48]	< 0.0001	NA	Moderate
HFrTMS	1	30	−4.61 [−6.74, −2.48]	< 0.0001	NA	Moderate
DLPFC	1	20	0.35 [−1.40, 2.10]	0.69	NA	Moderate
AtDCS	1	20	0.35 [−1.40, 2.10]	0.69	NA	Moderate
M1_DLPFC	1	58	−0.65 [−1.46, 0.16]	0.12	NA	Moderate
AtDCS	1	58	−0.65 [−1.46, 0.16]	0.12	NA	Moderate
**FOG**						
M1	3	73	−0.09 [−0.93, 0.75]	0.83	0	High
HFrTMS	2	50	−0.77 [−3.49, 1.95]	0.58	0	High
AtDCS	1	23	−0.02 [−0.91, 0.87]	0.96	NA	Moderate
SMA	2	60	−2.28 [−3.35, −1.21]	< 0.0001	0	High
HFrTMS	1	30	−2.53 [−3.73, −1.33]	< 0.0001	NA	Moderate
AtDCS	1	30	−1.27 [−3.66, 1.12]	0.30	NA	Moderate
M1_DLPFC	1	58	−1.00 [−3.47, 1.47]	0.43	NA	Moderate
AtDCS	1	58	−1.00 [−3.47, 1.47]	0.43	NA	Moderate

HFrTMS, high-frequency repetitive transcranial magnetic stimulation; LFrTMS, low-frequency repetitive transcranial magnetic stimulation; AtDCS, anodal transcranial direct current stimulation; CtDCS, cathode transcranial direct current stimulation; MC, motor cortex; M1, primary motor cortex; DLPFC, dorsolateral prefrontal cortex; SMA, supplementary motor area; M, Mean Difference; CI, Confidence Interval; NA, Not applicable; UPDRS-III, Unified Parkinson's Disease Rating Scale part III; TUGT, Time Up and Go Test; FOGQ, Freezing of Gait Questionnaire.

### Acceptability and AEs

Both rTMS and tDCS were safe and well-tolerated. Only eight RCTs in our NMA reported AEs, headache, neck pain, and burning sensation were the most common AEs. Seven of the studies (Kim et al., 2015; Brys et al., 2016; Chang et al., 2017; Cohen et al., 2018; Khedr et al., 2019; Li et al., 2020; Zhuang et al., 2020) reported headache or neck pain in several patients and their symptoms were relieved quickly. One RCT reported two patients experienced a burning sensation that was resolved independently (Yotnuengnit et al., 2018). Detailed descriptions of AEs are shown in Supplementary Table 2.

### Risk of bias, quality of evidence, and publication bias

**Figure 5:**
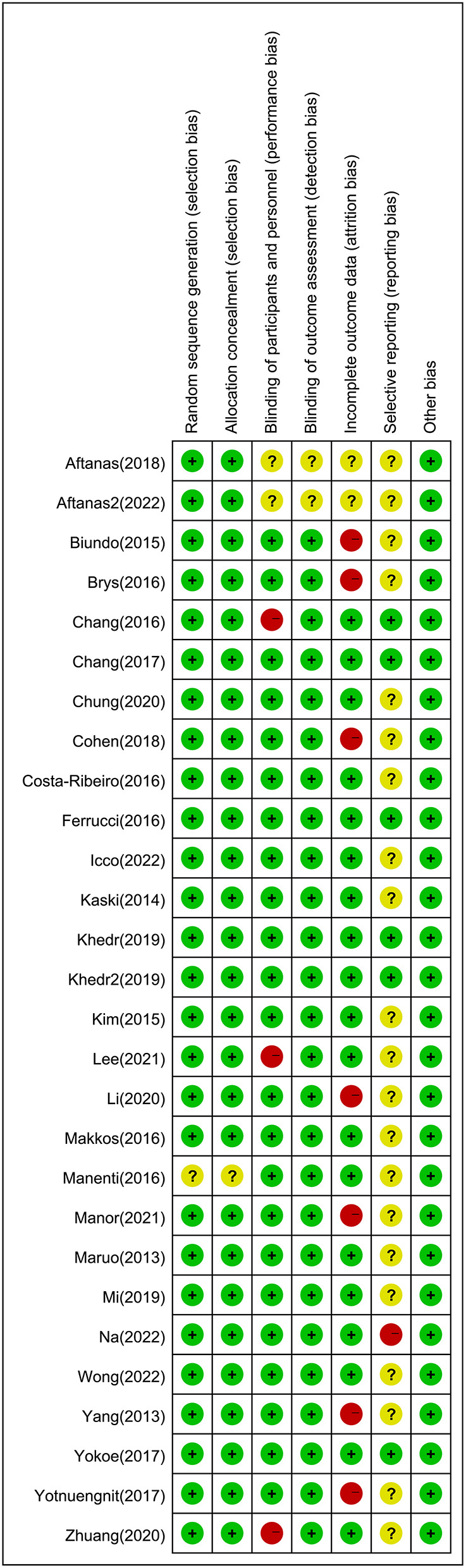
Risk of bias: a summary table for each risk of bias item for each study.

The risk of bias for the included studies is shown in Figure 5. The risk of bias in a random sequence generation was observed in one study to be unclear. The risk of bias in allocation concealment in one study was also unclear. The blinding of participants and personnel in three studies showed high risk of bias, and the risk of bias was unclear in two studies. With regard to blinding of outcome assessment, the risk of bias in the published studies was unclear. Seven studies had a high risk of bias in terms of incomplete outcome data, and the risk of bias was unclear in two studies. In selective reporting, there was a high risk of bias in one study and unclear bias risk in 21 studies. The GRADE assessment showed that the quality of evidence in most enrolled studies ranged from low to moderate, imprecision and heterogeneity were the main reasons for downgrading the certainty of evidence (Supplementary Table 4). The funnel plot was relatively symmetric for publication bias, indicating that no potential publication bias affected the NMA (Supplementary Figures 20–26; van Aert et al., 2019).

### Heterogeneity, sensitive analysis, and consistency analysis

The network *I*^2^-values for each outcome are shown in Supplementary Figures 7–12. The comparison of AtDCS and Sham stimulation demonstrated high heterogeneity in changes in long-term UPDRS-III (56.6%) and short-term (78.8%) and long-term FOGQ (92.5%), whereas the comparison between HFrTMS and Sham stimulation showed high heterogeneity in short-term UPDRS-III changes (98.7%). Sensitivity analysis was conducted by excluding four studies (Maruo et al., 2013; Aftanas et al., 2018, 2022; Yokoe et al., 2018) with high heterogeneity resulting from different stimulation sites, the overall heterogeneity of short-term UPDRS-III changes decreased to 22%, indicating the main heterogeneity was derived from the stimulation sites. The results of short-term UPDRS-III changes in studies with low heterogeneity are shown in Supplementary Table 3. In addition, because of a shortage of clinical data on TUGT and FOGQ score, we conducted only a node-split model on UPDRS-III score. As shown in Supplementary Figures 19, 20, no significant inconsistency was observed in network structure, indicating the robustness of the NMA. The convergence result is illustrated in Supplementary Figures 13–18, fluctuation was not recognized and the density graph was normally distributed, indicating an excellent convergence.

## Discussion

A lack of consensus with regards to optimal modalities of rTMS and tDCS still exists, despite considerable attention garnered for the use of NIBS for PD. To the best of our knowledge, this is the first NMA to compare different modalities of rTMS and tDCS in the improvement of motor symptoms of patients with PD. Our findings demonstrated that, whether a long-term or short-term outcome, HFrTMS was associated with significant improvements in UPDRS-III and TUGT time. In addition, HFrTMS was superior to LFrTMS in the improvement of TUGT time. Furthermore, the AtDCS group experienced less improvement in long-term UPDRS-III and TUGT time compared to the HFrTMS group, AtDCS was also inferior to LFrTMS in the improvement of long-term UPDRS-III score. In terms of incidence of AEs, all NIBS treatments resulted in few and mild AEs.

The specific neurophysiological mechanism underlying the beneficial effects of rTMS and tDCS on motor function remains unclear, it is possible that different pathophysiological mechanisms could indirectly influence our findings (Krogh et al., 2022). With regard to rTMS, when the brain is a conducting medium exposed to a magnetic field, it generates a current in the primary coil that is the source of the magnetic field. The neurons are then activated by the penetration of magnetic field into scalp and skull and the secondary current generated by the electric field (Rossi et al., 2009). Klomjai et al. proved that the effects of rTMS can exceed the duration of stimulation (Klomjai et al., 2015). Current evidence has demonstrated that the long-term intervention with rTMS can alter synaptic plasticity within the corticospinal tract by enhancing excitatory postsynaptic potentials, which might be associated with the availability and sensitivity of Ca^2+^, NO, and glutamate (Hoogendam et al., 2010; Klomjai et al., 2015). Although the simulation of tDCS is broad and non-focal, it could influence neuronal synchronization, connections, and oscillations (Brabenec et al., 2021). Fregni et al. suggested that tDCS may promote neural connectivity in cortical and subcortical networks (e.g., basal ganglia thalamocortical motor circuits) and may mitigate the effects of the basal ganglia degeneration that occurs in patients with PD (Fregni et al., 2006; Benninger et al., 2010; Ferrucci et al., 2016). Low-frequency magnetic stimulation has been shown to produce inhibitory effects on the motor cortex (Chen et al., 1997), whereas high-frequency magnetic stimulation produced immediate excitatory effects (Wu et al., 2000). With regard to direct current stimulation, anodal stimulation is classified as excitatory: it downgrades the threshold of neuronal activation and improves neural efficiency, mood, and cognition (Pellicciari and Miniussi, 2018). In contrast, cathodal stimulation leads to hyperpolarization of the resting membrane potential and decreased neuronal excitability (Brunoni et al., 2016).

The UPDRS-III is now widely used to evaluate motor function improvement in PD. According to our SUCRA ranking, HFrTMS had the best short-term efficacy for improving UPDRS-III score in PD. The potential explanation for the application of HFrTMS is that the under-activation of brain regions, such as M1, DLPFC, and SMA, may be increasingly excited by higher frequency stimulation (Jahanshahi et al., 1995). Moreover, there was a statistically significant difference in score when compared to those in the Sham group, consistent with a study conducted by Yokoe et al. (2018). In a meta-analysis examining the efficacy of rTMS on motor function in PD, HFrTMS also demonstrated greater improvement in motor symptoms in patients with PD (Li et al., 2022). In terms of UPDRS-III, no statistically significant differences in score were demonstrated between those resulting from HFrTMS and LFrTMS. A potential explanation is that both HFrTMS and LFrTMS increase cortical inhibitory mechanisms and down-regulate hyperexcitability in the cortical motor layer in Parkinson's patients (Lefaucheur et al., 2004; Fierro et al., 2008). It has been suggested that the long-term positive effects of TMS may stem from differential effects on nerves, neural networks, synapses, and molecular genetics (Chervyakov et al., 2015). In addition, the effect of the dual-modality combination therapy was not significant, there were few relevant RCTs exploring this modality. Hopefully, more RCTs with large samples will soon be available. We conclude that magnetic stimulation, especially high-frequency, was most effective in improving UPDRS-III score in Parkinson's patients.

Changes in TUGT time and FOGQ score in patients with PD were also assessed in our NMA. Both AtDCS and HFrTMS resulted in improvements in TUGT time, whereas LFrTMS resulted in improvements at long-term observation. Among the different stimulation modalities, HFrTMS was superior to both AtDCS and LFrTMS with respect to improvements in long-term TUGT time, whereas it was superior to LFrTMS at short-term observation. According to the SUCRA plot, HFrTMS also showed the highest rank probability in TUGT time. Regarding FOGQ score, there was no statistical difference between those associated with NIBS and Sham surgery, which might be attributed to the limited number of studies and the variability in results stemming from stimulation sites. However, studies conducted by Khedr et al. (2007b) and Strafella et al. (2003) suggest that NIBS may directly activate dopaminergic neurons in the striatum, thereby providing endogenous dopamine. In conclusion, NIBS has the potential to improve motor performance in PD patients, with HFrTMS being the most effective modality for this improvement.

After conducting a detailed subgroup analysis of various stimulation modalities of rTMS and tDCS with specific stimulation locations, we observed that the NIBS stimulation sites were mainly focused on the M1 region, followed by DLPFC and SMA. The overactivation of these regions has been observed in PD patients, stimulating these regions could reduce the activity via hyperdirect pathway that connects several prefrontal regions and subthalamic nucleus, thus improving motor and non-motor symptoms (Nambu et al., 1997; Jang et al., 2012). Among stimulation sites, cumulative high-frequency rTMS on M1 has been suggested as a potential therapy to improve locomotion and motor function in PD patients (Chang et al., 2016). Khedr et al. showed that high-frequency rTMS increased the excitability of M1 and the interaction of related brain regions in healthy volunteers and improved motor performance in PD patients (Khedr et al., 2007a, 2019). In addition, Manor et al. suggested that stimulation of the DLPFC, and M1 regions may benefit some aspects of motor function (Manor et al., 2021). Although some studies reported the participation of SMA in PD, the application of SMA is still limited (Zhu et al., 2015). In pair-wise analysis, we observed comparable improvement in long-term efficacy in SMA region. SMA is a suitable target for HFrTMS and the combination of it with M1 might be a treatment worth trying (Chen and Chen, 2019). Moreover, HFrTMS placed in the SMA region demonstrated significant improvement in FOGQ score in PD. This effect may be due to an increased excitability in underactive SMA regions, leading to a beneficial effect on motor symptoms (Fitzgerald et al., 2006; Mi et al., 2019). However, it should be noted that higher frequency (10 Hz) stimulation may end up with adverse efficacy as overactivation of SMA at rest may lead to homeostatic plasticity (Buhmann et al., 2004; Siebner et al., 2004; Shirota et al., 2013). HFrTMS significantly improved long-term UPDRS-III and TUGT time in PD. Although improvements in patient FOGQ score were not observed with AtDCS, study conducted by Manor et al. found that AtDCS was associated with a reduction in self-reported severity of FOGQ immediately after the intervention (Manor et al., 2021). This finding suggests that tDCS may have a positive effect on FOGQ (although the correlation between experimental testing and self-reporting was low), but a more robust “dose” (i.e., number, intensity, and frequency of stimulation sessions) may be required to cause statistically significant differences in laboratory-based observations.

Overall, rTMS and tDCS are the two most common NIBS techniques currently used in patients with PD. Both techniques are generally well-tolerated, with occasional reports of (typically self-limiting) head and neck pain or tingling. The choice of appropriate treatment options for clinical decision-making should be based on the patient's specific needs. Moreover, although there are currently numerous clinical trials on NIBS, future rigorous large-scale clinical trials considering specific sites and modalities are still required to validate the detailed benefits of NIBS.

There are some limitations in our study. First, our findings may be influenced by the results of some small RCTs, such as the low number of RCTs using specific treatment modalities. Second, the limited sample size of cathodal electrical stimulations may constrain the interpretation of the NMA results. Third, although network heterogeneity and consistency have been confirmed, the statistical power of this relatively weak network remains limited and might be influenced by confounding factors, such as discrepancy of primary care levels between different RCTs. Finally, the combination of the Sham tDCS and Sham rTMS groups was assumed to result in similar placebo effects, despite differences in the specific measures.

## Conclusion

Our study compared the efficacy of different NIBS on motor function in patients with PD, and the findings suggest that HFrTMS is generally more effective than LFrTMS and AtDCS in improving motor function. Furthermore, NIBS was found to be well-tolerated, with few and transient AEs. More RCTs with large sample size that focus on the comparison of different stimulation sites as well as the frequency and duration of stimulation in NIBS are required in the future.

## Data availability statement

The original contributions presented in the study are included in the article/Supplementary material, further inquiries can be directed to the corresponding authors.

## Author contributions

The leading investigators are YQ, ZY, and MW. Study design by ZW, JW, and GC. Data analysis completed by AD and MX. Drafting of the manuscript completed by YQ and ZY. Manuscript revision completed by ZW, JW, and GC. Final version authorized for publication by all authors.
