# Supplementary material for: Motor function improvement and acceptability of non-invasive brain stimulation in patients with Parkinson's disease: a Bayesian network analysis
Source: Front Neurosci. 2023 Jul 26;17:1212640. doi: 10.3389/fnins.2023.1212640 (PMC10410144; doi:10.3389/fnins.2023.1212640)

**Motor function improvement and acceptability of non-invasive brain stimulation in patients with Parkinson's disease: A Bayesian network analysis**

Youjia Qiu^1, #^, Ziqian Yin^2, #^, Menghan Wang^2, #^, Aojie Duan^1^, Minjia Xie^1^, Zhong Wang^1, *^, Jiang Wu^1, *^, Gang Chen^1^

*^1^ Department of Neurosurgery & Brain and Nerve Research Laboratory, The First Affiliated Hospital of Soochow University, Suzhou, Jiangsu Province, 215006, China*

*^2^ Suzhou Medical College of Soochow University, Suzhou, Jiangsu Province, 215002, China*

^#^ Youjia Qiu, Ziqian Yin, and Menghan Wang contribute equally to this work.

*Corresponding author:

Zhong Wang, Department of Neurosurgery, The First Affiliated Hospital of Soochow University, 188 Shizi Street, Suzhou, Jiangsu Province, 215006, China. Email address: wangz8761@163.com.

Jiang Wu, Department of Neurosurgery, The First Affiliated Hospital of Soochow University, 188 Shizi Street, Suzhou, Jiangsu Province, 215006, China. Email address: szjiangwu@163.com.

Table S1: Detailed search strategy.

Table S2: Detailed AEs.

Table S3: Network meta-analysis results of short-term UPDRS-Ⅲ in studies with low heterogeneity.

Table S4: Detailed certainty of evidence for each outcome in league table.

Figure S1: Forest plots of the network meta-analysis: short-term UPDRS-Ⅲ.

Figure S2: Forest plots of the network meta-analysis: long-term UPDRS-Ⅲ.

Figure S3: Forest plots of the network meta-analysis: short-term TUG.

Figure S4: Forest plots of the network meta-analysis: long-term TUG.

Figure S5: Forest plots of the network meta-analysis: short-term FOG.

Figure S6: Forest plots of the network meta-analysis: long-term FOG.

Figure S7 Forest plots for the heterogeneity: short-term UPDRS-Ⅲ.

Figure S8 Forest plots for the heterogeneity: long-term UPDRS-Ⅲ.

Figure S9 Forest plots for the heterogeneity: short-term TUG.

Figure S10 Forest plots for the heterogeneity: long-term TUG.

Figure S11 Forest plots for the heterogeneity: short-term FOG.

Figure S12 Forest plots for the heterogeneity: long-term FOG.

Figure S13 Trace and density of the network meta-analysis: short-term UPDRS-Ⅲ.

Figure S14 Trace and density of the network meta-analysis: long-term UPDRS-Ⅲ.

Figure S15 Trace and density of the network meta-analysis: short-term TUG.

Figure S16 Trace and density of the network meta-analysis: long-term TUG.

Figure S17 Trace and density of the network meta-analysis: short-term FOG.

Figure S18 Trace and density of the network meta-analysis: long-term FOG.

Figure S19 Forest plots for the consistency: short-term UPDRS-Ⅲ.

Figure S20 Forest plots for the consistency: long-term UPDRS-Ⅲ.

Figure S21: Funnel plot for short-term UPDRS-Ⅲ.

Figure S22: Funnel plot for long-term UPDRS-Ⅲ.

Figure S23: Funnel plot for short-term TUG.

Figure S24: Funnel plot for long-term TUG.

Figure S25: Funnel plot for short-term FOG.

Figure S26: Funnel plot for long-term FOG.

Figure S27: Pair-wise meta-analysis of short-term: UPDRS-Ⅲ, MC, NIBS versus Sham.

Figure S28: Pair-wise meta-analysis of short-term: UPDRS-Ⅲ, M1, NIBS versus Sham.

Figure S29: Pair-wise meta-analysis of short-term: UPDRS-Ⅲ, SMA, NIBS versus Sham.

Figure S30: Pair-wise meta-analysis of short-term: UPDRS-Ⅲ, DLPFC, NIBS versus Sham.

Figure S31: Pair-wise meta-analysis of short-term: UPDRS-Ⅲ, M1_DLPFC, NIBS versus Sham.

Figure S32: Pair-wise meta-analysis of short-term: UPDRS-Ⅲ, Cerebellum, NIBS versus Sham.

Figure S33: Pair-wise meta-analysis of short-term: TUG, MC, NIBS versus Sham.

Figure S34: Pair-wise meta-analysis of short-term: TUG, M1, NIBS versus Sham.

Figure S35: Pair-wise meta-analysis of short-term: TUG, SMA, NIBS versus Sham.

Figure S36: Pair-wise meta-analysis of short-term: TUG, DLPFC, NIBS versus Sham.

Figure S37: Pair-wise meta-analysis of short-term: TUG, M1_DLPFC, NIBS versus Sham.

Figure S38: Pair-wise meta-analysis of short-term: TUG, Cerebellum, NIBS versus Sham.

Figure S39: Pair-wise meta-analysis of short-term: FOG, M1, NIBS versus Sham.

Figure S40: Pair-wise meta-analysis of short-term: FOG, SMA, NIBS versus Sham.

Figure S41: Pair-wise meta-analysis of short-term: FOG, M1_DLPFC, NIBS versus Sham.

Figure S42: Pair-wise meta-analysis of long-term: UPDRS-Ⅲ, MC, NIBS versus Sham.

Figure S43: Pair-wise meta-analysis of long-term: UPDRS-Ⅲ, M1, NIBS versus Sham.

Figure S44: Pair-wise meta-analysis of long-term: UPDRS-Ⅲ, SMA, NIBS versus Sham.

Figure S45: Pair-wise meta-analysis of long-term: UPDRS-Ⅲ, DLPFC, NIBS versus Sham.

Figure S46: Pair-wise meta-analysis of long-term: UPDRS-Ⅲ, M1_DLPFC, NIBS versus Sham.

Figure S47: Pair-wise meta-analysis of long-term: TUG, MC, NIBS versus Sham.

Figure S48: Pair-wise meta-analysis of long-term: TUG, M1, NIBS versus Sham.

Figure S49: Pair-wise meta-analysis of long-term: TUG, SMA, NIBS versus Sham.

Figure S50: Pair-wise meta-analysis of long-term: TUG, DLPFC, NIBS versus Sham.

Figure S51: Pair-wise meta-analysis of long-term: TUG, M1_DLPFC, NIBS versus Sham.

Figure S52: Pair-wise meta-analysis of long-term: FOG, M1, NIBS versus Sham.

Figure S53: Pair-wise meta-analysis of long-term: FOG, SMA, NIBS versus Sham.

Figure S54: Pair-wise meta-analysis of long-term: FOG, M1_DLPFC, NIBS versus Sham.

Figure S55: PRSIMA checklist

**Table S1: Detailed Search Strategy**

Search Date: 1 January 2023

**Pubmed：**

| Search | Query | Results |
| --- | --- | --- |
| #1 | ((((((((((Magnetic Stimulation, Transcranial[Title/Abstract])) OR (Magnetic Stimulations, Transcranial[Title/Abstract])) OR (Stimulation, Transcranial Magnetic[Title/Abstract])) OR (Stimulations, Transcranial Magnetic[Title/Abstract])) OR (Transcranial Magnetic Stimulations[Title/Abstract])) OR (Transcranial Magnetic Stimulation, Single Pulse[Title/Abstract])) OR (Transcranial Magnetic Stimulation, Paired Pulse[Title/Abstract])) OR (Transcranial Magnetic Stimulation, Repetitive[Title/Abstract])) OR (rTMS[Title/Abstract])) OR "Transcranial Magnetic Stimulation"[Mesh]) | 21,123 |
| #2 | (((Transcranial Direct Current Stimulation [MeSH Terms]) OR (Direct Current Stimulation, Transcranial [Title/Abstract])) OR (tDCS [Title/Abstract])) OR (noninvasive brain stimulation [Title/Abstract]) | 8044 |
| #3 | #1 OR #2 | 27,072 |
| #4 | ((((((((((((((((((Idiopathic Parkinson's Disease[Title/Abstract])) OR (Lewy Body Parkinson's Disease[Title/Abstract])) OR (Parkinson's Disease, Idiopathic[Title/Abstract])) OR (Parkinson's Disease, Lewy Body[Title/Abstract])) OR (Parkinson Disease, Idiopathic[Title/Abstract])) OR (Parkinson's Disease[Title/Abstract])) OR (Idiopathic Parkinson Disease[Title/Abstract])) OR (Lewy Body Parkinson Disease[Title/Abstract])) OR (Primary Parkinsonism[Title/Abstract])) OR (Parkinsonism, Primary[Title/Abstract])) OR (Paralysis Agitans[Title/Abstract])) OR (PD[Title/Abstract])) OR "Parkinson Disease"[Mesh]) | 246,862 |
| #5 | #3 AND #4 | 1,010 |
| #6 | #5 AND (((((((((((Randomized Controlled Trial[MeSH Terms]) OR (controlled clinical trial[Title/Abstract])) OR (random allocation[Title/Abstract])) OR (double-blind[Title/Abstract])) OR (single-blind[Title/Abstract])) OR (Placebo[Title/Abstract])) OR (Randomly[Title/Abstract])) OR (randomized[Title/Abstract])) OR (clinical trial[Title/Abstract])) OR (RCT[Title/Abstract])) OR (random[Title/Abstract])) | 258 |

**Embass：**

| Search | Query | Results |
| --- | --- | --- |
| #1 | ' Transcranial Magnetic Stimulation '/exp | 29,591 |
| #2 | ' rTMS ':ab,ti | 8,948 |
| #3 | ' Transcranial Magnetic Stimulation, Repetitive ':ab,ti | 13 |
| #4 | ' Transcranial Magnetic Stimulation, Paired Pulse ':ab,ti | 3 |
| #5 | ' Transcranial Magnetic Stimulation, Single Pulse ':ab,ti | 3 |
| #6 | ' Transcranial Magnetic Stimulations ':ab,ti | 91 |
| #7 | ' Stimulation, Transcranial Magnetic ':ab,ti | 83 |
| #8 | ' Magnetic Stimulation, Transcranial ':ab,ti | 79 |
| #9 | ' Magnetic Stimulations, Transcranial ':ab,ti | 0 |
| #10 | ' Stimulations, Transcranial Magnetic ':ab,ti | 1 |
| #11 | #1 OR #2 OR #3 OR #4 OR #5 OR #6 OR #7 OR #8 OR #9 OR #10 | 30,507 |
| #12 | ' Transcranial Direct Current Stimulation '/exp | 9,935 |
| #13 | ' Direct Current Stimulation, Transcranial ':ab,ti | 20 |
| #14 | ' tDCS ':ab,ti | 8,746 |
| #15 | ' noninvasive brain stimulation ':ab,ti | 1,139 |
| #16 | #12 OR #13 OR #14 OR #15 | 11,929 |
| #17 | #11 OR #16 | 39,583 |
| #18 | ' Parkinson Disease '/exp | 187,268 |
| #19 | ' PD ':ab,ti | 291,130 |
| #20 | ' Paralysis Agitans ':ab,ti | 202 |
| #21 | ' Parkinsonism, Primary ':ab,ti | 6 |
| #22 | ' Primary Parkinsonism ':ab,ti | 35 |
| #23 | ' Lewy Body Parkinson Disease ':ab,ti | 5 |
| #24 | ' Idiopathic Parkinsons Disease ':ab,ti | 39 |
| #25 | ' Lewy Body Parkinsons Disease ':ab,ti | 0 |
| #26 | ' Parkinsons Disease, Idiopathic ':ab,ti | 1 |
| #27 | ' Parkinsons Disease, Lewy Body ':ab,ti | 1 |
| #28 | ' Parkinson Disease ':ab,ti | 18,162 |
| #29 | ' Parkinson Disease, Idiopathic ':ab,ti | 3 |
| #30 | ' Idiopathic Parkinson Disease ':ab,ti | 484 |
| #31 | #18 OR #19 OR #20 OR #21 OR #22 OR #23 OR #24 OR #25 OR #26 OR #27 OR #28 OR #29 OR #30 | 396,287 |
| #32 | #17 AND #31 | 2,116 |
| #33 | 'randomized controlled trial':ab,ti OR 'randomized':ab,ti OR 'placebo':ab,ti OR 'RCT':ab,ti | 1,107,747 |
| #34 | #32 AND #33 | 417 |

**Cochrane:**

| Search | Query | Results |
| --- | --- | --- |
| #1 | MeSH descriptor: [Transcranial Magnetic Stimulation] explode all trees | 1694 |
| #2 | (rTMS):ti,ab,kw OR (Transcranial Magnetic Stimulation, Repetitive):ti,ab,kw OR (Transcranial Magnetic Stimulation, Paired Pulse):ti,ab,kw OR (Transcranial Magnetic Stimulation, Single Pulse):ti,ab,kw OR (Magnetic Stimulation, Transcranial):ti,ab,kw OR (Magnetic Stimulations, Transcranial):ti,ab,kw OR (Stimulation, Transcranial Magnetic):ti,ab,kw OR (Stimulations, Transcranial Magnetic):ti,ab,kw OR (Transcranial Magnetic Stimulations):ti,ab,kw | 7248 |
| #3 | #1 or #2 | 7248 |
| #4 | MeSH descriptor: [Transcranial Direct Current Stimulation] explode all trees | 1073 |
| #5 | (Direct Current Stimulation, Transcranial):ti,ab,kw OR (tDCS):ti,ab,kw OR (noninvasive brain stimulation):ti,ab,kw OR (Transcranial Direct Current Stimulation):ti,ab,kw | 6655 |
| #6 | #4 or #5 | 6655 |
| #7 | MeSH descriptor: [Parkinson Disease] explode all trees | 4831 |
| #8 | (Idiopathic Parkinson's Disease):ab,ti,kw or (Lewy Body Parkinson's Disease):ab,ti,kw or (Parkinson's Disease, Idiopathic):ab,ti,kw or (Parkinson's Disease, Lewy Body):ab,ti,kw or (Parkinson Disease, Idiopathic):ab,ti,kw or (Parkinson's Disease):ab,ti,kw or (Idiopathic Parkinson Disease):ab,ti,kw or (Lewy Body Parkinson Disease):ab,ti,kw or (Primary Parkinsonism):ab,ti,kw or (Parkinsonism, Primary):ab,ti,kw or (Paralysis Agitans):ab,ti,kw or (PD):ab,ti,kw | 45957 |
| #9 | #7 OR #8 | 45957 |
| #10 | #3 OR #6 | 12354 |
| #11 | #10 AND #9 | 692 |

Table S2: Detailed AEs.

| **Study** | **Headache and neck pain** | **Burning sensation** | **Description** |
| --- | --- | --- | --- |
| **rTMS** |  |  |  |
| **Yang(2013)** | - | - | Unreported |
| **Maruo(2013)** | - | - | Unreported |
| **Kim(2015)** | 1 | 0 | One patient reported a mild headache, which disappeared soon after stopping stimulation. |
| **Chang(2016)** | - | - | Unreported |
| **Makkos(2016)** | - | - | Unreported |
| **Brys(2016)** | 34 | 0 | rTMS was well tolerated by all participants, although 34(68% of completers) reported adverse events, most commonly headache and neck pain, which were mild and transient. |
| **Chang(2017)** | 1 | 0 | In the rTMS group, one patient reported a mild headache during the 2nd rTMS session which disappeared soon after the stimulation was discontinued and the patient completed the study with no other adverse effects. |
| **Yokoe(2017)** | - | - | Unreported |
| **Cohen(2018)** | 6 | 0 | The most common side effects were headache (five patients in the rDTMS group and one in the sham group), dizziness (four patients in the rDTMS group), pain in the head or neck during treatment (three patients in the rDTMS group and one in the sham group), nausea, general weakness, and transient aggravation of gait disturbance (two patients in each group). |
| **Aftanas(2018)** | - | - | Unreported |
| **Mi(2019)** | - | - | Unreported |
| **Khedr(2019)** | - | - | Unreported |
| **Khedr2(2019)** | 1 | 0 | 1 developed headache and insomnia and the other 2 refused to stay in the hospital. |
| **Li(2020)** | 6 | 0 | Besides two patients withdrew due to headaches and dizziness in each group, no serious adverse events occurred. The most common adverse events were headaches for 0.5–2 h (3 cases in each group),dizziness for 5–10 min (2 cases in each group), tinnitus for 15–30 min (2 cases in the rTMS group and 1 in the sham stimulation group), and transient aggravation of gait disturbances (1case in the rTMS group). |
| **Zhuang(2020)** | 2 | 0 | Two female participants in the active group reported transient mild headache during stimulation but were relieved soon. |
| **Chung(2020)** | - | - | Unreported |
| **Aftanas2(2022)** | - | - | Unreported |
| **Kaski(2014)** | - | - | Unreported |
| **Biundo(2015)** | - | - | Unreported |
| **Ferrucci(2016)** | - | - | Unreported |
| **Manenti(2016)** | - | - | Unreported |
| **Costa-Ribeiro(2016)** | - | - | Unreported |
| **Yotnuengnit(2017)** | 0 | 2 | During the intervention period, two participants, who received the anodal tDCS intervention for the first time, reported a burning sensation on their forehead where the stimulator was attached. As the day progressed, it subsided without any treatment. |
| **Wong(2022)** | - | - | Unreported |
| **Manor(2021)** | - | - | Unreported |
| **Lee (2021)** | - | - | Unreported |
| **Na(2022)** | - | - | Unreported |
| **Icco(2022)** | - | - | Unreported |
|  |  |  |  |

Table S3: Network meta-analysis results of short-term UPDRS-Ⅲ in studies with low heterogeneity.

| AtDCS |  |  |  |  |  |  |
| --- | --- | --- | --- | --- | --- | --- |
| -0.04 (-6.1, 6) | AtDCS_CtDCS |  |  |  |  |  |
| **3.75 (2.13, 5.36)** | 3.79 (-2.37, 9.92) | HFrTMS |  |  |  |  |
| 3.93 (-0.51, 8.47) | 4 (-3.46, 11.42) | 0.19 (-3.98, 4.4) | HFrTMS_AtDCS |  |  |  |
| 0.92 (-0.6, 2.44) | 0.96 (-5.13, 7.1) | **-2.82 (-4.77, -0.89)** | -3.01 (-7.66, 1.59) | HFrTMS_LFrTMS |  |  |
| 2.5 (-1.71, 6.72) | 2.57 (-4.69, 9.82) | -1.24 (-5.4, 2.91) | -1.44 (-7.36, 4.48) | 1.58 (-2.76, 5.93) | LFrTMS |  |
| -0.16 (-0.92, 0.6) | -0.12 (-6.1, 5.87) | **-3.91 (-5.34, -2.49)** | -4.09 (-8.56, 0.29) | -1.08 (-2.4, 0.23) | -2.67 (-6.8, 1.47) | Sham |

i2.pair i2.cons

22.01104 22.31528

Table S4: Detailed certainty of evidence for each outcome in league table.

| **Comparison** | **Number of studies** | **Within-study bias** | **Reporting bias** | **Indirectness** | **Imprecision** | **Heterogeneity** | **Incoherence** | **Confidence rating** | **Reason(s) for downgrading** |
| --- | --- | --- | --- | --- | --- | --- | --- | --- | --- |
| **Short-term UPDRS-Ⅲ** | | | | | | | | | |
| AtDCS:AtDCS_CtDCS | 0 | No concerns | Low risk | Some concerns | Major concerns | Some concerns | No concerns | Low | Imprecision,Heterogeneity |
| AtDCS:HFrTMS | 0 | No concerns | Low risk | Some concerns | Some concerns | No concerns | No concerns | High | [] |
| AtDCS:HFrTMS_AtDCS | 0 | No concerns | Low risk | Some concerns | Some concerns | No concerns | No concerns | High | [] |
| AtDCS:HFrTMS_LFrTMS | 0 | No concerns | Low risk | Some concerns | Major concerns | No concerns | No concerns | Moderate | Imprecision |
| AtDCS:LFrTMS | 0 | No concerns | Low risk | Some concerns | Some concerns | No concerns | No concerns | High | [] |
| AtDCS:Sham | 7 | No concerns | Low risk | No concerns | Major concerns | No concerns | Some concerns | Low | Imprecision,Incoherence |
| AtDCS_CtDCS:HFrTMS | 0 | No concerns | Low risk | Some concerns | Some concerns | No concerns | No concerns | High | [] |
| AtDCS_CtDCS:HFrTMS_AtDCS | 0 | No concerns | Low risk | Some concerns | Some concerns | No concerns | No concerns | High | [] |
| AtDCS_CtDCS:HFrTMS_LFrTMS | 0 | No concerns | Low risk | Some concerns | Major concerns | No concerns | No concerns | Moderate | Imprecision |
| AtDCS_CtDCS:LFrTMS | 0 | No concerns | Low risk | Some concerns | Major concerns | No concerns | No concerns | Moderate | Imprecision |
| AtDCS_CtDCS:Sham | 1 | No concerns | Low risk | No concerns | Some concerns | No concerns | No concerns | High | [] |
| HFrTMS:HFrTMS_AtDCS | 1 | No concerns | Low risk | No concerns | Some concerns | No concerns | No concerns | High | [] |
| HFrTMS:HFrTMS_LFrTMS | 0 | No concerns | Low risk | Some concerns | Major concerns | No concerns | No concerns | Moderate | Imprecision |
| HFrTMS:LFrTMS | 2 | No concerns | Low risk | No concerns | Some concerns | No concerns | Major concerns | High | [] |
| HFrTMS:Sham | 11 | No concerns | Low risk | No concerns | Some concerns | No concerns | No concerns | High | [] |
| HFrTMS_AtDCS:HFrTMS_LFrTMS | 0 | No concerns | Low risk | Some concerns | Major concerns | Some concerns | Major concerns | Very low | Imprecision,Heterogeneity,Incoherence |
| HFrTMS_AtDCS:LFrTMS | 0 | No concerns | Low risk | Some concerns | Major concerns | No concerns | No concerns | Moderate | Imprecision |
| HFrTMS_AtDCS:Sham | 0 | No concerns | Low risk | Some concerns | Major concerns | No concerns | No concerns | Moderate | Imprecision |
| HFrTMS_LFrTMS:LFrTMS | 0 | No concerns | Low risk | Some concerns | Major concerns | Some concerns | Major concerns | Very low | Imprecision,Heterogeneity,Incoherence |
| HFrTMS_LFrTMS:Sham | 1 | No concerns | Low risk | No concerns | Major concerns | No concerns | No concerns | Moderate | Imprecision |
| LFrTMS:Sham | 2 | No concerns | Low risk | No concerns | Some concerns | No concerns | No concerns | High | [] |
| **long-term UPDRS-Ⅲ** | | | | | | | | | |
| AtDCS:AtDCS_CtDCS | 0 | No concerns | Low risk | Some concerns | Some concerns | No concerns | No concerns | High | [] |
| AtDCS:HFrTMS | 0 | No concerns | Low risk | Some concerns | Some concerns | No concerns | No concerns | High | [] |
| AtDCS:HFrTMS_AtDCS | 0 | No concerns | Low risk | Some concerns | Some concerns | No concerns | No concerns | High | [] |
| AtDCS:LFrTMS | 0 | No concerns | Low risk | Some concerns | Some concerns | No concerns | Some concerns | Moderate | Incoherence |
| AtDCS_CtDCS:HFrTMS | 0 | No concerns | Low risk | Some concerns | Some concerns | No concerns | No concerns | High | [] |
| AtDCS_CtDCS:HFrTMS_AtDCS | 0 | No concerns | Low risk | Some concerns | Some concerns | No concerns | No concerns | High | [] |
| AtDCS_CtDCS:LFrTMS | 0 | No concerns | Low risk | Some concerns | Some concerns | No concerns | No concerns | High | [] |
| HFrTMS_AtDCS:LFrTMS | 0 | No concerns | Low risk | Some concerns | Some concerns | No concerns | No concerns | High | [] |
| HFrTMS_AtDCS:Sham | 0 | No concerns | Low risk | Some concerns | Some concerns | No concerns | No concerns | High | [] |
| AtDCS_CtDCS:Sham | 1 | No concerns | Low risk | No concerns | Major concerns | Some concerns | No concerns | Low | Imprecision，Heterogeneity |
| HFrTMS:HFrTMS_AtDCS | 1 | No concerns | Low risk | No concerns | Some concerns | No concerns | No concerns | High | [] |
| HFrTMS:LFrTMS | 2 | No concerns | Low risk | No concerns | Some concerns | No concerns | No concerns | High | [] |
| LFrTMS:Sham | 2 | No concerns | Low risk | No concerns | Some concerns | No concerns | No concerns | High | [] |
| AtDCS:Sham | 6 | No concerns | Low risk | No concerns | Major concerns | Some concerns | No concerns | Low | Imprecision，Heterogeneity |
| HFrTMS:Sham | 8 | No concerns | Low risk | No concerns | Some concerns | No concerns | No concerns | High | [] |
| **Short-term TUG** | | | | | | | | | |
| AtDCS:HFrTMS | 0 | No concerns | Low risk | Some concerns | Some concerns | No concerns | No concerns | High | [] |
| AtDCS:HFrTMS_AtDCS | 0 | No concerns | Low risk | Some concerns | Some concerns | No concerns | No concerns | High | [] |
| AtDCS:HFrTMS_LFrTMS | 0 | No concerns | Low risk | Some concerns | Some concerns | No concerns | Some concerns | Moderate | Incoherence |
| AtDCS:LFrTMS | 0 | No concerns | Low risk | Some concerns | Some concerns | No concerns | No concerns | High | [] |
| HFrTMS:HFrTMS_LFrTMS | 0 | No concerns | Low risk | Some concerns | Some concerns | No concerns | No concerns | High | [] |
| HFrTMS_AtDCS:HFrTMS_LFrTMS | 0 | No concerns | Low risk | Some concerns | Major concerns | No concerns | No concerns | Moderate | Imprecision |
| HFrTMS_AtDCS:LFrTMS | 0 | No concerns | Low risk | Some concerns | Major concerns | No concerns | No concerns | Moderate | Imprecision |
| HFrTMS_AtDCS:Sham | 0 | No concerns | Low risk | Some concerns | Major concerns | No concerns | No concerns | Moderate | Imprecision |
| HFrTMS_LFrTMS:LFrTMS | 0 | No concerns | Low risk | Some concerns | Some concerns | No concerns | No concerns | High | [] |
| HFrTMS:HFrTMS_AtDCS | 1 | No concerns | Low risk | No concerns | Major concerns | No concerns | Some concerns | Low | Imprecision,Incoherence |
| HFrTMS:LFrTMS | 1 | No concerns | Low risk | No concerns | Some concerns | No concerns | No concerns | High | [] |
| HFrTMS_LFrTMS:Sham | 1 | No concerns | Low risk | No concerns | Major concerns | No concerns | No concerns | Moderate | Imprecision |
| LFrTMS:Sham | 1 | No concerns | Low risk | No concerns | Major concerns | No concerns | No concerns | Moderate | Imprecision |
| AtDCS:Sham | 6 | No concerns | Low risk | No concerns | Some concerns | No concerns | No concerns | High | [] |
| HFrTMS:Sham | 6 | No concerns | Low risk | No concerns | Some concerns | No concerns | No concerns | High | [] |
| **Long-term TUG** | | | | | | | | | |
| AtDCS:HFrTMS | 0 | No concerns | Low risk | Some concerns | No concerns | No concerns | No concerns | High | [] |
| AtDCS:HFrTMS_AtDCS | 0 | No concerns | Low risk | Some concerns | Major concerns | No concerns | No concerns | Moderate | Imprecision |
| AtDCS:LFrTMS | 0 | No concerns | Low risk | Some concerns | Some concerns | No concerns | No concerns | High | [] |
| AtDCS:Sham | 4 | No concerns | Low risk | No concerns | No concerns | No concerns | No concerns | High | [] |
| HFrTMS:HFrTMS_AtDCS | 1 | No concerns | Low risk | No concerns | Major concerns | No concerns | No concerns | Moderate | Imprecision |
| HFrTMS:LFrTMS | 1 | No concerns | Low risk | No concerns | No concerns | No concerns | No concerns | High | [] |
| HFrTMS:Sham | 5 | No concerns | Low risk | No concerns | No concerns | No concerns | No concerns | High | [] |
| HFrTMS_AtDCS:LFrTMS | 0 | No concerns | Low risk | Some concerns | Some concerns | No concerns | No concerns | High | [] |
| HFrTMS_AtDCS:Sham | 0 | No concerns | Low risk | Some concerns | Some concerns | No concerns | Some concerns | Moderate | Incoherence |
| LFrTMS:Sham | 1 | No concerns | Low risk | No concerns | Some concerns | No concerns | No concerns | High | [] |
| **Short-term FOG** | | | | | | | | | |
| AtDCS:HFrTMS | 0 | No concerns | Low risk | Some concerns | Some concerns | No concerns | No concerns | High | [] |
| AtDCS:HFrTMS_AtDCS | 0 | No concerns | Low risk | Some concerns | Major concerns | No concerns | Some concerns | Low | Imprecision,Incoherence |
| AtDCS:Sham | 3 | No concerns | Low risk | No concerns | Some concerns | No concerns | No concerns | High | [] |
| HFrTMS:HFrTMS_AtDCS | 1 | No concerns | Low risk | No concerns | Some concerns | No concerns | No concerns | High | [] |
| HFrTMS:Sham | 7 | No concerns | Low risk | No concerns | Some concerns | No concerns | No concerns | High | [] |
| HFrTMS_AtDCS:Sham | 0 | No concerns | Low risk | Some concerns | Some concerns | No concerns | No concerns | High | [] |
| **Long-term FOG** | | | | | | | | | |
| AtDCS:HFrTMS | 0 | No concerns | Low risk | Some concerns | Major concerns | No concerns | No concerns | Moderate | Imprecision |
| AtDCS:HFrTMS_AtDCS | 0 | No concerns | Low risk | Some concerns | Some concerns | No concerns | No concerns | High | [] |
| AtDCS:Sham | 3 | No concerns | Low risk | No concerns | Some concerns | No concerns | No concerns | High | [] |
| HFrTMS:HFrTMS_AtDCS | 1 | No concerns | Low risk | No concerns | Major concerns | No concerns | No concerns | Moderate | Imprecision |
| HFrTMS:Sham | 7 | No concerns | Low risk | No concerns | Some concerns | No concerns | No concerns | High | [] |
| HFrTMS_AtDCS:Sham | 0 | No concerns | Low risk | Some concerns | Some concerns | No concerns | No concerns | High | [] |

Figure S1: Forest plots of the network meta-analysis: short-term UPDRS-Ⅲ.


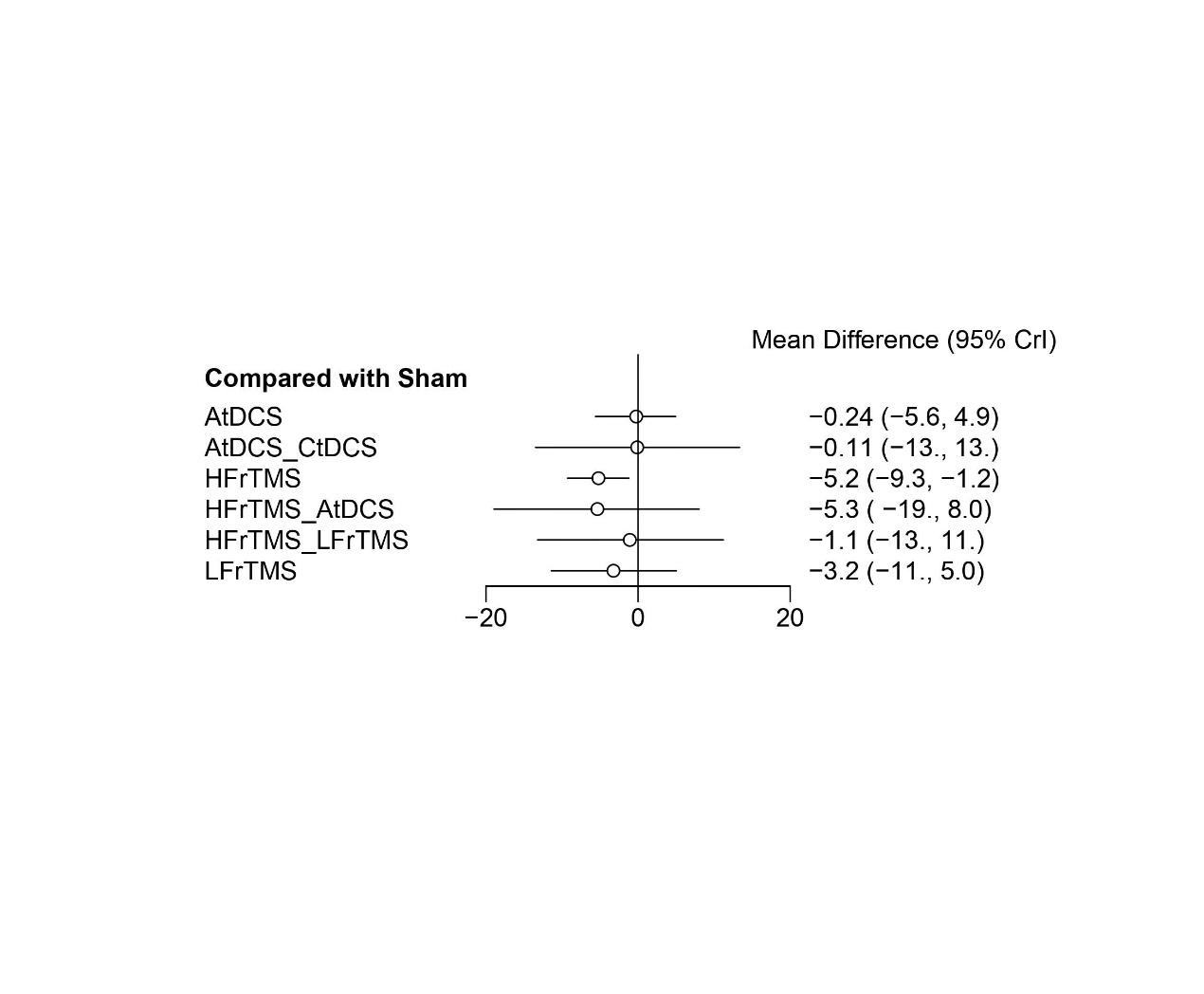


Figure S2: Forest plots of the network meta-analysis: long-term UPDRS-Ⅲ.


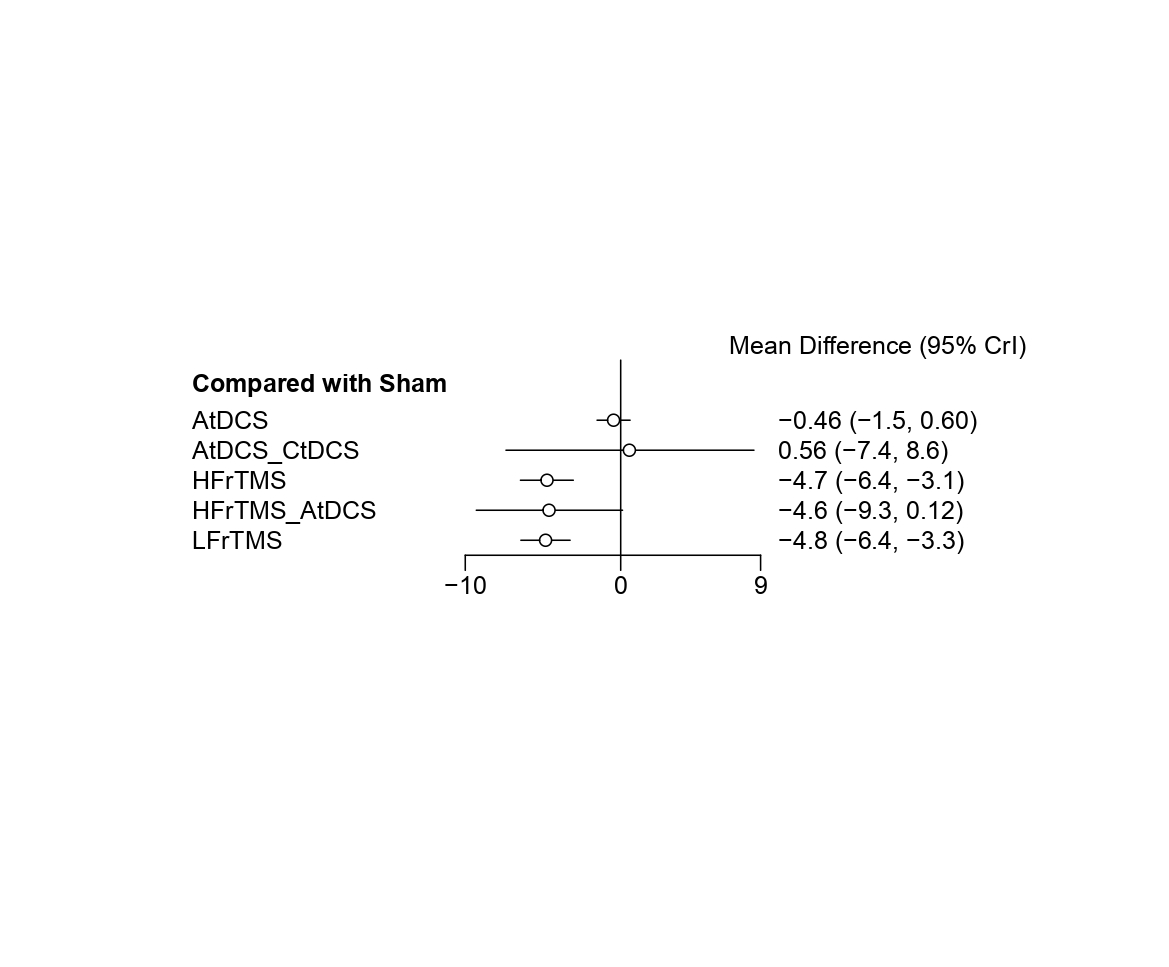


Figure S3: Forest plots of the network meta-analysis: short-term TUG.


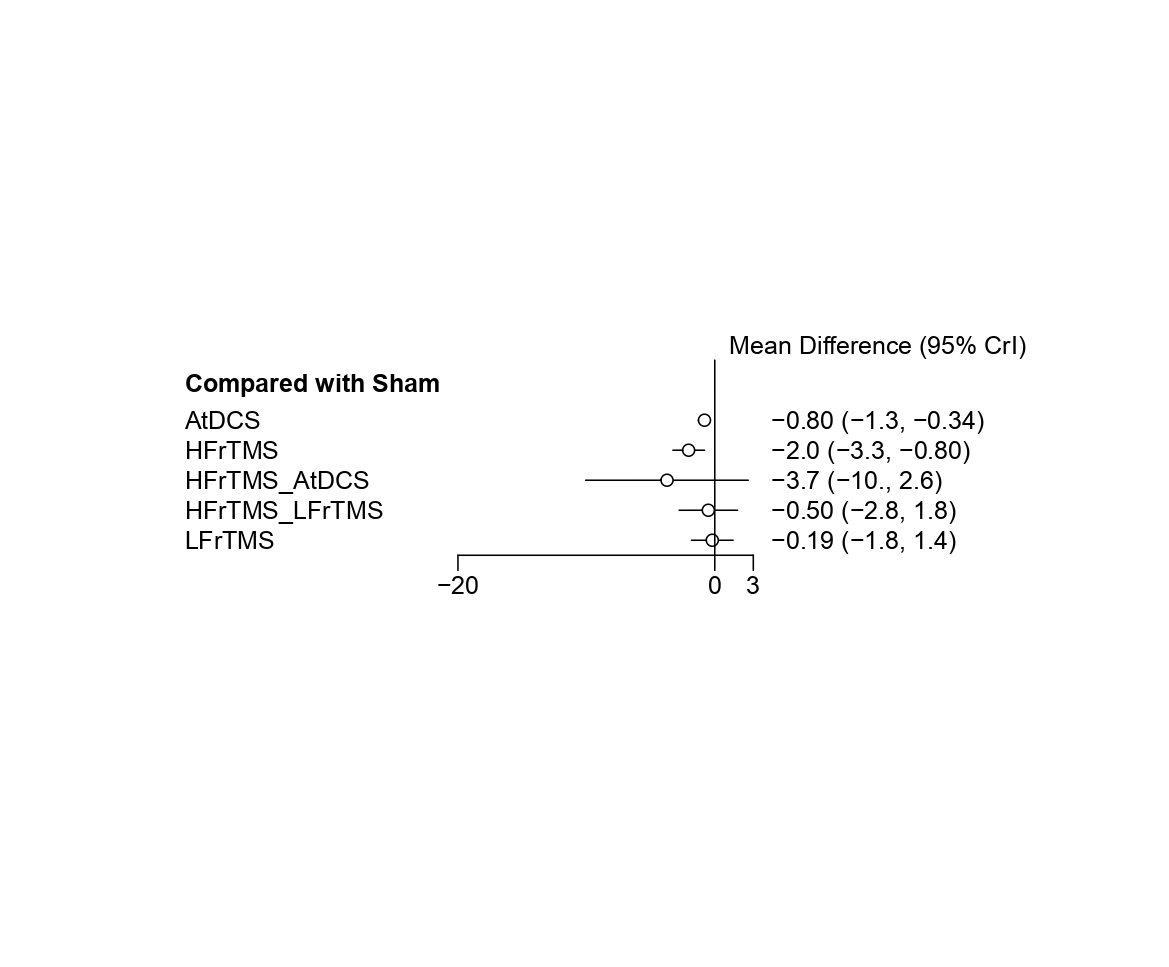


Figure S4: Forest plots of the network meta-analysis: long-term TUG.


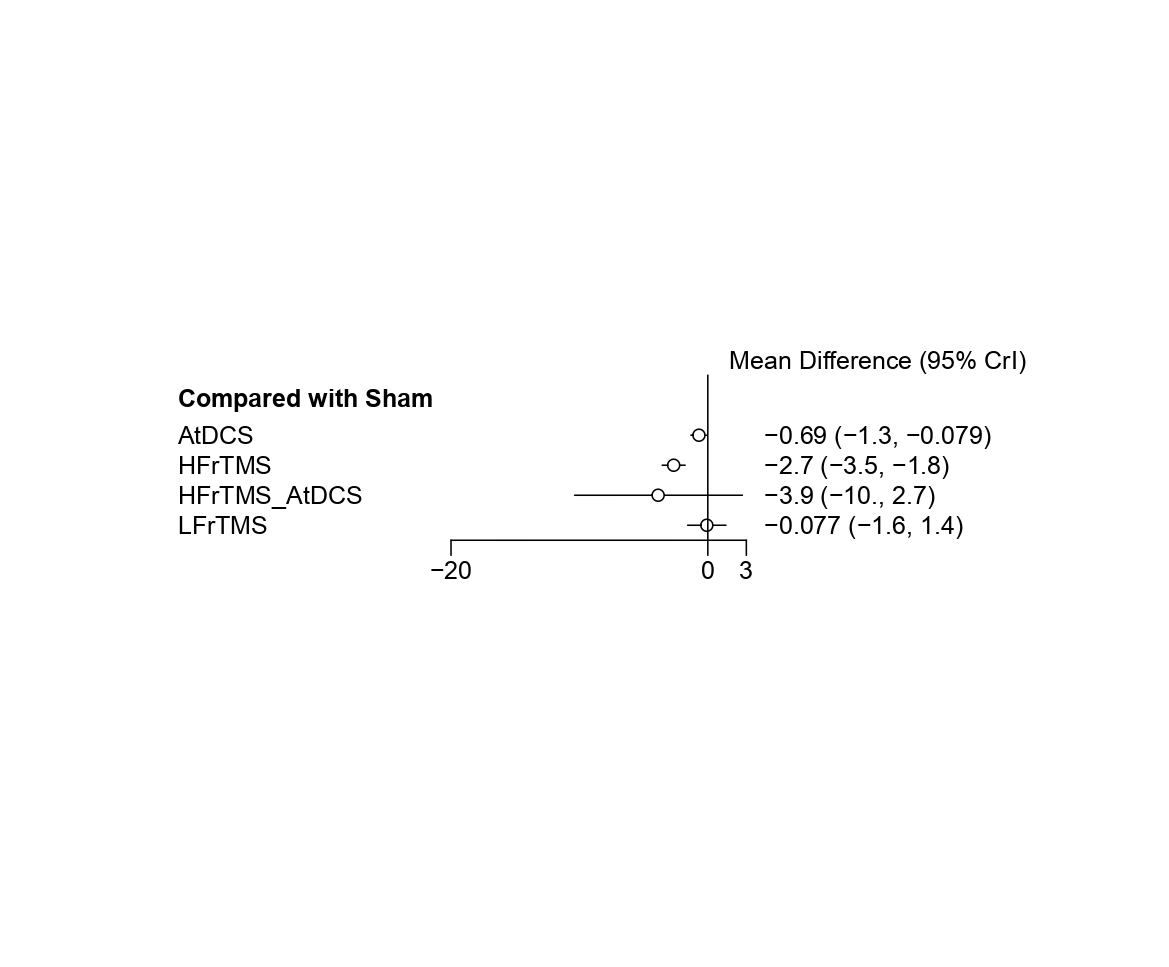


Figure S5: Forest plots of the network meta-analysis: short-term FOG.


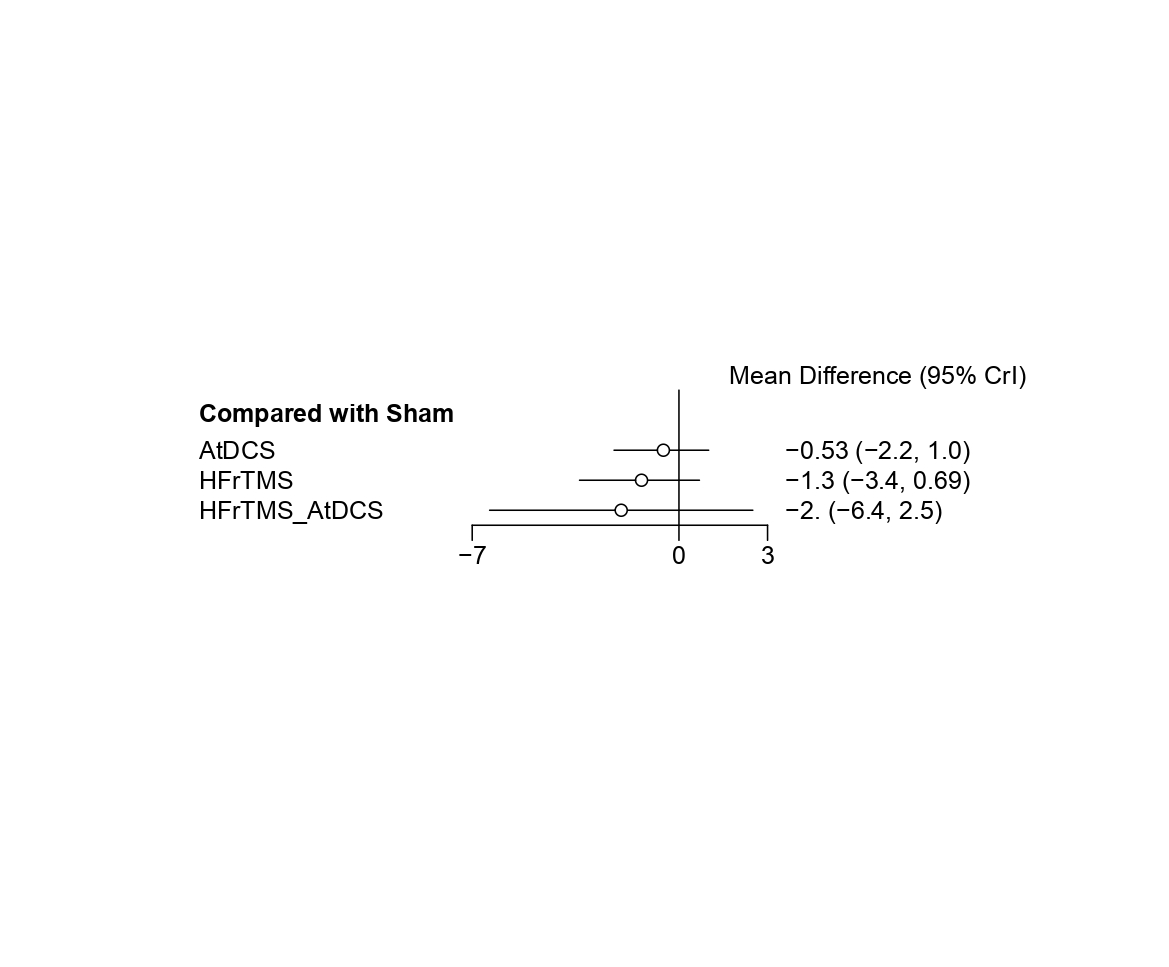


Figure S6: Forest plots of the network meta-analysis: long-term FOG.


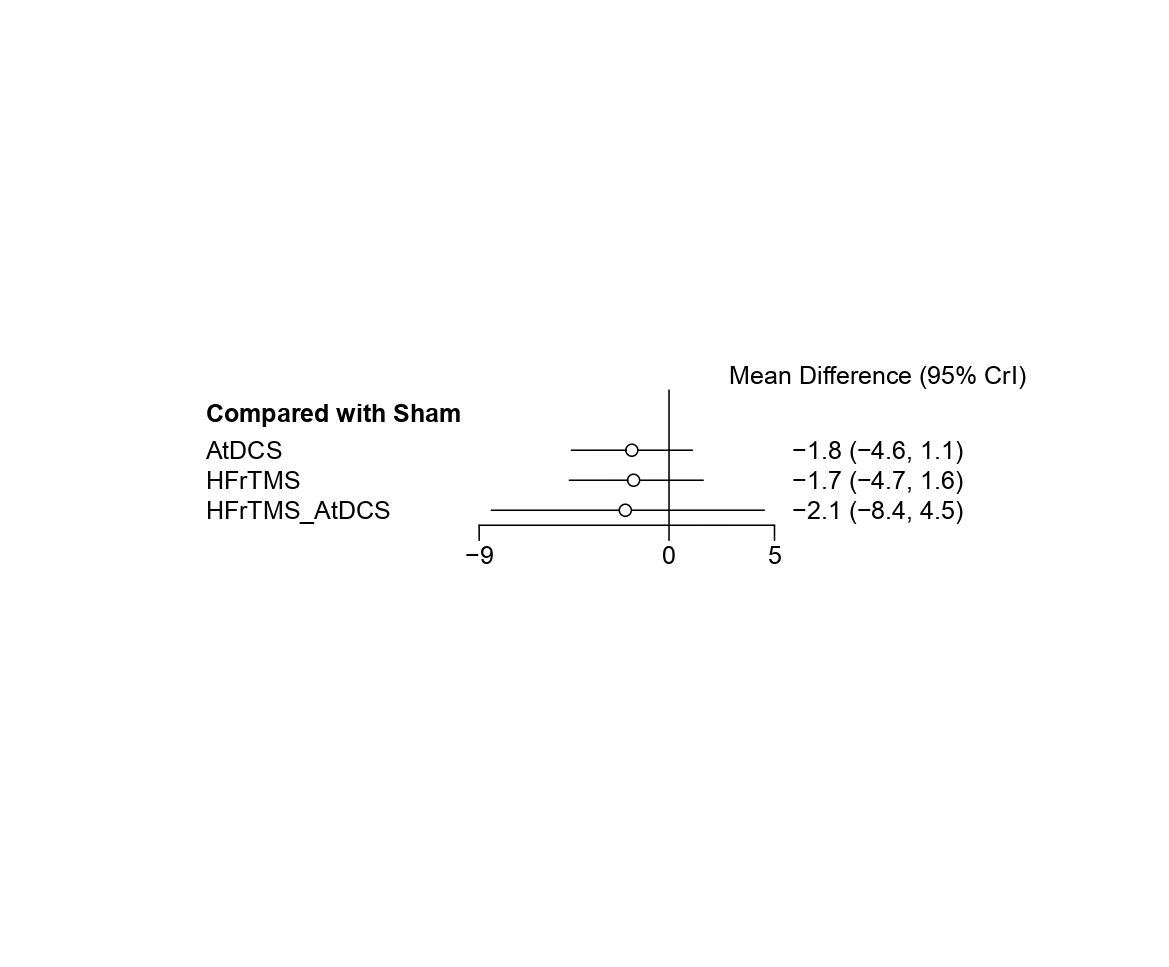


Figure S7 Forest plots for the heterogeneity: short-term UPDRS-Ⅲ.


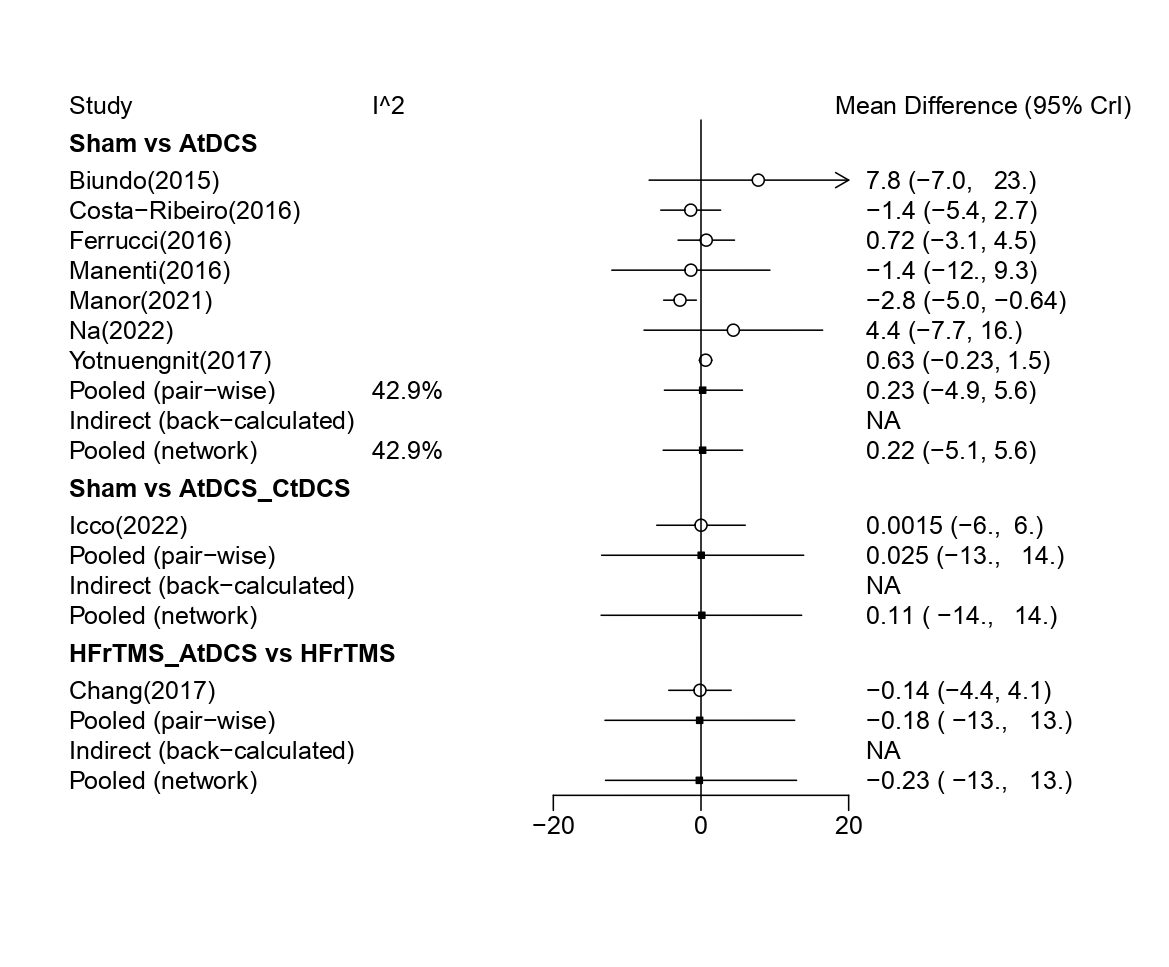


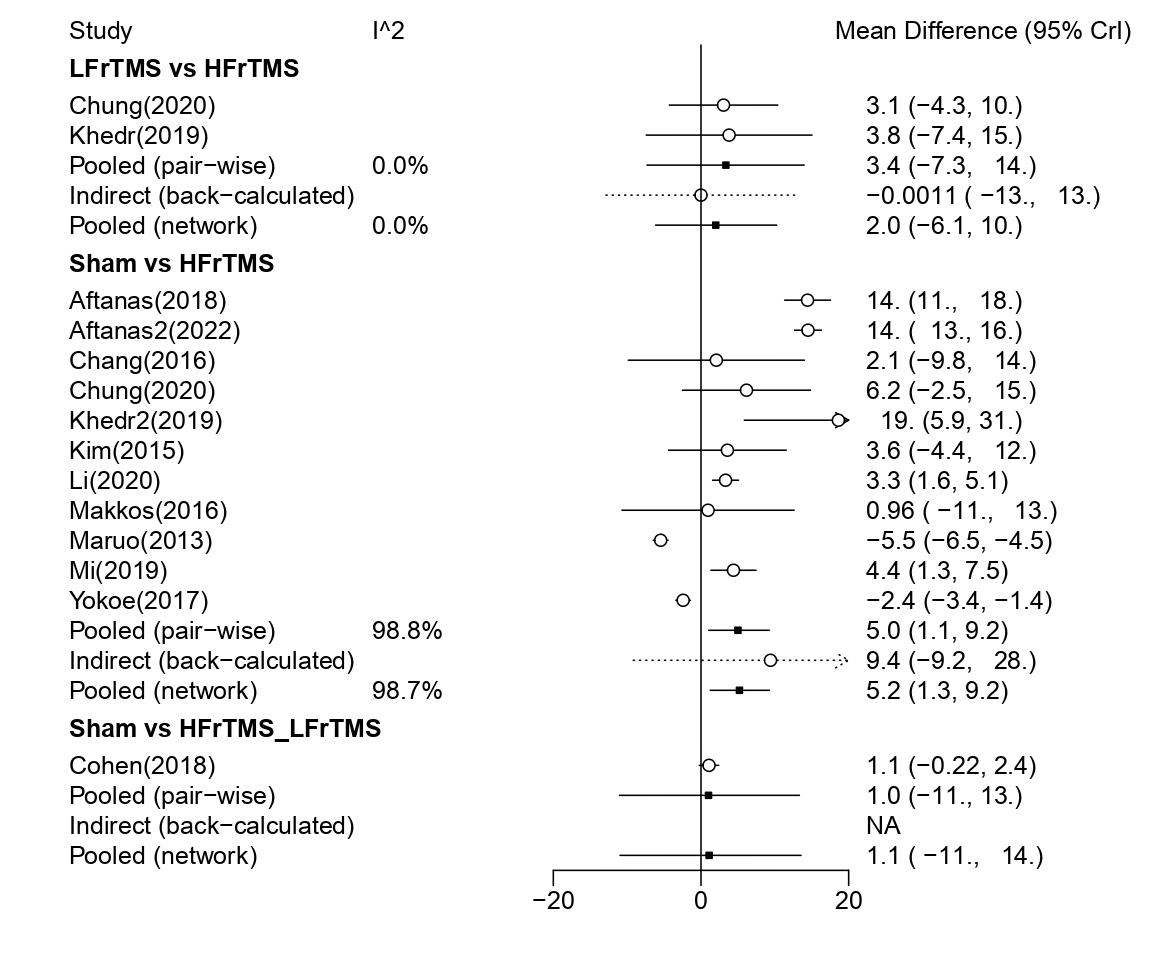


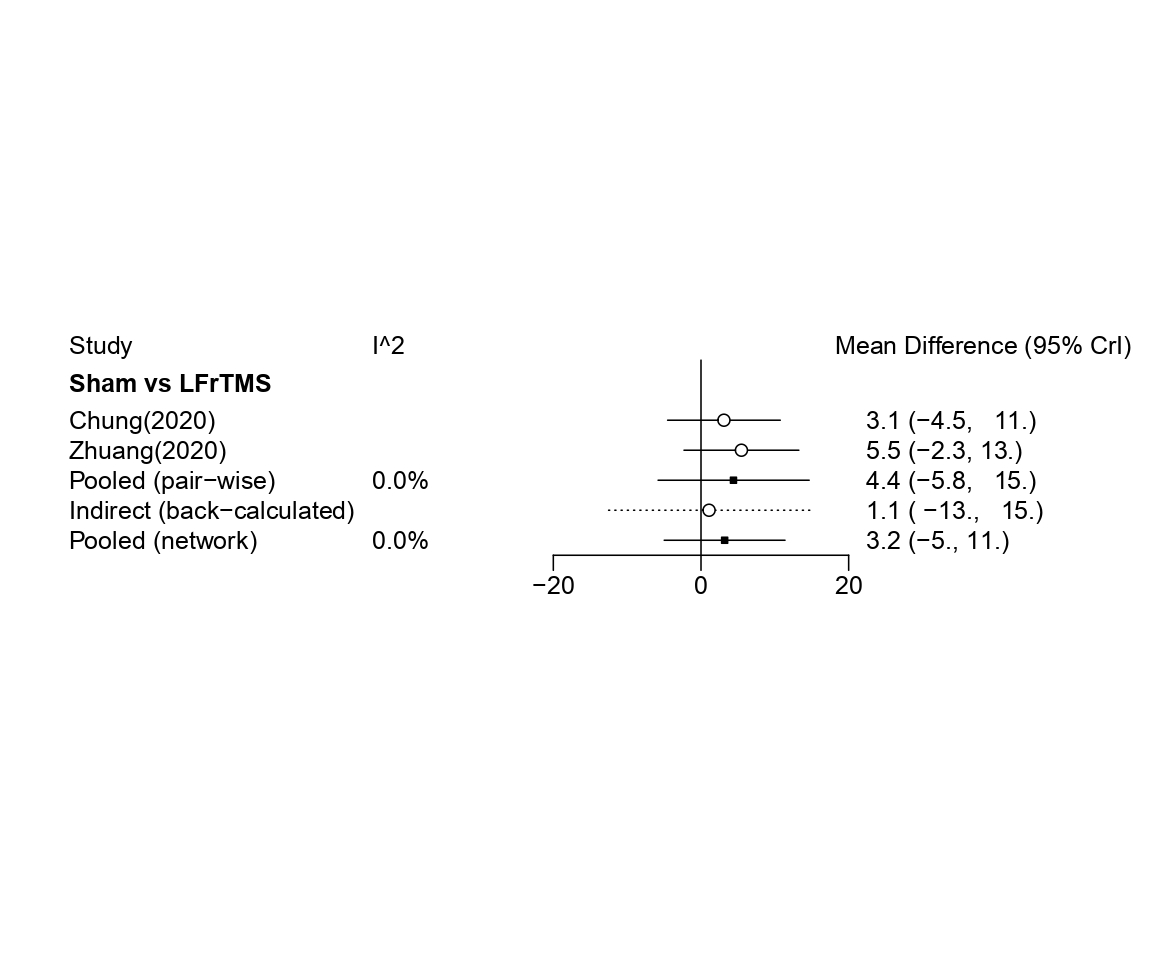


Figure S8 Forest plots for the heterogeneity: long-term UPDRS-Ⅲ.


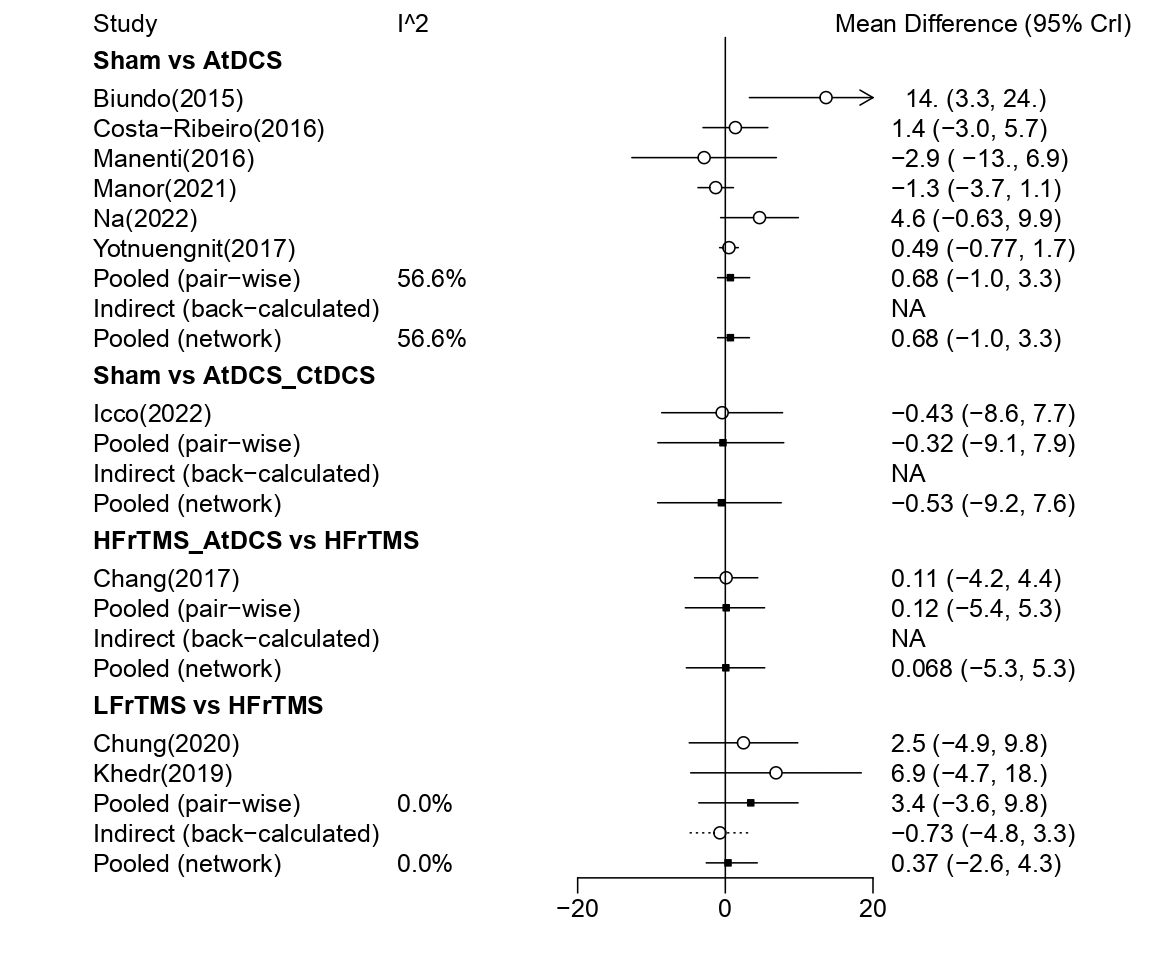


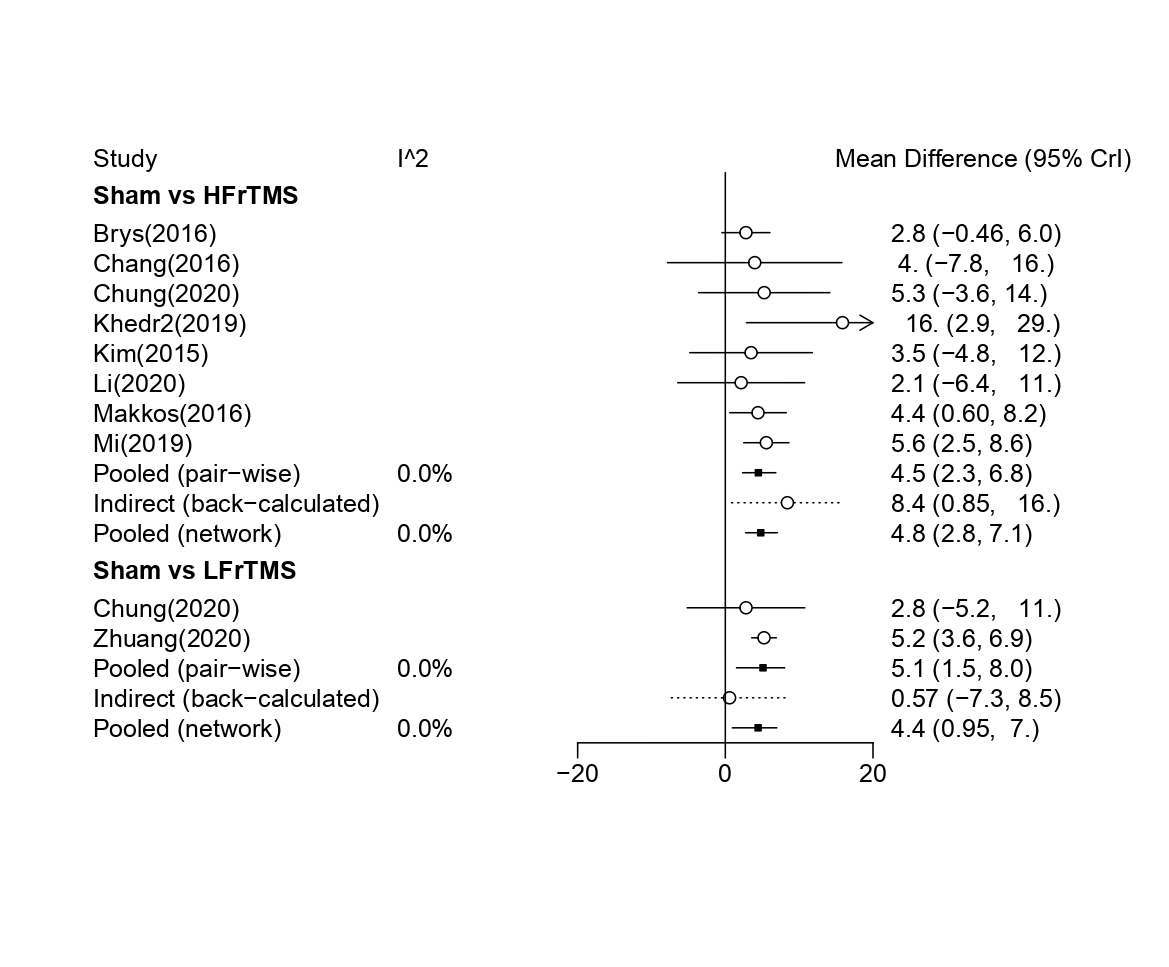


Figure S9 Forest plots for the heterogeneity: short-term TUG.


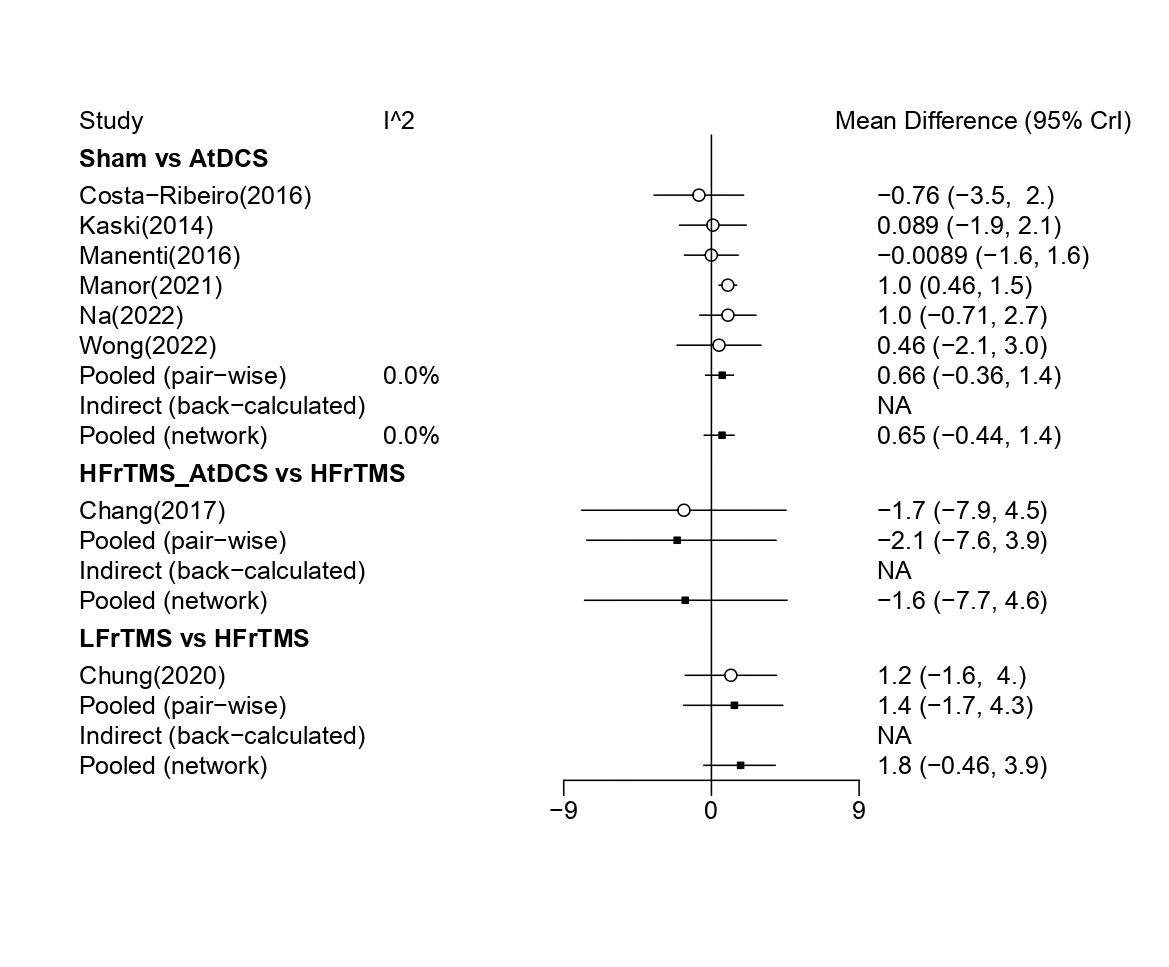


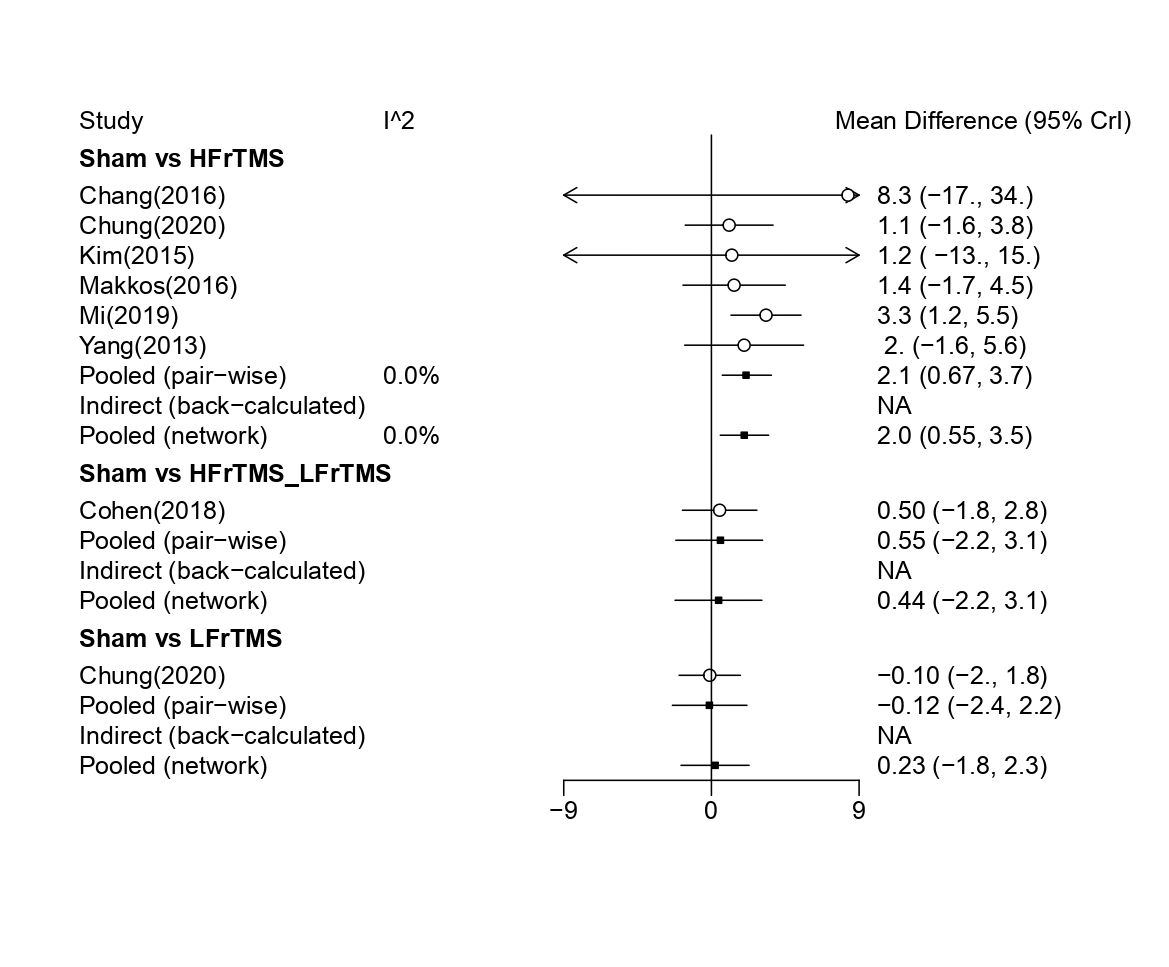


Figure S10 Forest plots for the heterogeneity: long-term TUG.


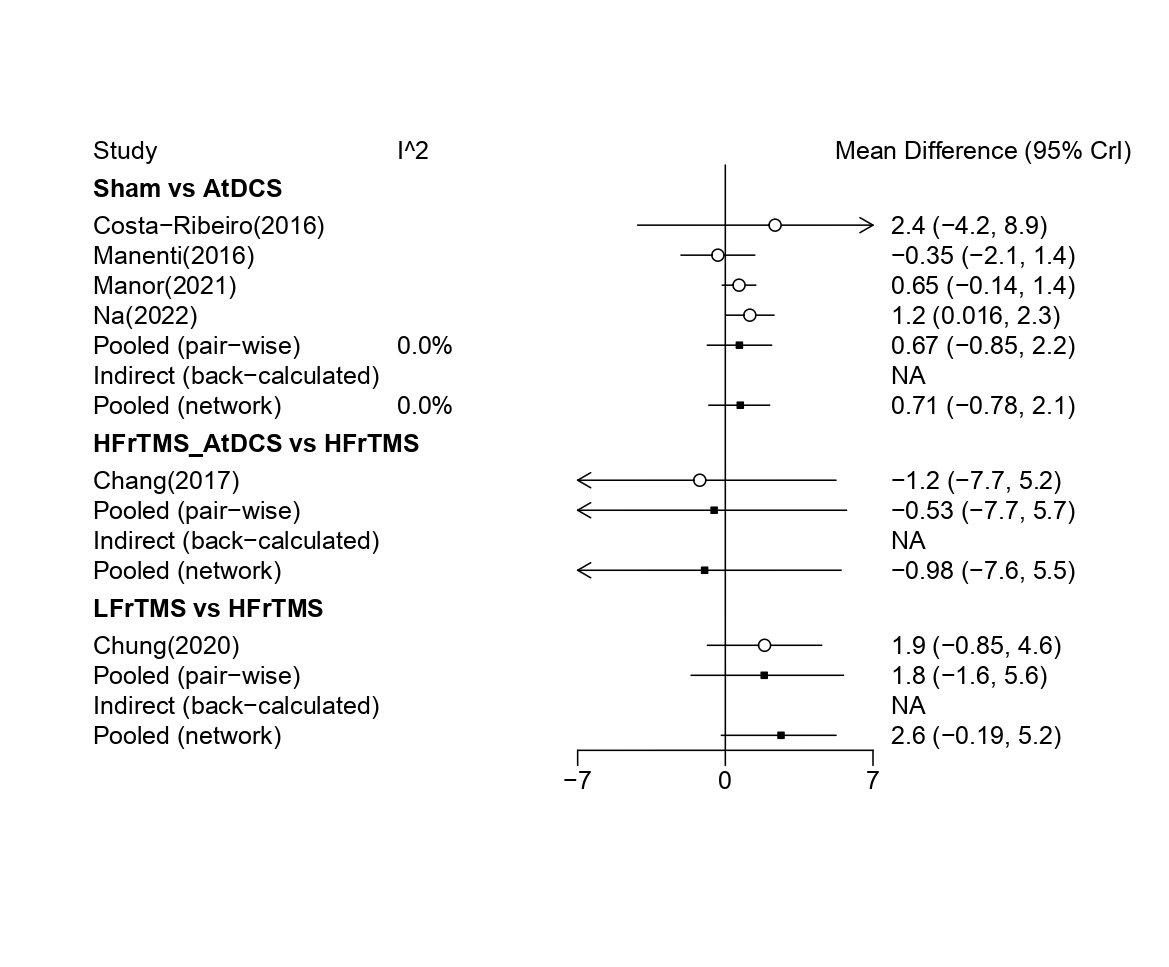


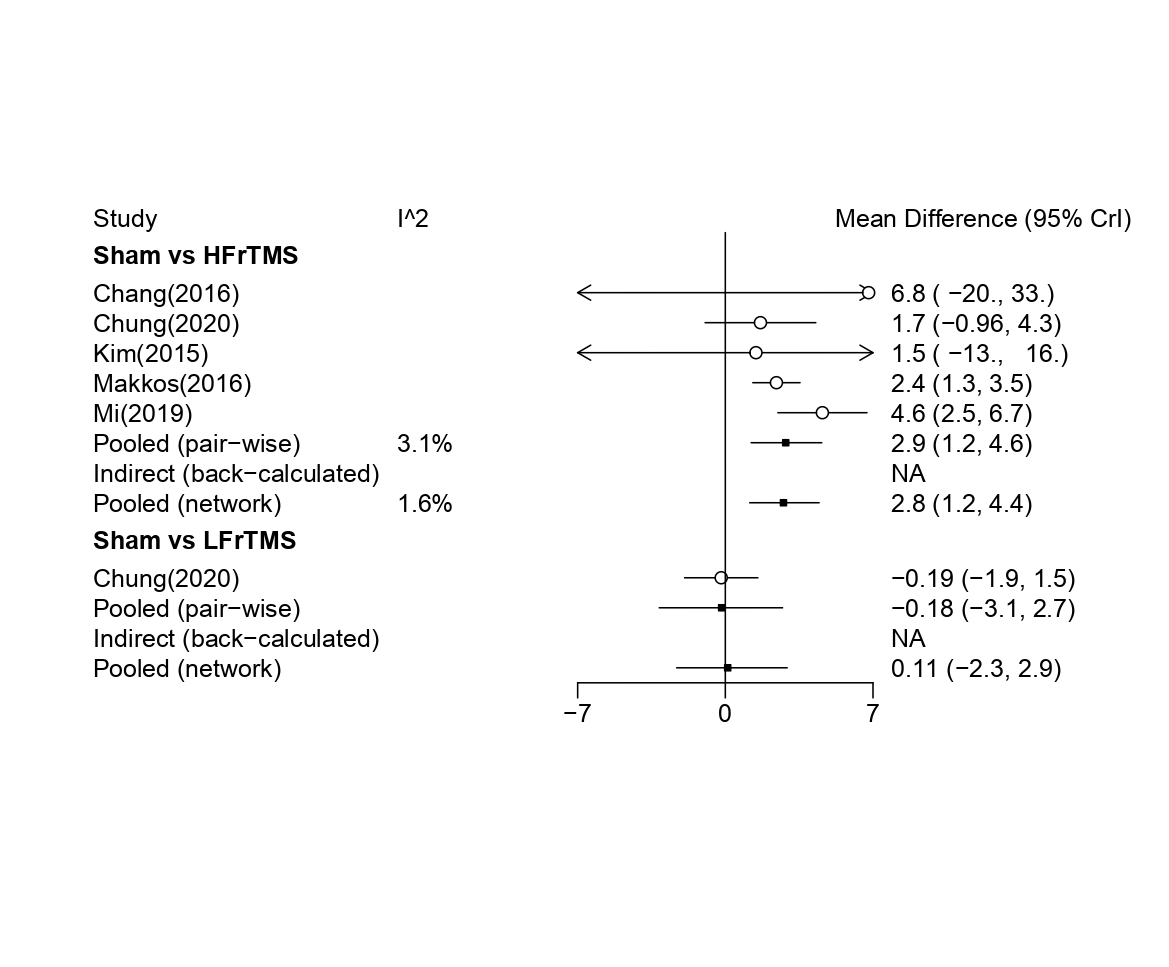


Figure S11 Forest plots for the heterogeneity: short-term FOG.


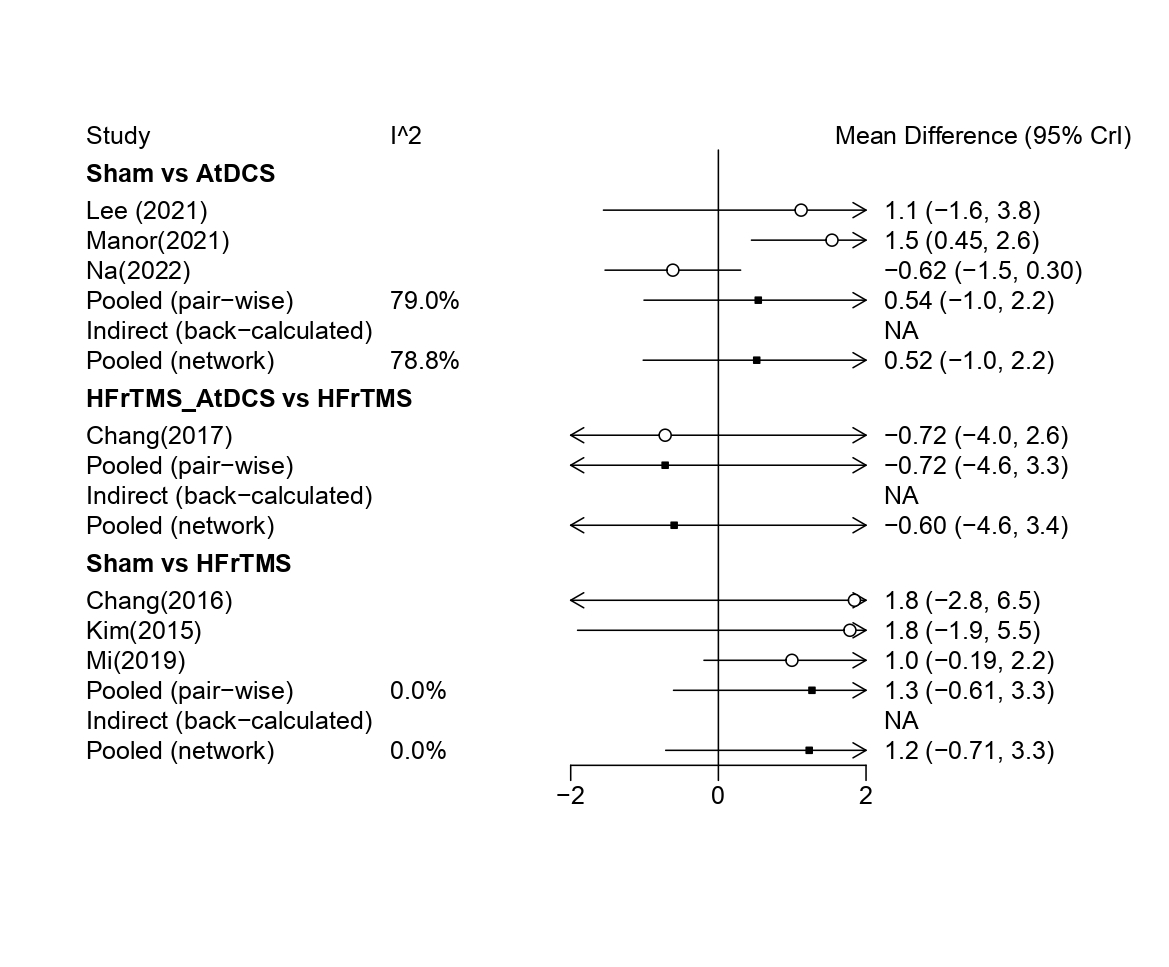


Figure S12 Forest plots for the heterogeneity: long-term FOG.


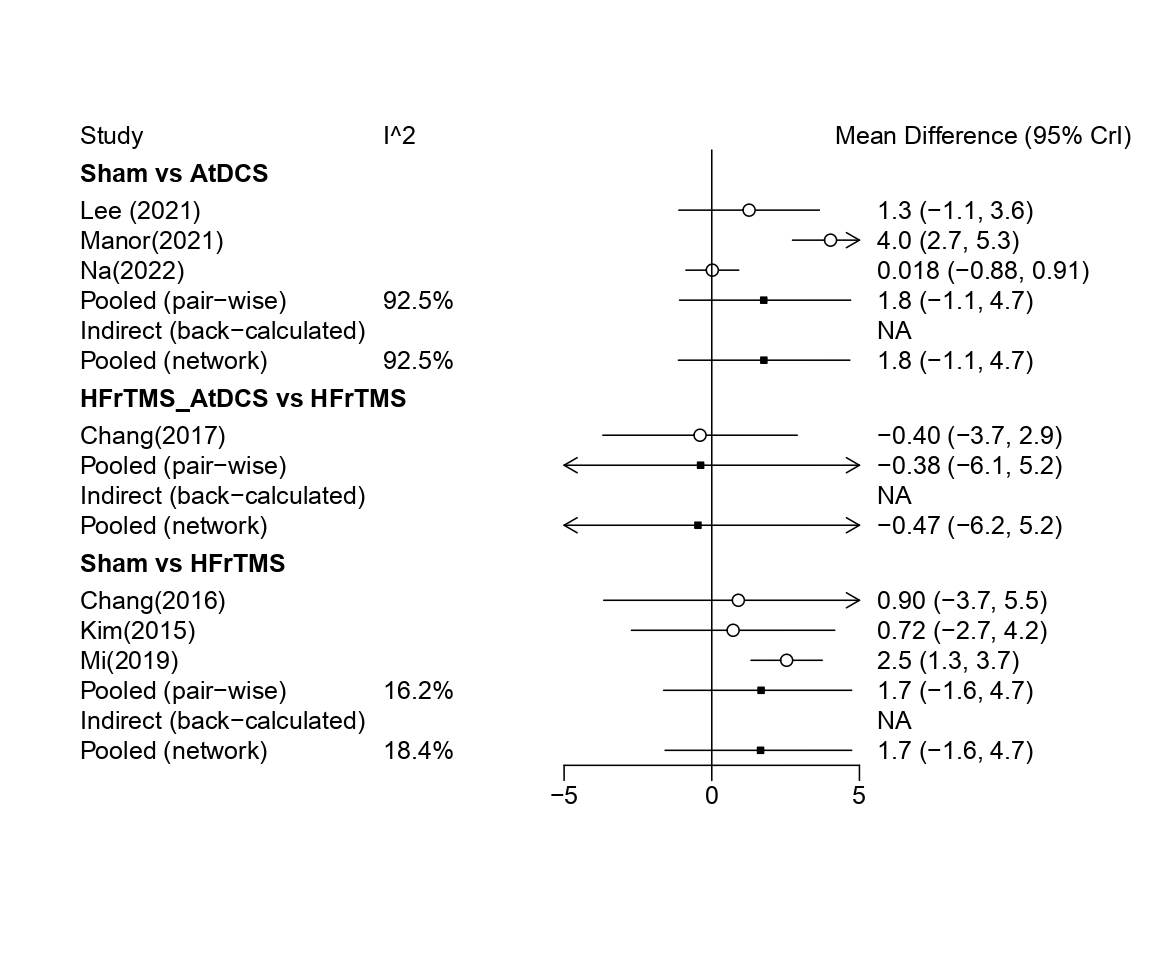


Figure S13 Trace and density of the network meta-analysis: short-term UPDRS-Ⅲ.


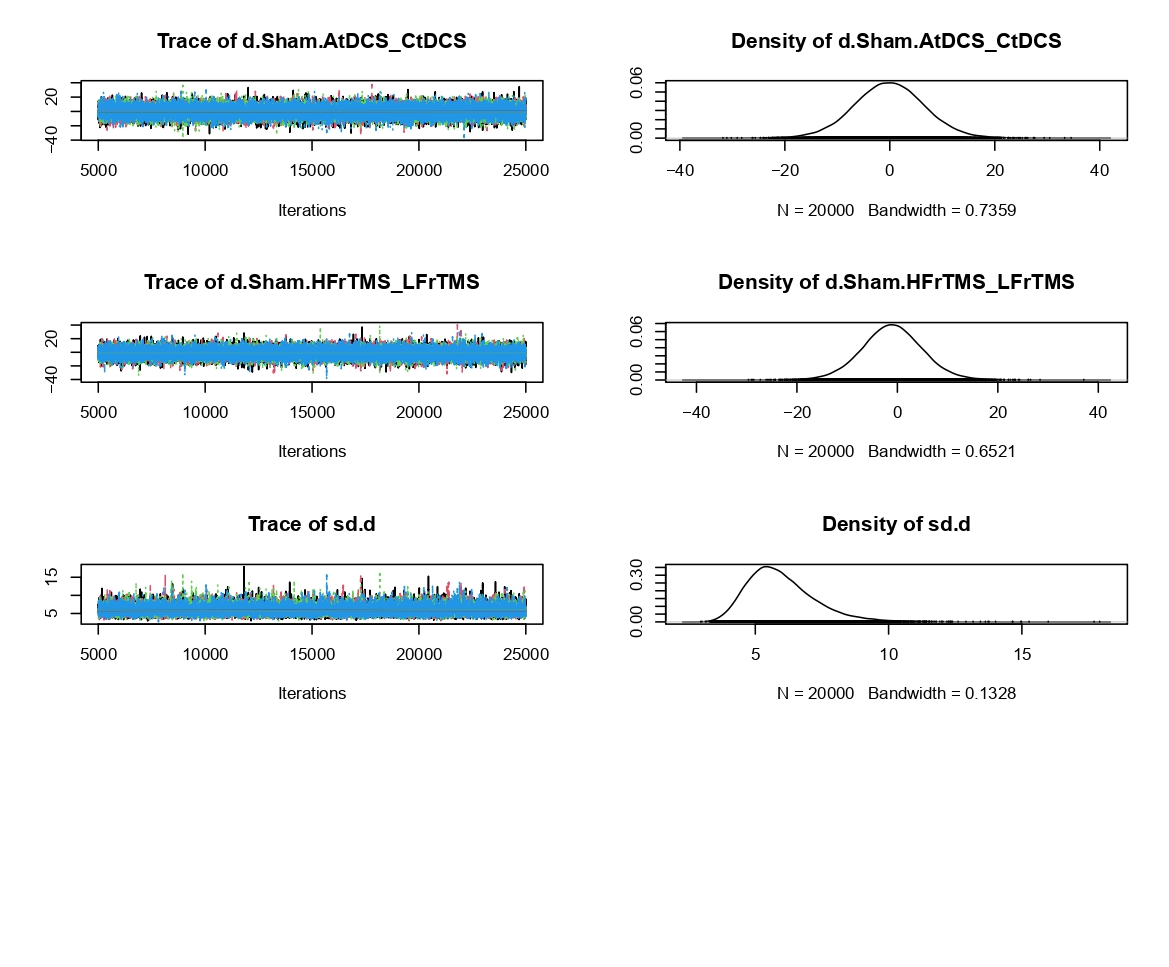


Figure S14 Trace and density of the network meta-analysis: long-term UPDRS-Ⅲ.


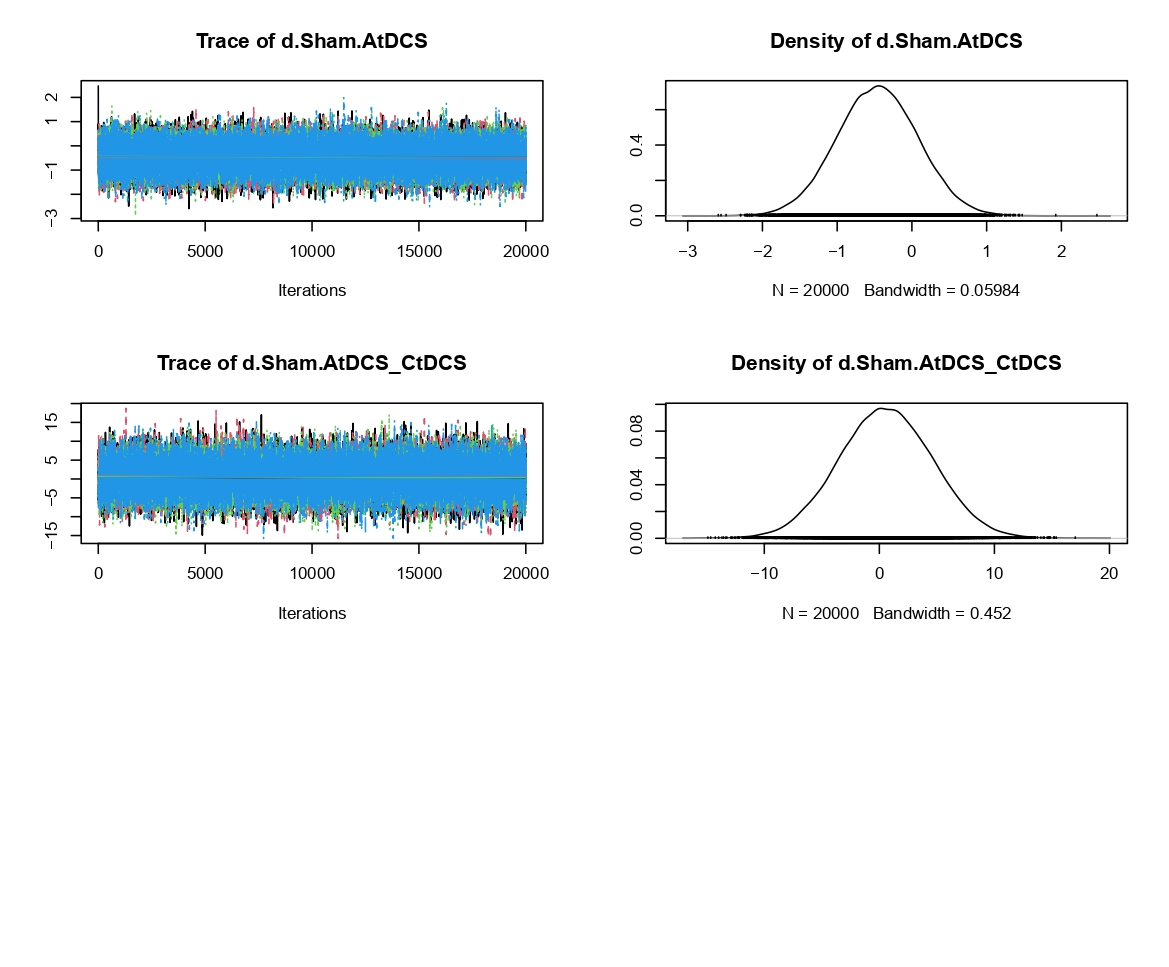


Figure S15 Trace and density of the network meta-analysis: short-term TUG.


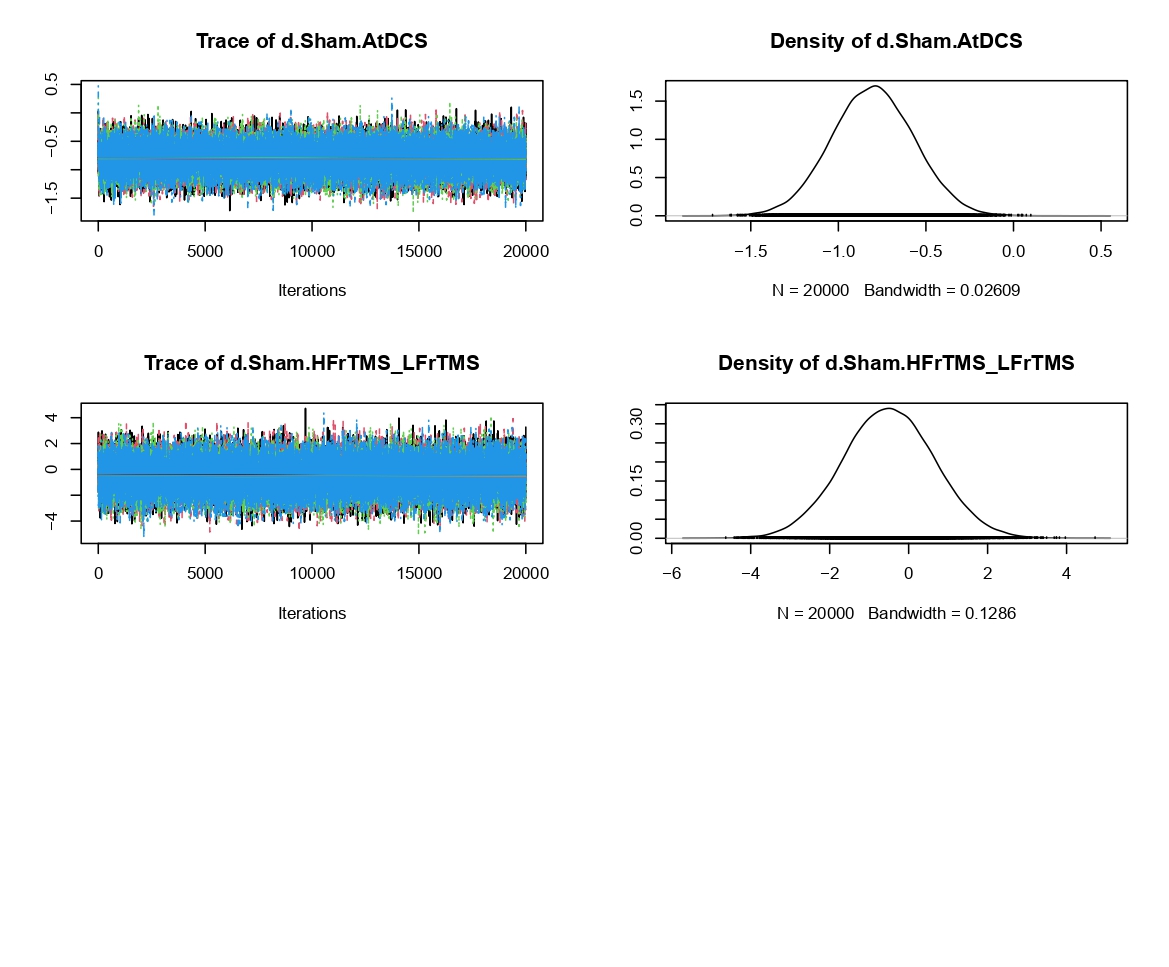


Figure S16 Trace and density of the network meta-analysis: long-term TUG.


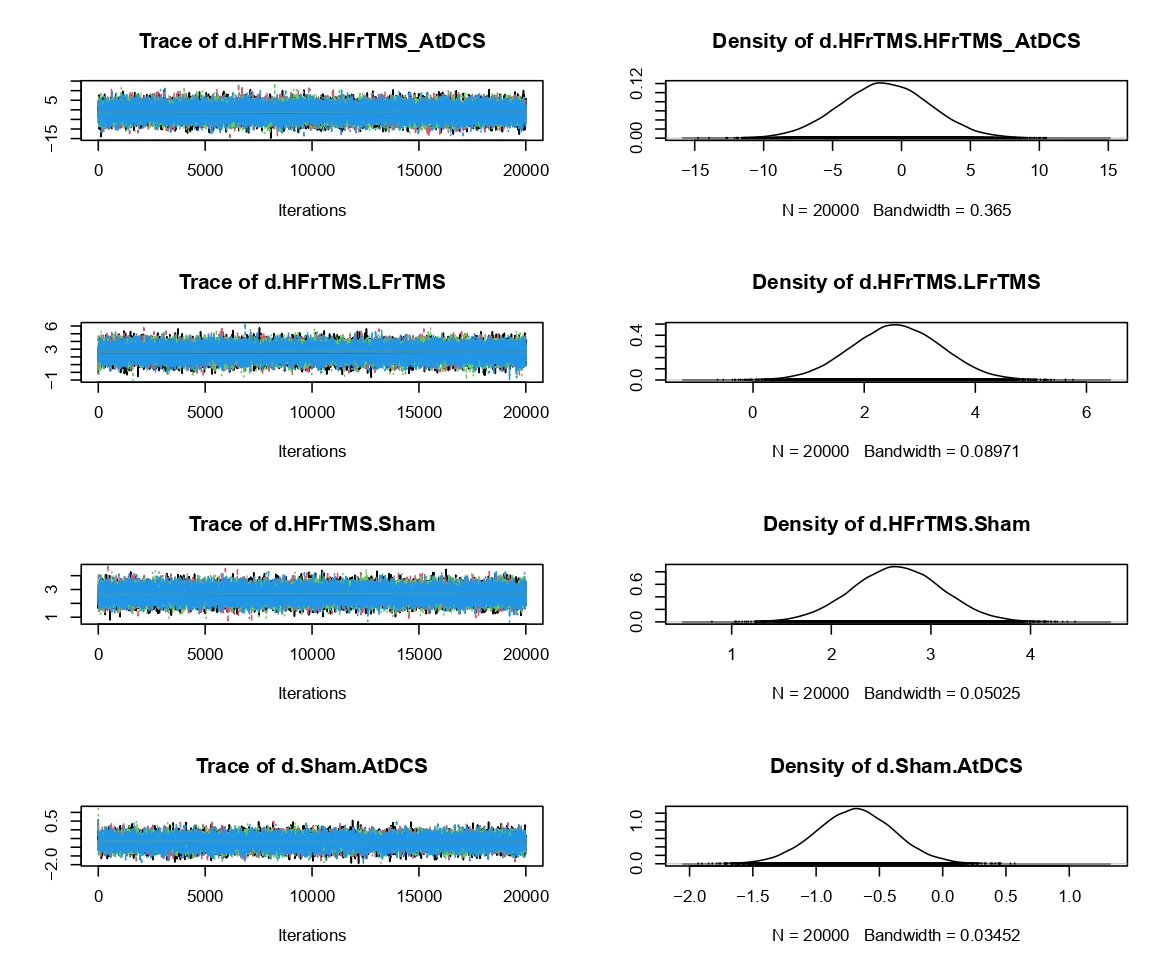


Figure S17 Trace and density of the network meta-analysis: short-term FOG.


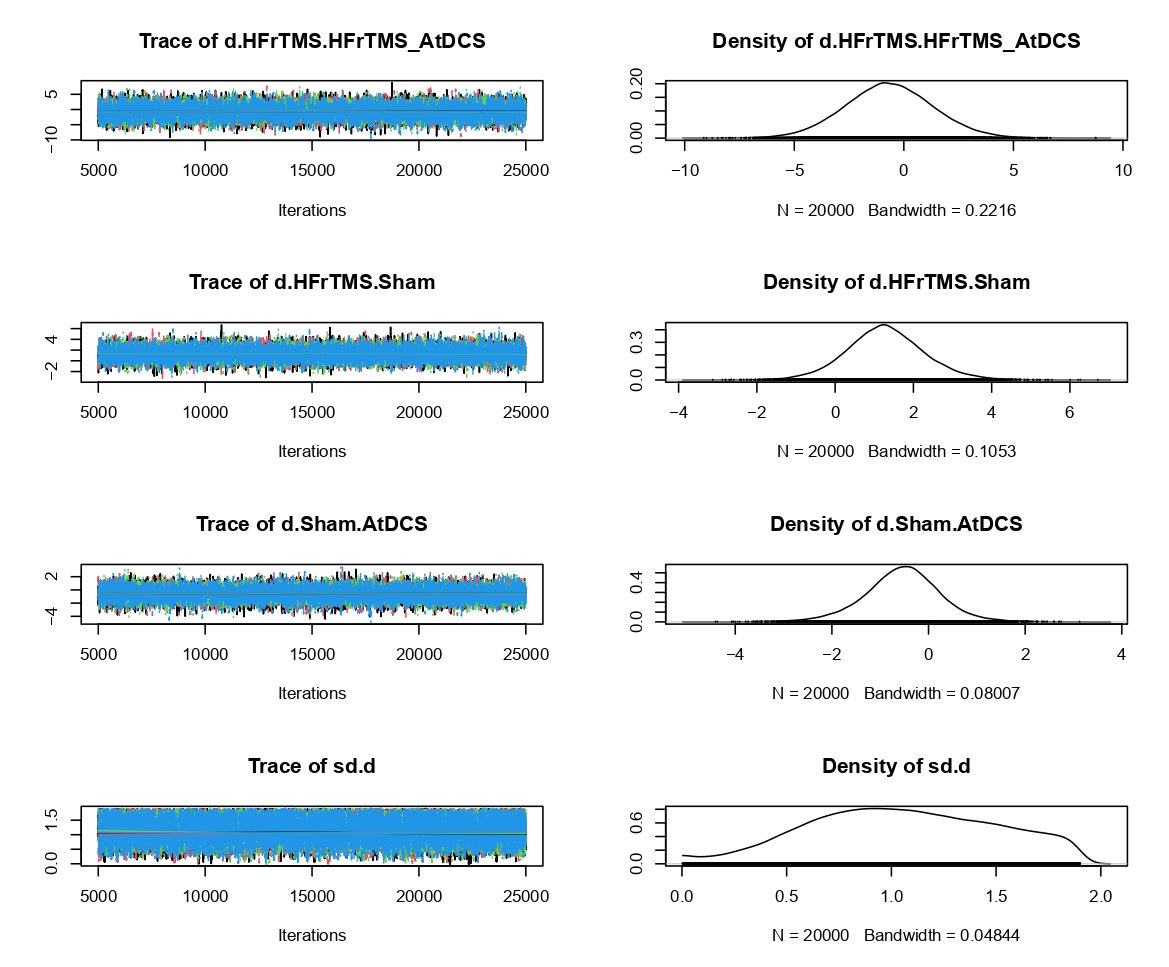


Figure S18 Trace and density of the network meta-analysis: long-term FOG.


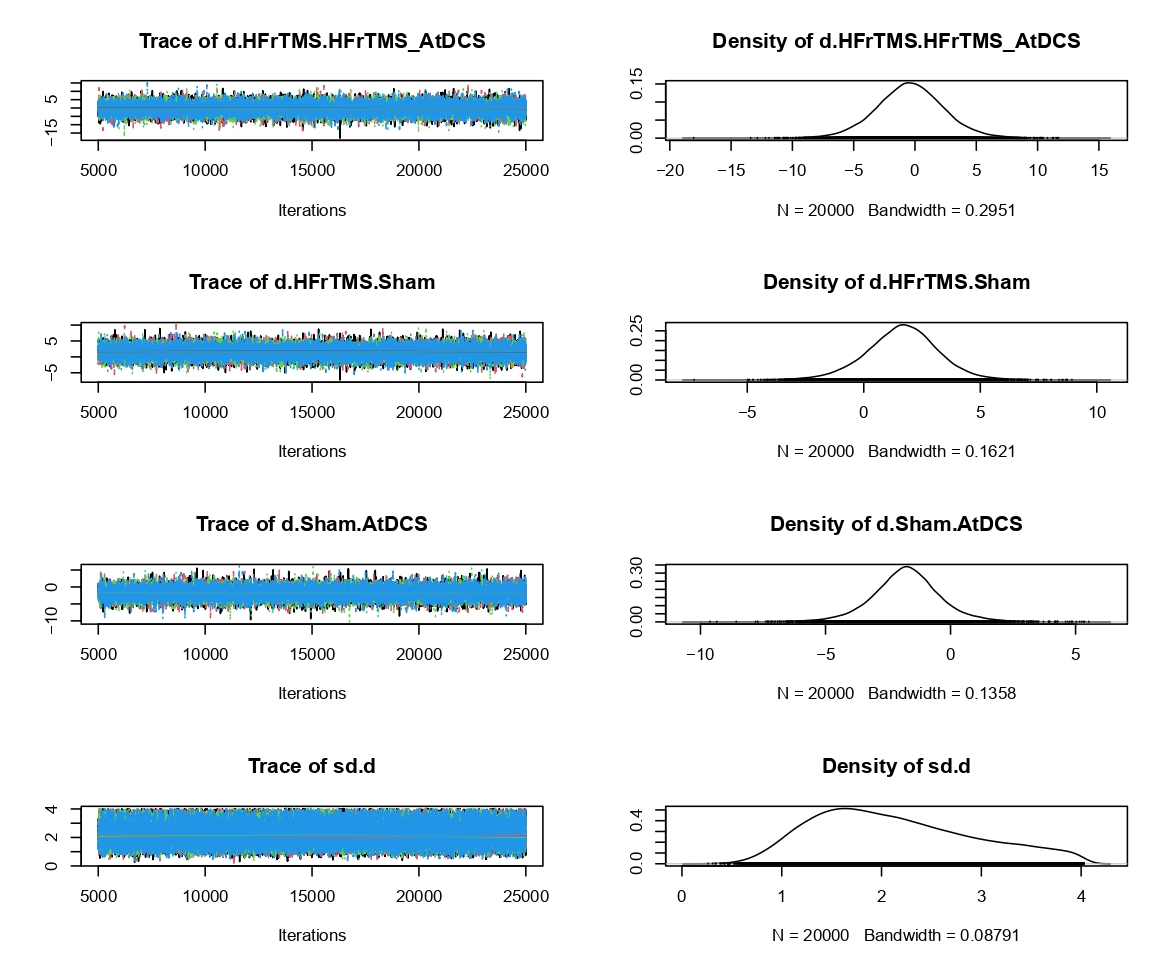


Figure S19 Forest plots for the consistency: short-term UPDRS-Ⅲ.


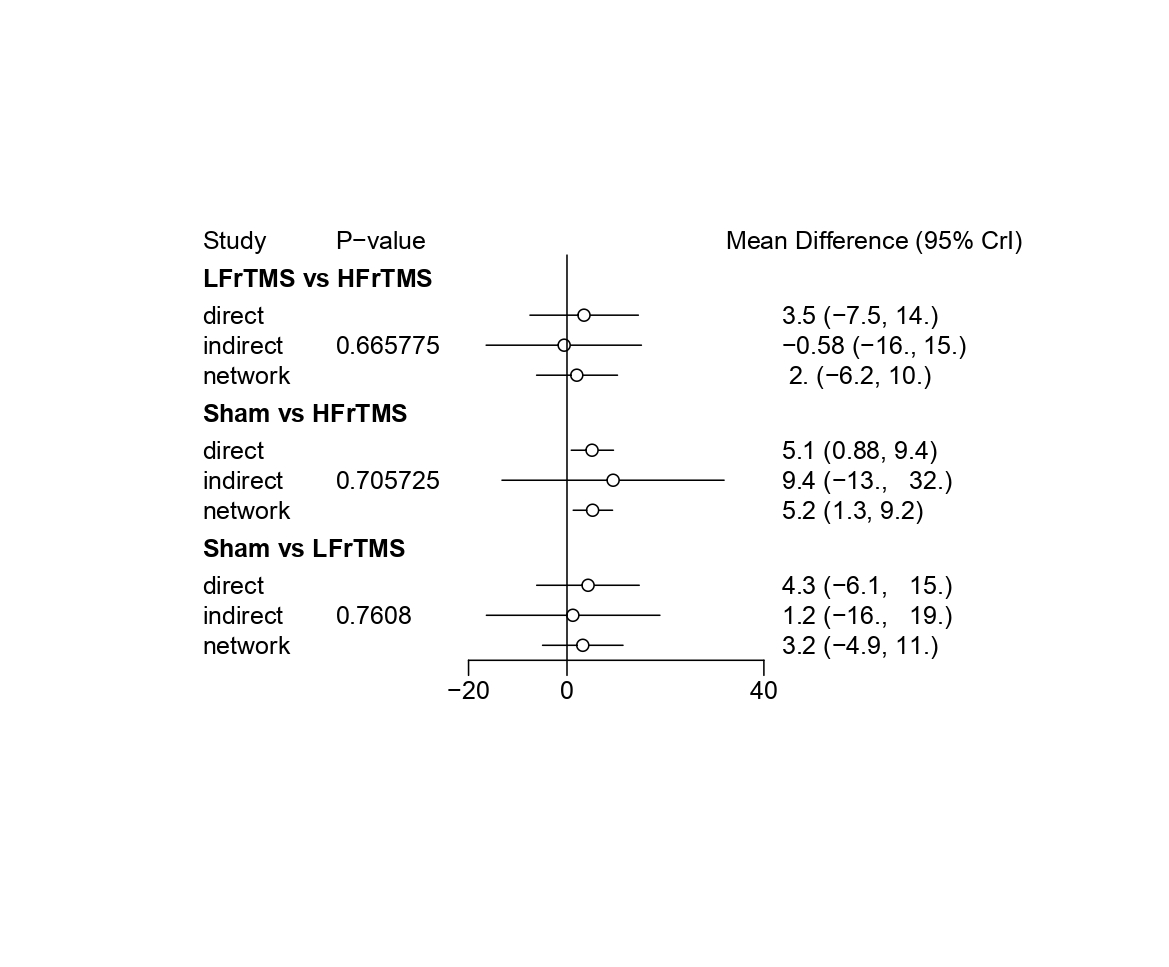


Figure S20 Forest plots for the consistency: long-term UPDRS-Ⅲ.


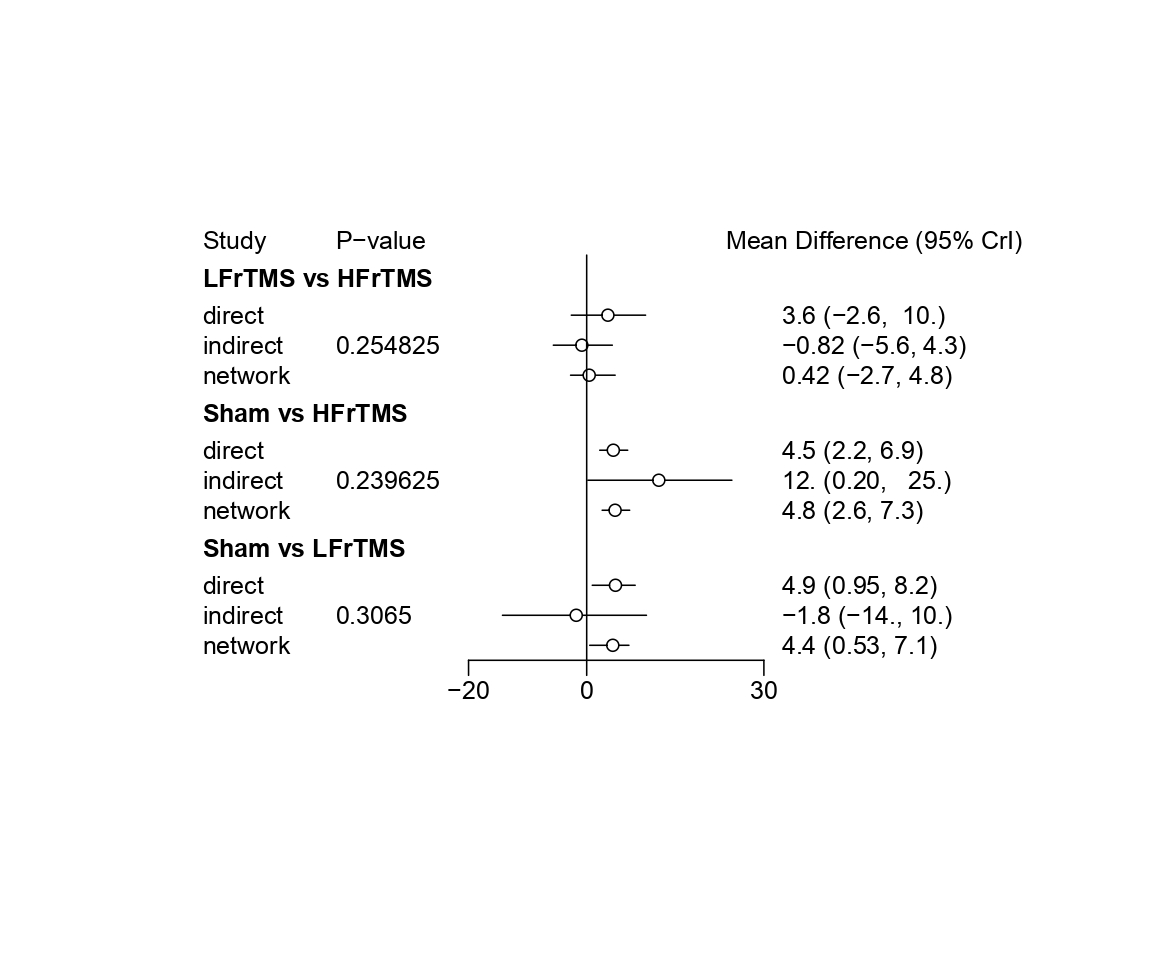


Figure S21: Funnel plot for short-term UPDRS-Ⅲ.


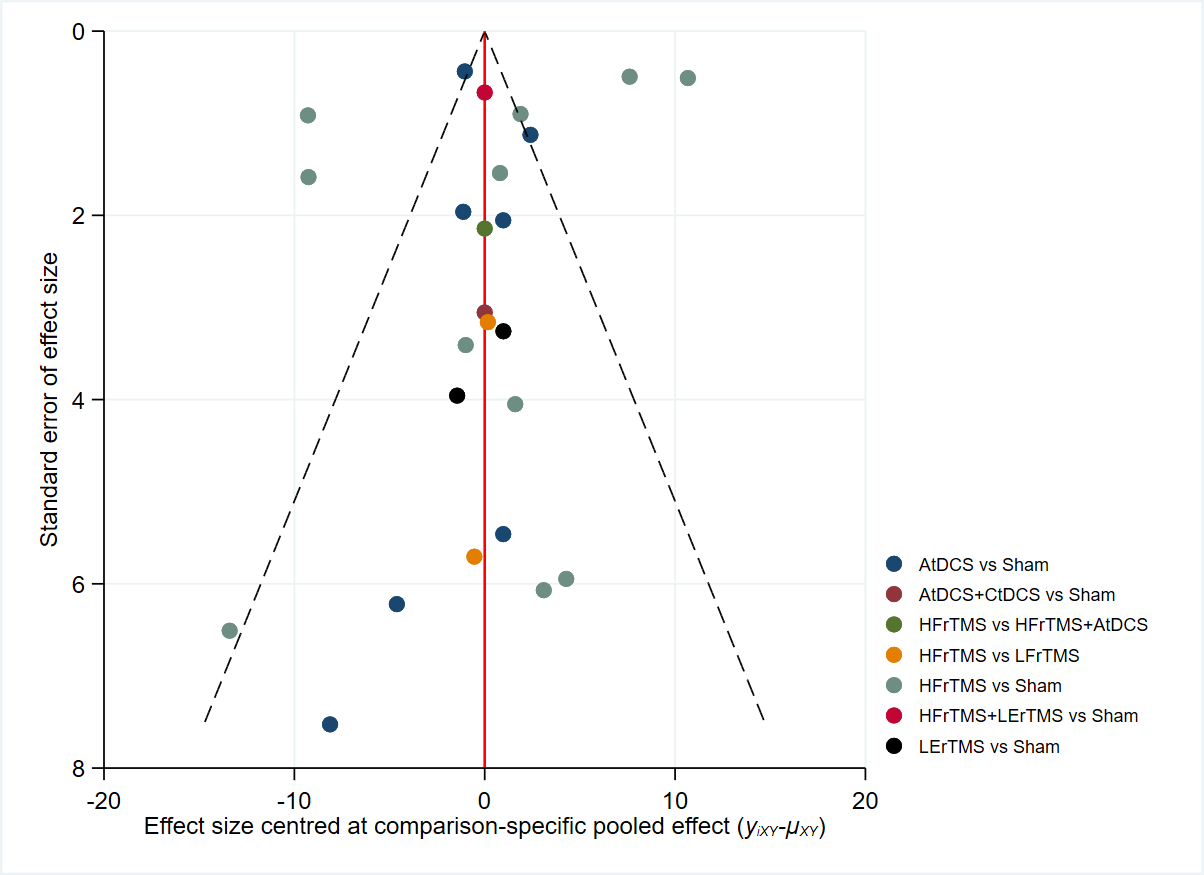


Figure S22: Funnel plot for long-term UPDRS-Ⅲ.


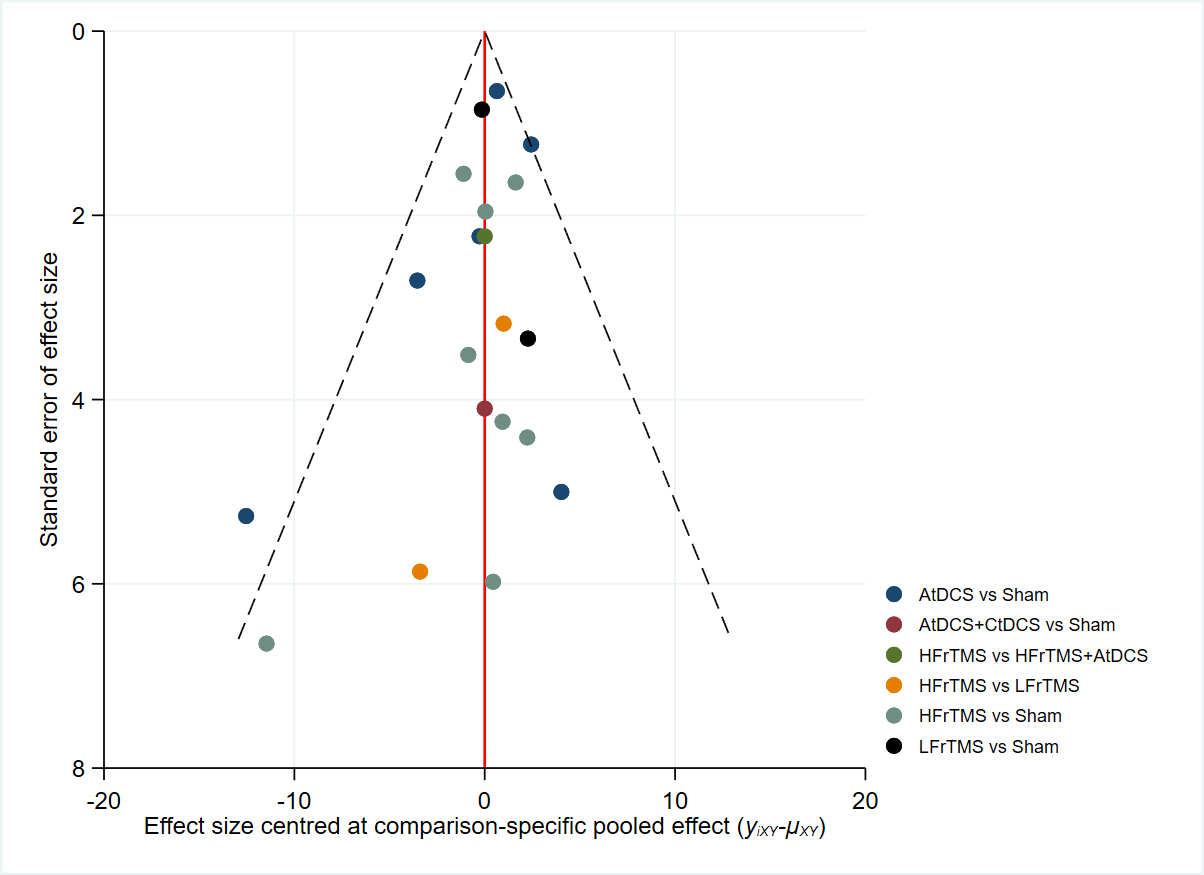


Figure S23: Funnel plot for short-term TUG.


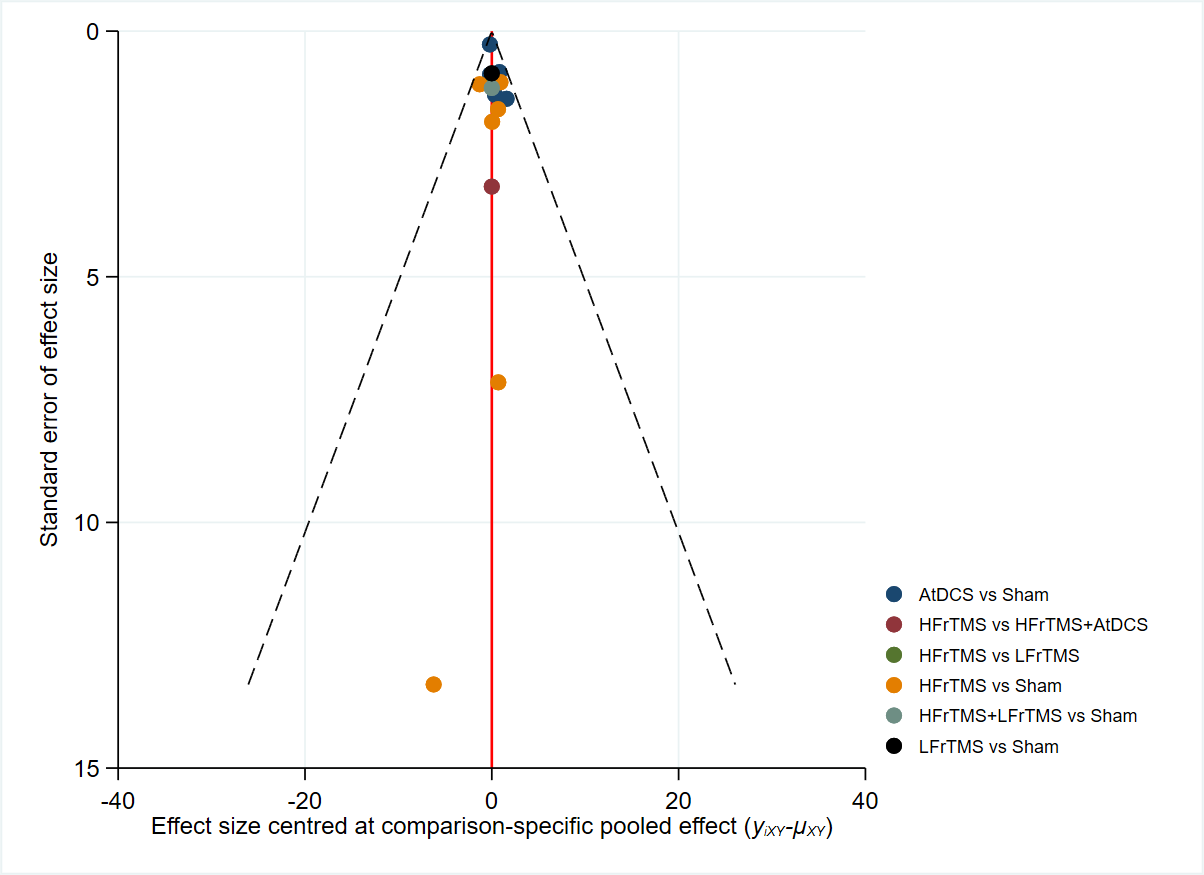


Figure S24: Funnel plot for long-term TUG.


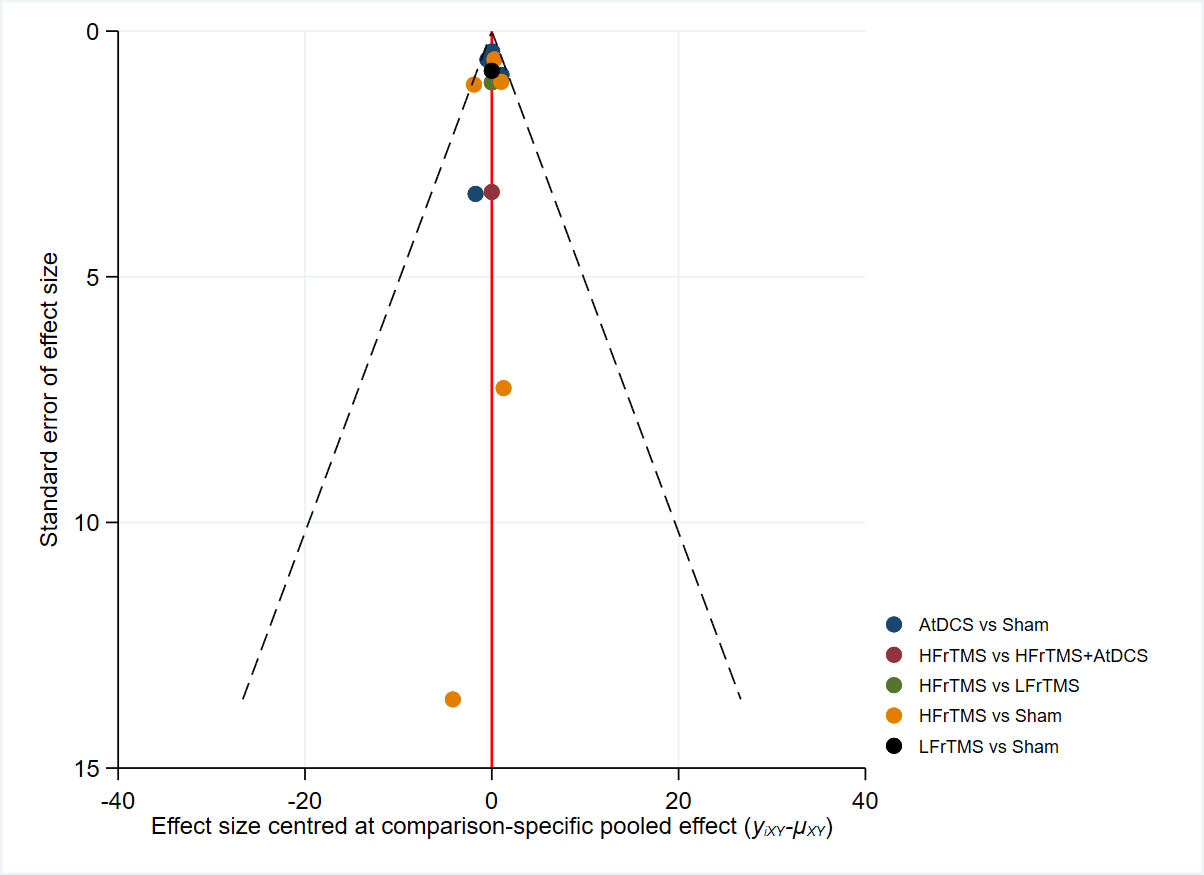


Figure S25: Funnel plot for short-term FOG.


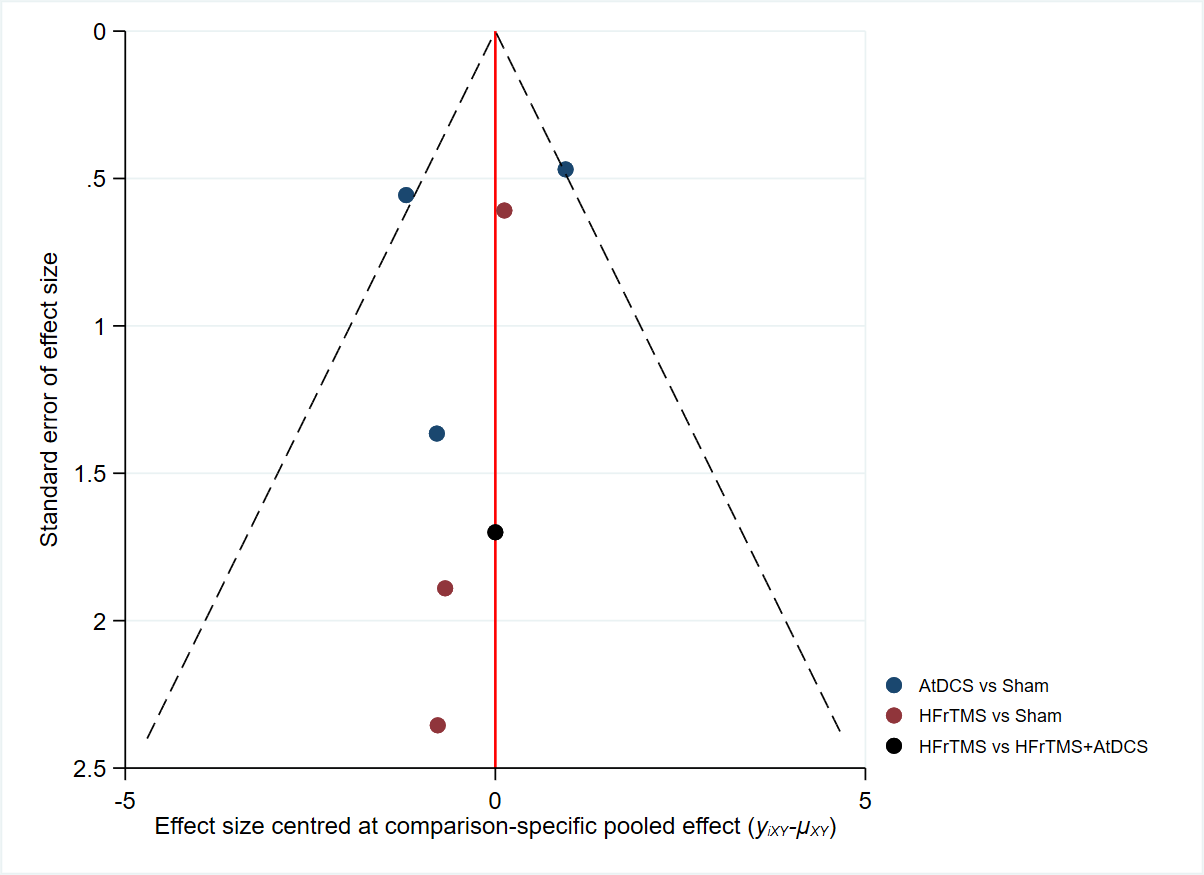


Figure S26: Funnel plot for long-term FOG.


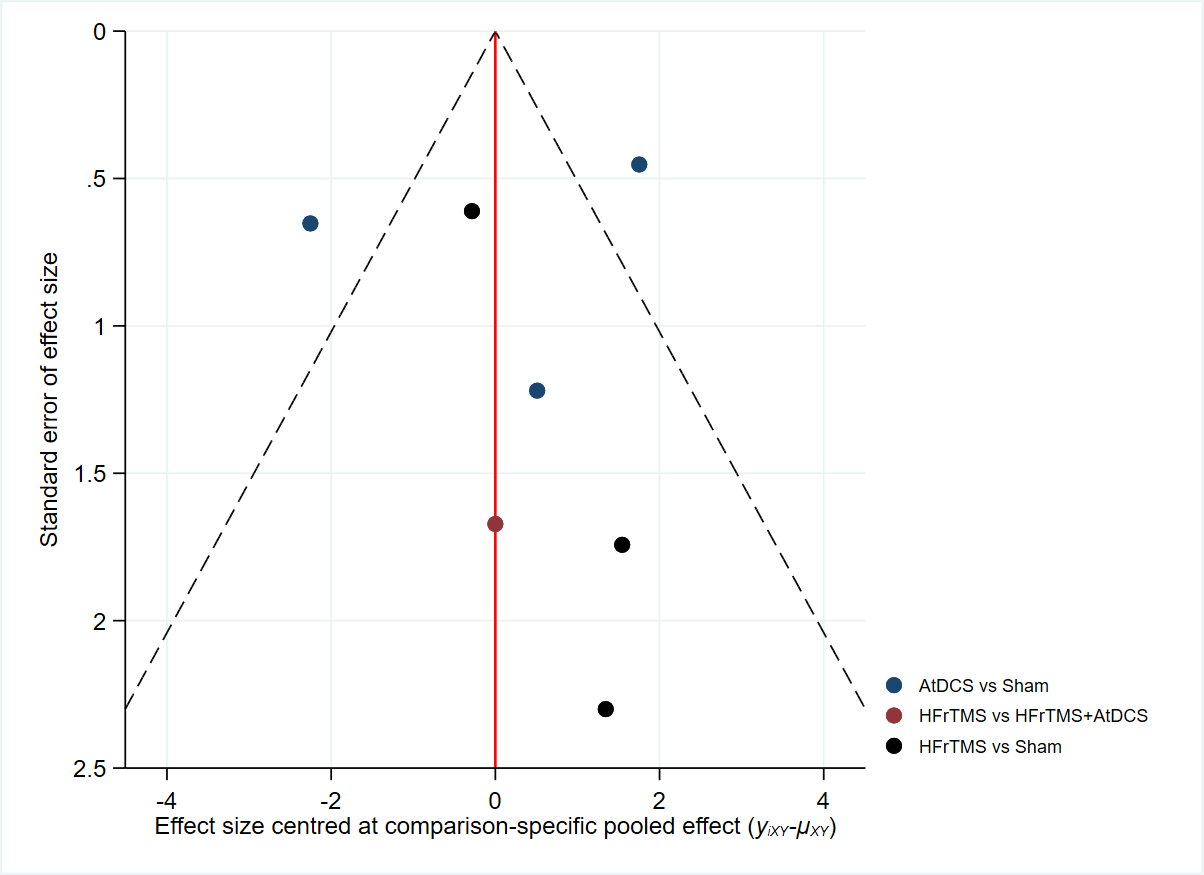


Figure S27: Pair-wise meta-analysis of short-term: UPDRS-Ⅲ, MC, NIBS versus Sham.


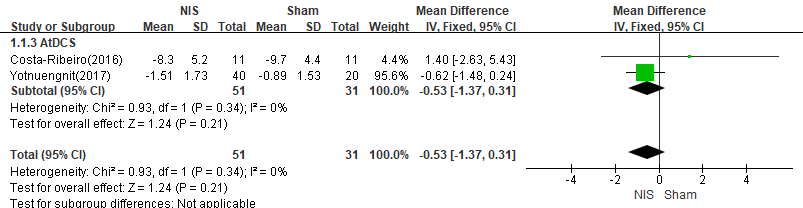


Figure S28: Pair-wise meta-analysis of short-term: UPDRS-Ⅲ, M1, NIBS versus Sham.


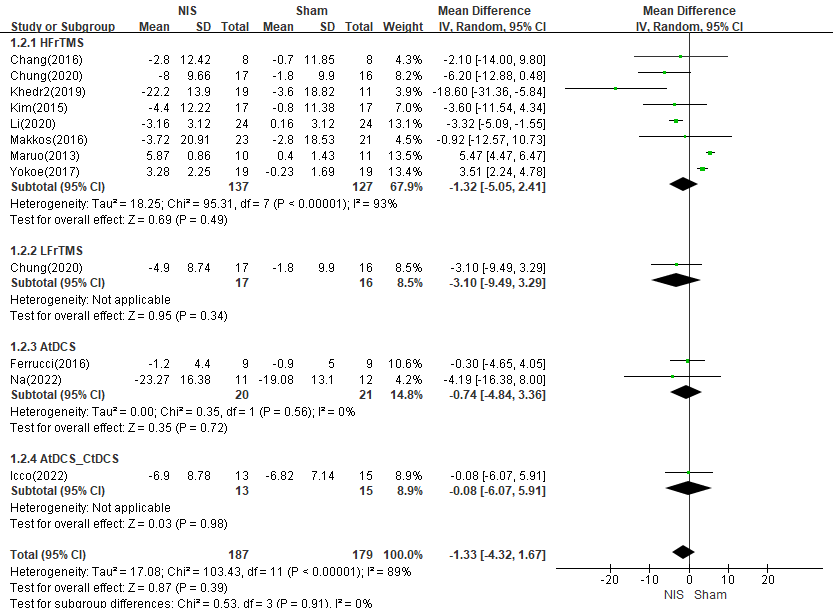


Figure S29: Pair-wise meta-analysis of short-term: UPDRS-Ⅲ, SMA, NIBS versus Sham.


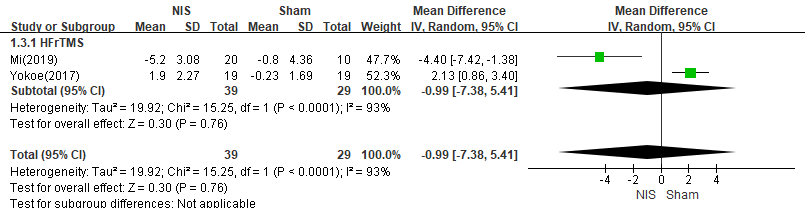


Figure S30: Pair-wise meta-analysis of short-term: UPDRS-Ⅲ, DLPFC, NIBS versus Sham.


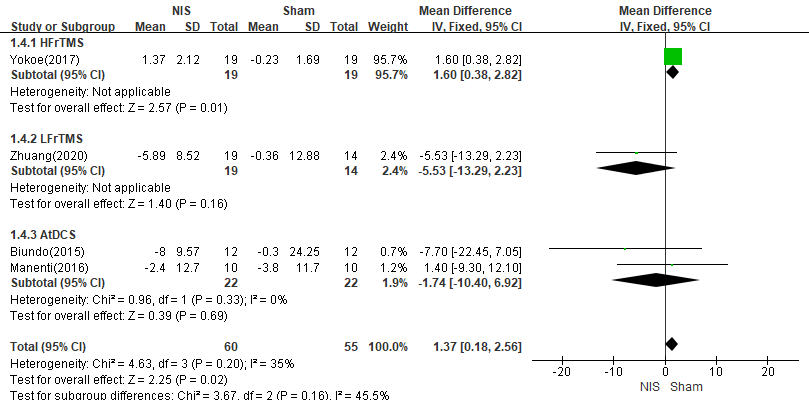


Figure S31: Pair-wise meta-analysis of short-term: UPDRS-Ⅲ, M1_DLPFC, NIBS versus Sham.


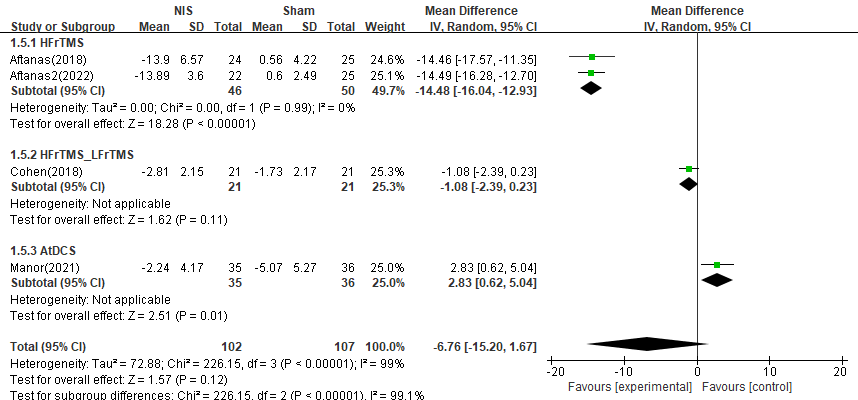


Figure S32: Pair-wise meta-analysis of short-term: UPDRS-Ⅲ, Cerebellum, NIBS versus Sham.


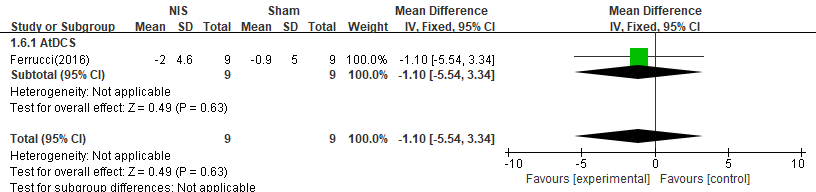


Figure S33: Pair-wise meta-analysis of short-term: TUG, MC, NIBS versus Sham.


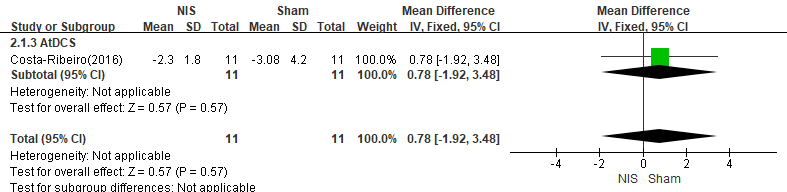


Figure S34: Pair-wise meta-analysis of short-term: TUG, M1, NIBS versus Sham.


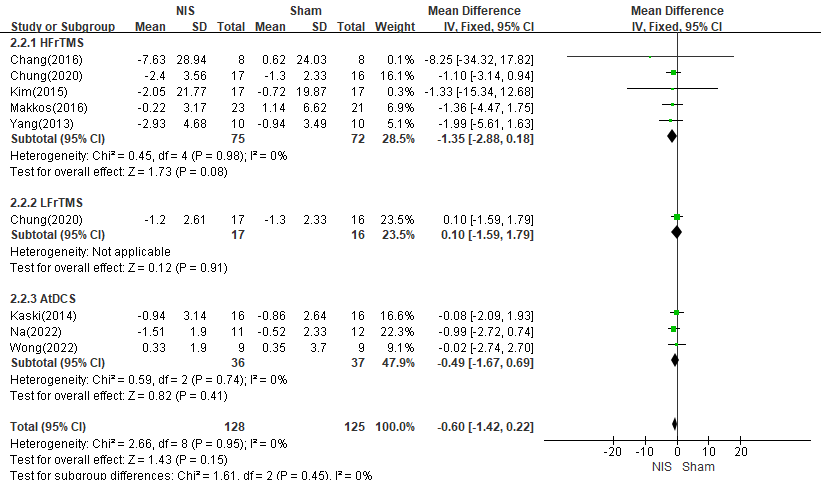


Figure S35: Pair-wise meta-analysis of short-term: TUG, SMA, NIBS versus Sham.


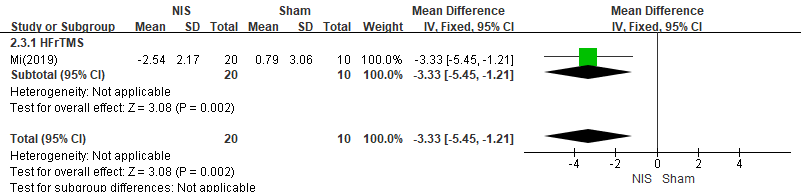


Figure S36: Pair-wise meta-analysis of short-term: TUG, DLPFC, NIBS versus Sham.


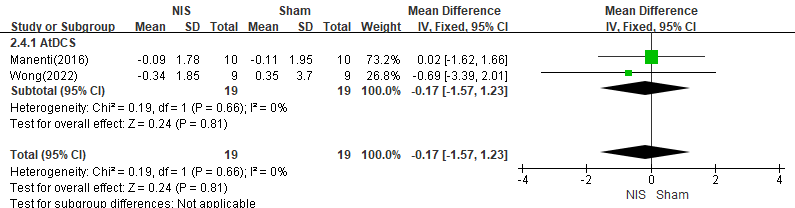


Figure S37: Pair-wise meta-analysis of short-term: TUG, M1_DLPFC, NIBS versus Sham.


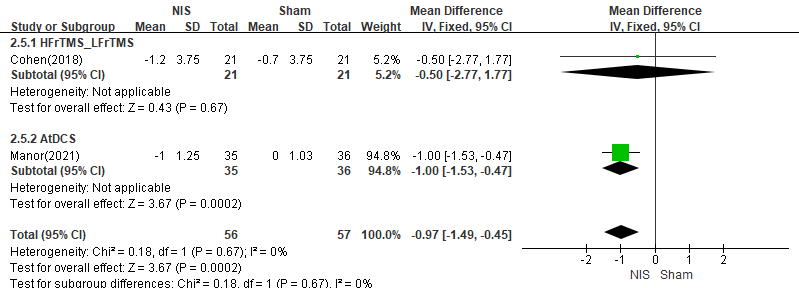


Figure S38: Pair-wise meta-analysis of short-term: TUG, Cerebellum, NIBS versus Sham.


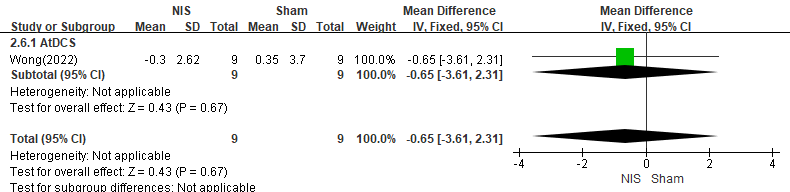


Figure S39: Pair-wise meta-analysis of short-term: FOG, M1, NIBS versus Sham.


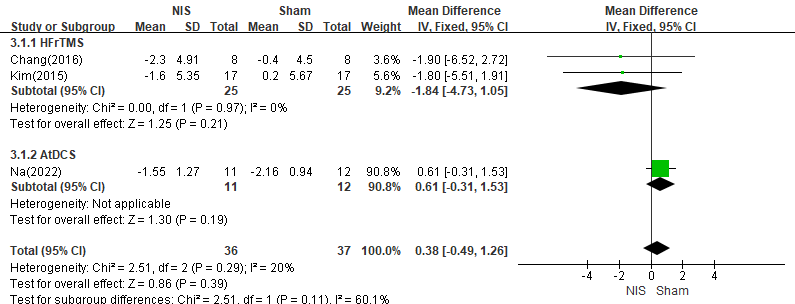


Figure S40: Pair-wise meta-analysis of short-term: FOG, SMA, NIBS versus Sham.


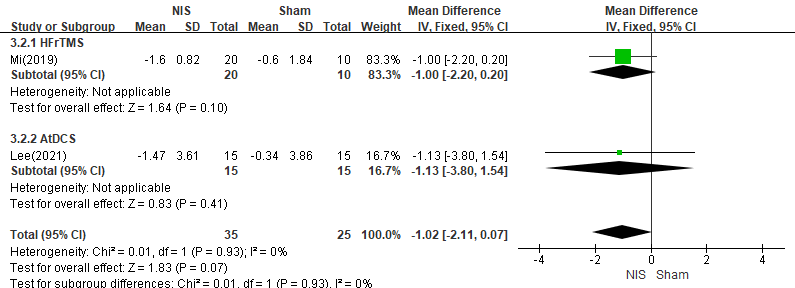


Figure S41: Pair-wise meta-analysis of short-term: FOG, M1_DLPFC, NIBS versus Sham.


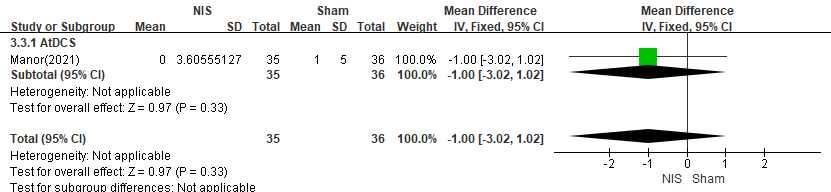


Figure S42: Pair-wise meta-analysis of long-term: UPDRS-Ⅲ, MC, NIBS versus Sham.


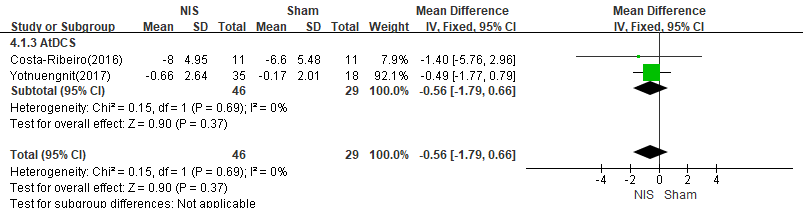


Figure S43: Pair-wise meta-analysis of long-term: UPDRS-Ⅲ, M1, NIBS versus Sham.


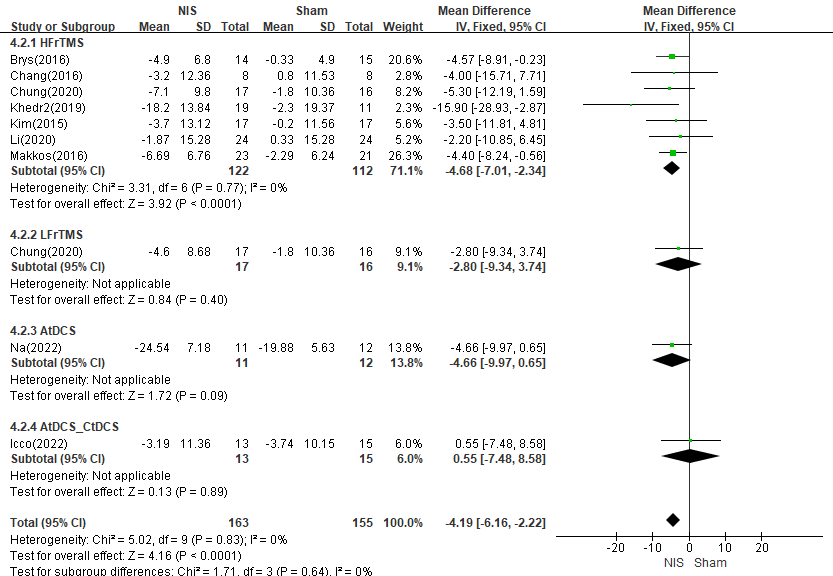


Figure S44: Pair-wise meta-analysis of long-term: UPDRS-Ⅲ, SMA, NIBS versus Sham.


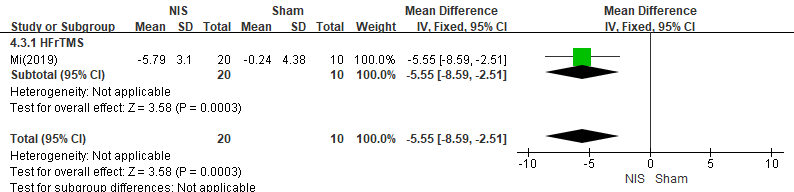


Figure S45: Pair-wise meta-analysis of long-term: UPDRS-Ⅲ, DLPFC, NIBS versus Sham.


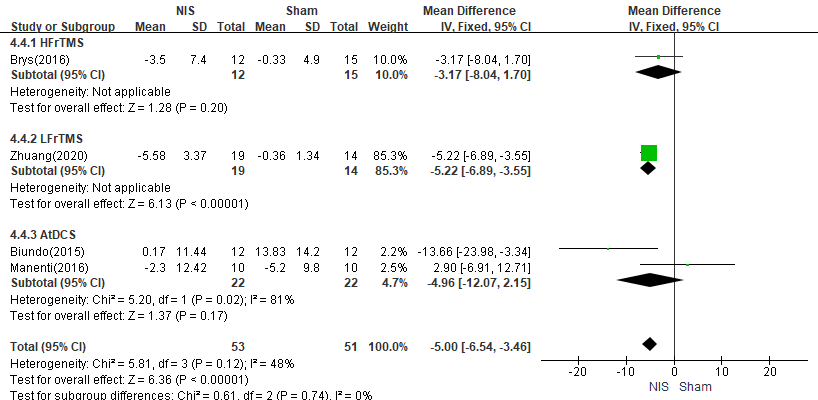


Figure S46: Pair-wise meta-analysis of long-term: UPDRS-Ⅲ, M1_DLPFC, NIBS versus Sham.


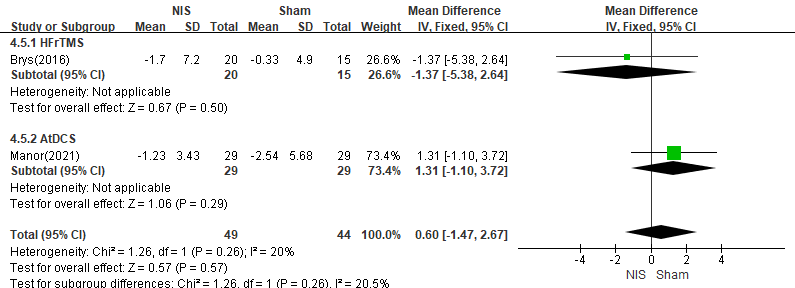


Figure S47: Pair-wise meta-analysis of long-term: TUG, MC, NIBS versus Sham.


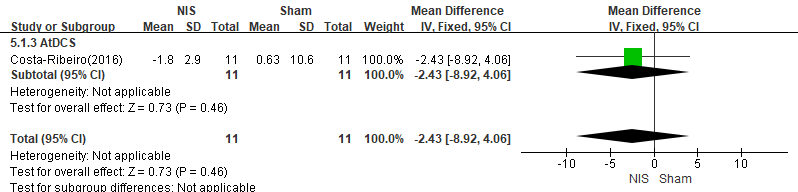


Figure S48: Pair-wise meta-analysis of long-term: TUG, M1, NIBS versus Sham.


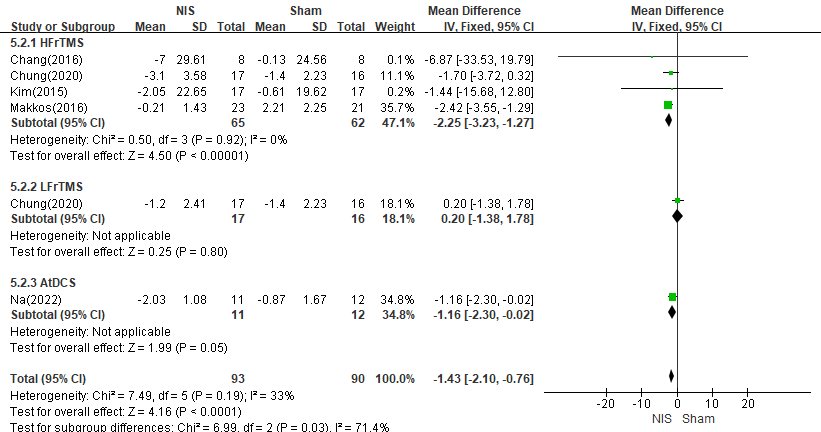


Figure S49: Pair-wise meta-analysis of long-term: TUG, SMA, NIBS versus Sham.


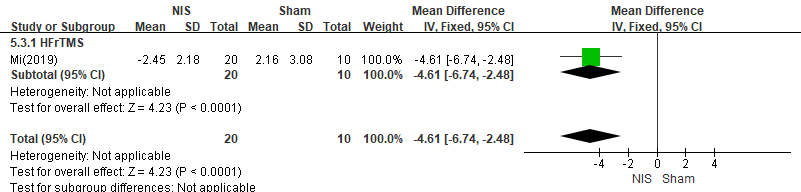


Figure S50: Pair-wise meta-analysis of long-term: TUG, DLPFC, NIBS versus Sham.


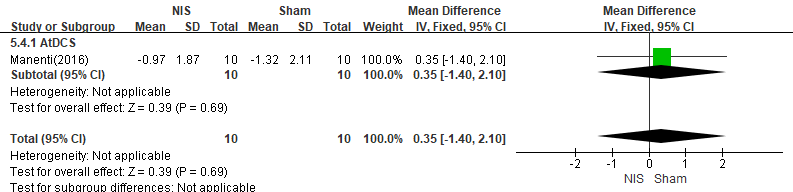


Figure S51: Pair-wise meta-analysis of long-term: TUG, M1_DLPFC, NIBS versus Sham.


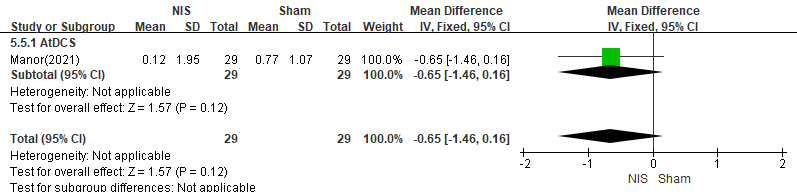


Figure S52: Pair-wise meta-analysis of long-term: FOG, M1, NIBS versus Sham.


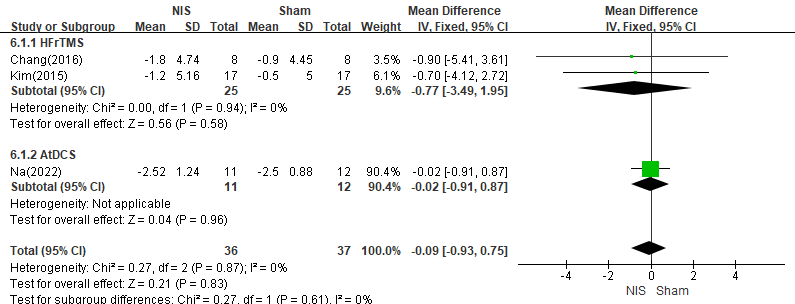


Figure S53: Pair-wise meta-analysis of long-term: FOG, SMA, NIBS versus Sham.


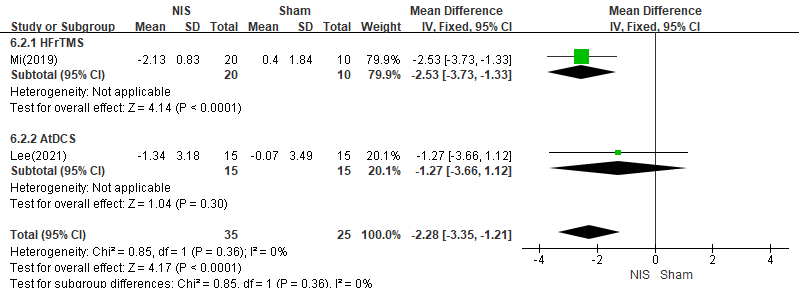


Figure S54: Pair-wise meta-analysis of long-term: FOG, M1_DLPFC, NIBS versus Sham.


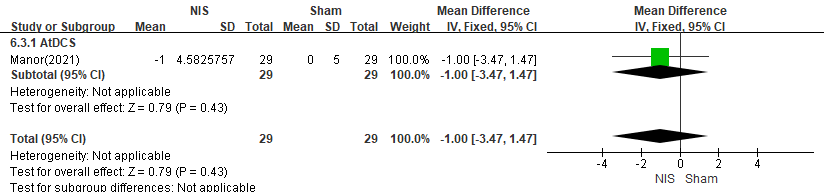


Figure S55: PRSIMA checklist


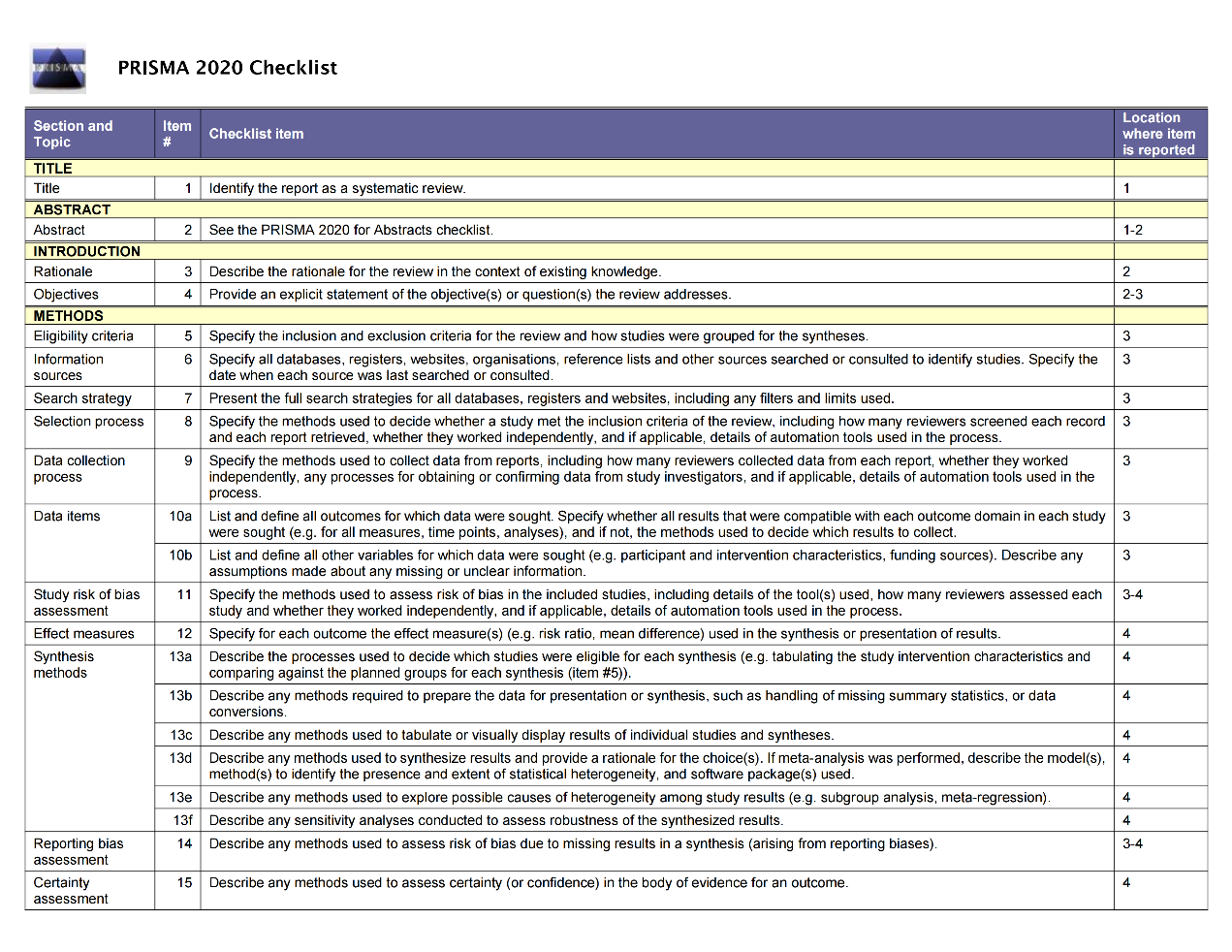


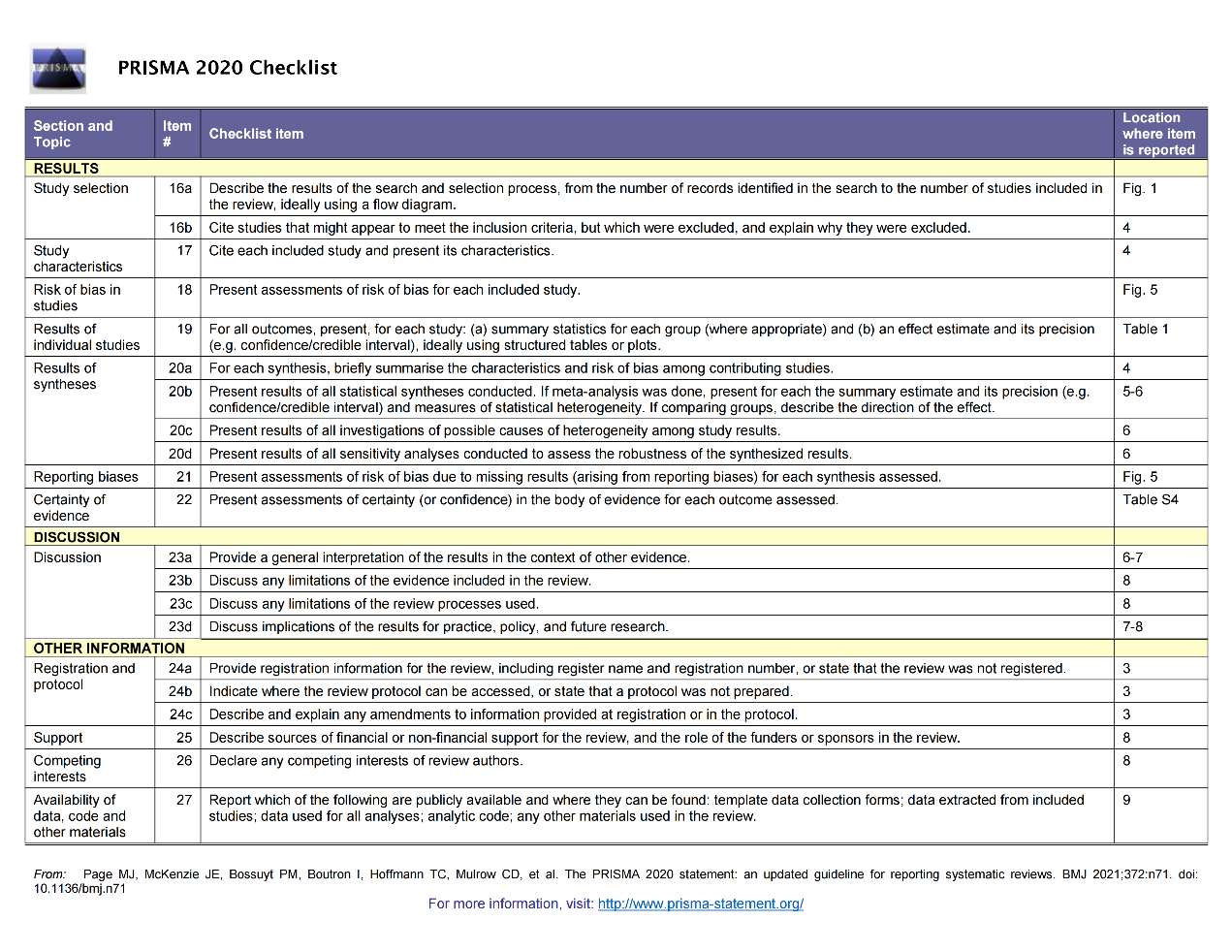

Supplement: Supplementary file 1 [file Data_Sheet_1.docx]
